# A Mechanical Planar Chiral Rotaxane Induces Single‐Handed Helicity in a Twisted Perylene Diimide

**DOI:** 10.1002/anie.1877364

**Published:** 2026-07-31

**Authors:** Thomas E. Lawson, Denis Hartmann, Artemijs Krimovs, Qilong Sun, Robert Pal, Timothy A. Barendt

**Affiliations:** ^1^ School of Chemistry University of Birmingham Birmingham UK; ^2^ Department of Chemistry University of Durham Durham UK

**Keywords:** chiral induction, chirality, chiroptics, perylene diimide, rotaxane

## Abstract

The twisting of chromophores such as perylene diimides (PDIs) into helical structures affords chiroptical properties from discrete molecules, which are of significant value for chiral sensing, imaging and catalysis. Since most helical PDI derivatives are susceptible to racemisation, chiral induction provides a promising, yet underdeveloped, strategy for generating materials with persistent chiroptical properties. For the first time, we use a mechanically planar chiral rotaxane as a configurationally stable chirality source to induce single‐handed helicity in a molecular chromophore, yielding persistent circular dichroism and circularly polarised luminescence. The large π‐surface of the PDI also enables rotaxane self‐assembly, which is rare among mechanically interlocked molecules. While mechanical bonding is shown to influence the geometry of the resulting PDI dimers, mechanical planar chirality directs their handedness, thereby providing control at both the molecular and supramolecular levels.

## Graphical abstract

A mechanical planar chiral rotaxane (***R*_mp_**) is used to induce single‐handed helical chirality (***M***) in a perylene diimide chromophore, resulting in persistent circular dichroism (CD) and circularly polarised luminescence (CPL).

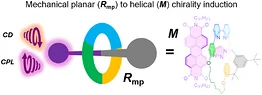

## 1 Introduction

Chiroptical materials are materials that absorb (circular dichroism [CD]) or emit (circularly polarised luminescence [CPL]) circularly polarised light. Amongst organic materials, rylene diimides (RDIs) have become a popular choice of chromophore due to their strong absorption and emissive properties in the visible region, which are tuneable through synthetic modification [1, 2]. Notably, the installation of point chiral groups only results in weak chiroptical properties from the RDI monomer [3], with stronger chiroptical properties often arising from their π–π assisted self‐assembly into extended aggregates exhibiting supramolecular chirality [4]. However, while polymers have many uses as chiroptical materials in the solid‐state [5, 6], they are less desirable for applications requiring a discrete chiral chromophore in solution, such as for chiral sensing [7], imaging [8] or catalysis [9].

**FIGURE 1:**
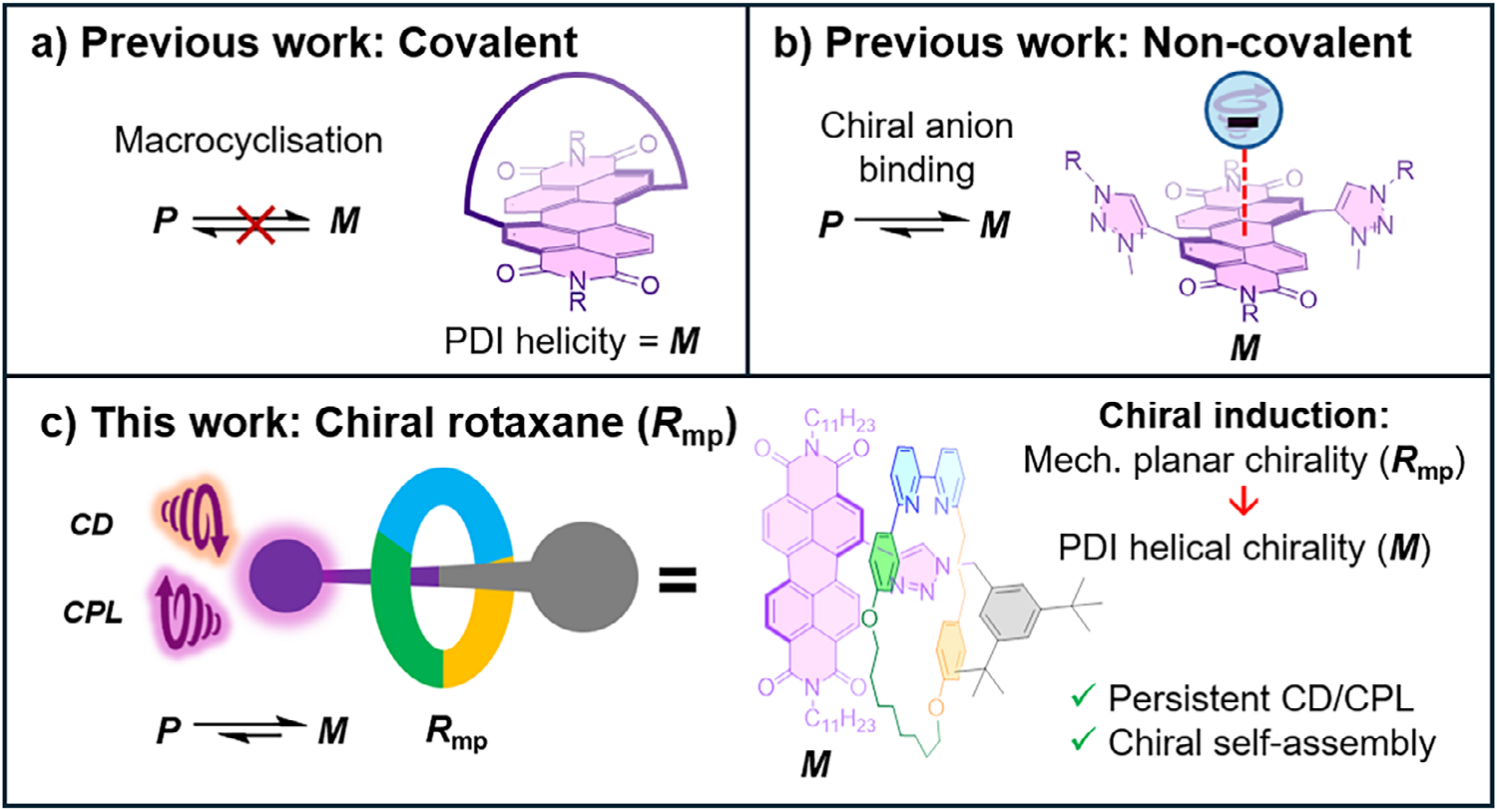
(a) Previous covalent [14] and (b) non‐covalent [18] strategies to ***M/P*** helical PDIs with persistent chiroptical properties and (c) a new approach using a mechanical planar chiral rotaxane (e.g., ***R*_mp_**) to induce single‐handed PDI helicity (*M*).

Importantly, intrinsic chirality can arise in a discrete chromophore from its helical twist, generating two enantiomers, ***M***/***P*** [10]. For larger RDIs, such as perylene dimiides (PDIs), twisting occurs when the aromatic core is substituted at the bay positions (Figure 1a) [2]. However, most helical PDIs are not configurationally stable [11], with their racemisation leading to the loss of chiroptical properties. While the covalent functionalisation of PDI [11, 12, 13], exemplified by macrocycle strapping across the bay positions (Figure 1a) [14, 15], can lock helicity and enable enantiomer resolution, a complementary strategy is to bias the ***M*** ⇄ ***P*** conformational equilibrium using a stable chiral source. Unfortunately, point‐chiral substituents are generally ineffective at inducing intrinsic chirality in monomeric PDIs [16, 17]. Therefore, we previously developed a non‐covalent approach in which a chiral anion induces single‐handedness in a helical PDI (Figure 1b) [18]. However, because this chiral induction mechanism relies on intermolecular interactions between the PDI and the chiral source, it remains reversible and is susceptible to reductions in the CD/CPL signal when environmental factors, including temperature, solvent and concentration, weaken these interactions.

In this work, we integrated a dynamic, helical PDI (***M*** ⇄ ***P***) into a configurationally stable chiral environment, specifically a mechanical planar chiral rotaxane (***S*_mp_** or ***R*_mp_**, Figure 1c). In a mechanical planar chiral rotaxane, the spatial arrangement of axle and macrocycle components generates two enantiomers, ***S*_mp_**/***R*_mp_**, that cannot interconvert without breaking the mechanical bond [19]. In our system, the rotaxane provides both a persistent source of chirality and a robust intramolecular mechanism for chiral induction to the twisted PDI chromophore, via the mechanical bond (Figure 1c). While rotaxanes have been used to control optical properties [20, 21, 22], tuning chiroptics with mechanical planar chiral interlocked molecules is less common [23, 24]. To the best of our knowledge, mechanical planar chirality has only previously been used to bias helical chirality in macromolecules, specifically polyacetylenes, by appending multiple chiral rotaxanes to the polymer's side chains [25].

Therefore, we report the first mechanical planar chiral rotaxanes to induce single‐handed helicity in a discrete chromophore, with the rotaxane enantiomers ***S*_mp_** or ***R*_mp_** found to induce ***P*** or ***M*** helicity in the PDI respectively (Figure 1c). These PDI‐based mechanical planar chiral rotaxanes are generated via a versatile and high‐yielding active metal templated synthesis [26], with enantiomers affording mirror image CD and CPL spectra in a range of organic solvents. Underpinning these persistent chiroptical properties, non‐covalent interactions between the macrocycle and PDI are characterised experimentally and supported by theory. Chiroptical properties also persist after prolonged heating due to the configurational stability of the chiral rotaxane architecture. The rotaxanes are monomeric in most conditions, yet we also discover that the PDI can direct self‐assembly via π–π stacking in a non‐polar solvent. Here, the mechanical bond impacts the size and geometry of the rotaxane assemblies, while the mechanical planar chirality is found to induce chirality at the supramolecular level as well.

## 2 Synthesis and Characterisation

**FIGURE 2:**
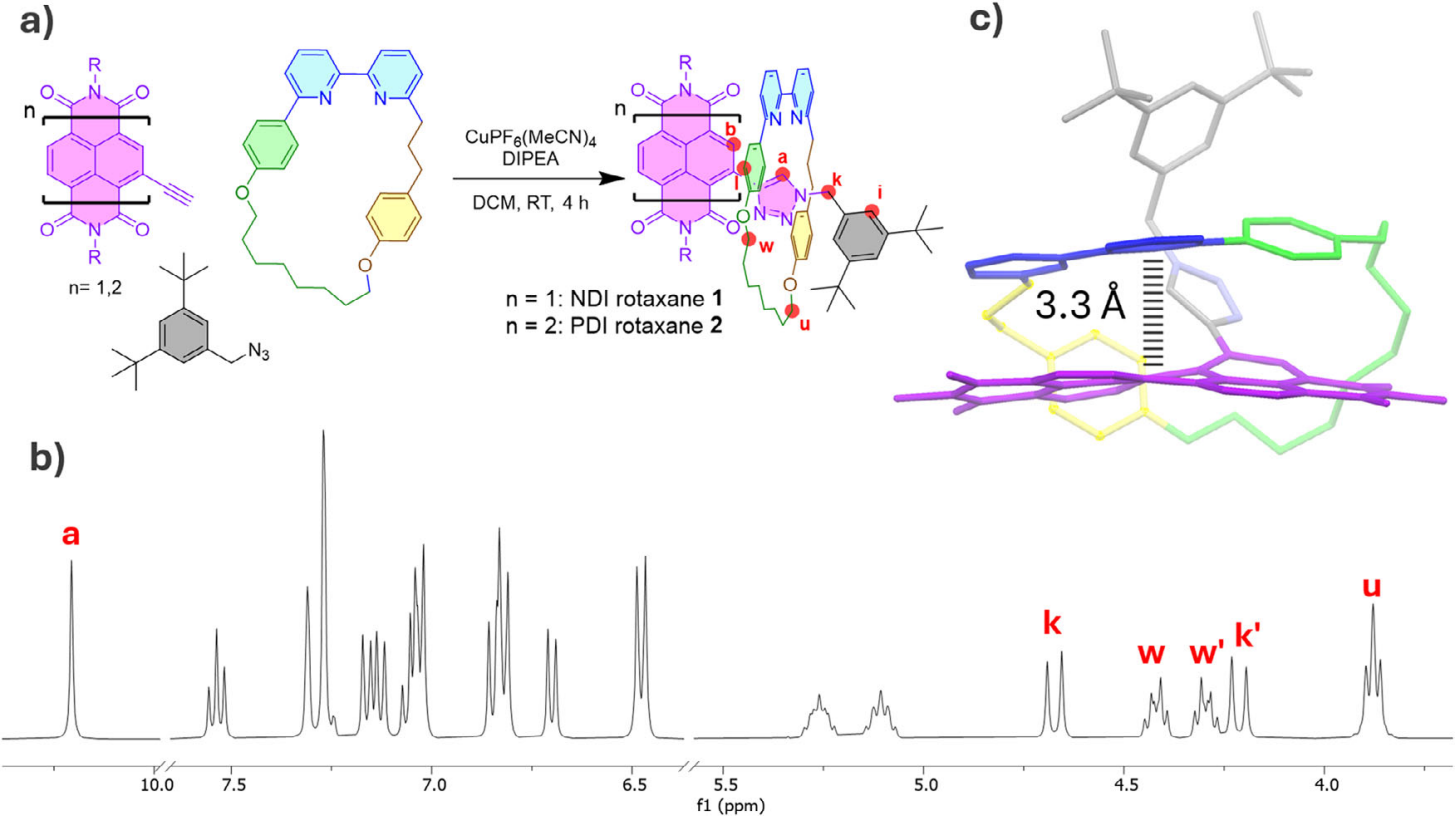
(a) General procedure for the synthesis of the NDI (**1**) and PDI (**2**) mechanically planar chiral [2]rotaxanes. (b) ^1^H NMR spectrum of **2** (1,1,2,2‐tetrachloroethane‐d_2_, 400 MHz), recorded at 373 K due to broadening at 298 K. (c) DFT structure of **2** showing a π‐stacking interaction between PDI and the alkoxybenzene–bipyridine unit.

The mechanical planar chiral PDI [2]rotaxane, **2**, was synthesised using conditions based on literature procedures (Section S1) [27, 28, 29, 30], with the mechanical bond forming reaction performed using Cu(I) catalysed active‐metal templation (Figure 2a) [26]. An analogous naphthalene diimide (NDI)‐based [2]rotaxane, **1**, was also prepared by the same route, to enable comparisons with a system containing a planar chromophore (Figure 2a). A short axle was deemed beneficial for chirality transfer by reducing the degrees of freedom of the molecular components [31]. Therefore, an aryl azide stopper was initially tested but yielded only free axle. However, exchanging this for the benzyl azide stopper gave the target rotaxanes **1** and **2** in excellent yields (up to 99%).

Relative to the free macrocycle, the ^1^H NMR spectra of rotaxane **2** showed diastereotopic splitting of several macrocycle and axle methylene groups (H_k,w,u_; Figure 2b), evidencing mechanical interlocking. This was further evidenced by ^1^H‐^1^H ROESY NMR spectra of rotaxanes **1** and **2**, which revealed multiple through‐space correlations between macrocycle and axle proton resonances confirming the close spatial proximity of the two molecular components. Moreover, for **2**, cross peaks between the macrocycle alkoxybenzene (H_l_) and the axle's triazole (H_a_) and phenyl stopper (H_i_) protons support the rotaxane co‐conformation shown in Figure 2c.

Compared to the free axle, the ^1^H NMR spectra of both rotaxanes revealed a downfield shift of triazole proton H_a_ (Δ*δ* = 2.5 ppm in **2**, Figure 2b) due to new intramolecular hydrogen bonding with the macrocycle's bipyridine unit. This change in chemical shift between the free axle and rotaxane was compared for three reporter protons in the axle component (H_a,b,i_) across a range of apolar, polar and protic solvents (Table S1 and Figures S22–S24). In all solvents, the largest Δ*δ* was consistently seen for H_a_, indicating a persistent co‐conformation in which the macrocycle is held at the triazole ‘station’ by intramolecular hydrogen bonding. This hydrogen bonding was also evident in calculated density functional theory (DFT) structures of the NDI and PDI rotaxanes **1** and **2** (Figure 2c and Section S5). These calculated structures were obtained using a combination of the CREST code [32] and the GFN2‐xTB semiempirical tight‐binding method [33, 34], with the predicted lowest energy conformers subsequently optimised by DFT (B97‐3c functional [35] and def2‐TZVP basis set [36]).

## 3 (Chir)optical Properties

**FIGURE 3:**
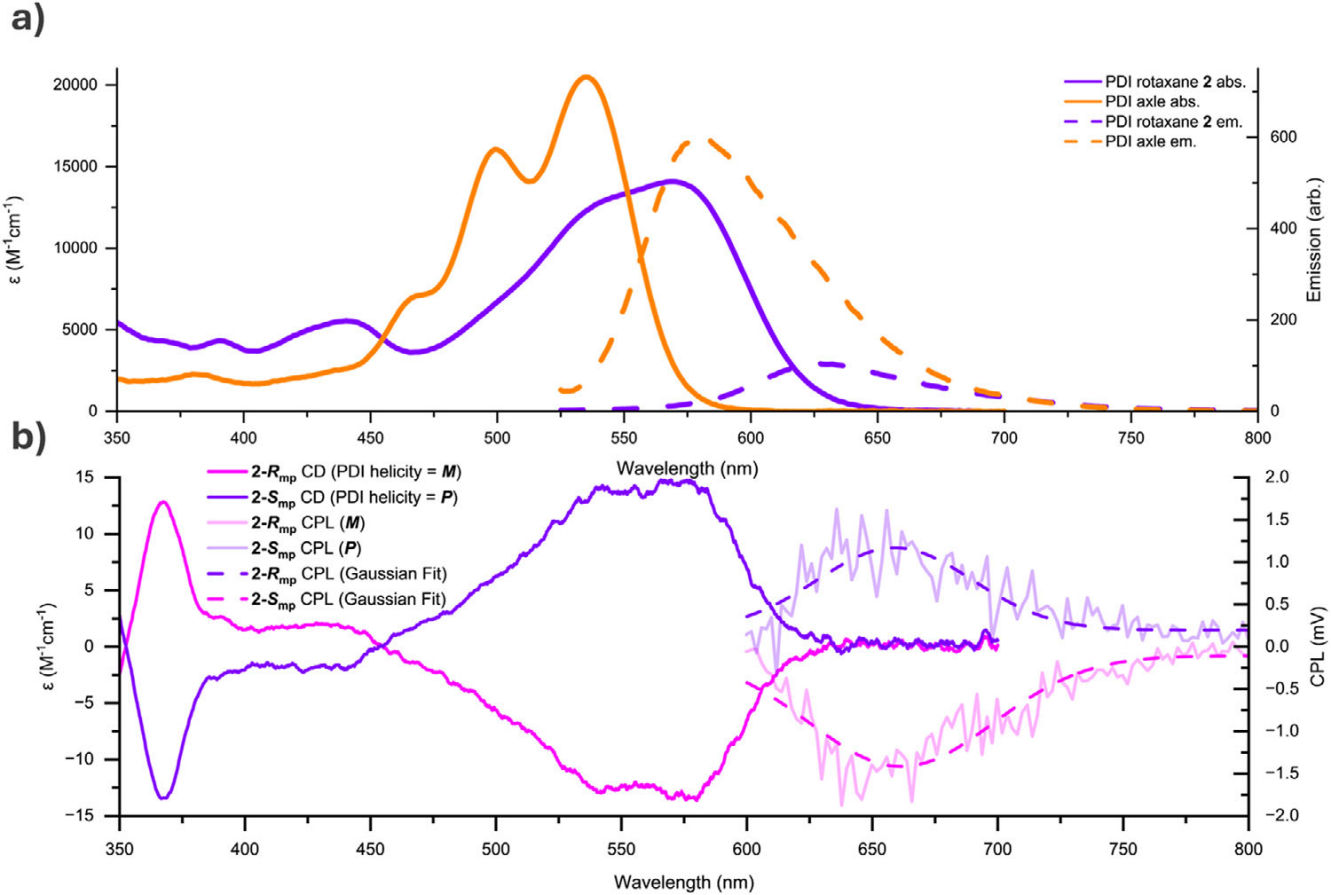
(a) UV–vis and fluorescence (*λ*_ex_ = 450 nm, 20 µM) spectra of PDI rotaxane **2** and its corresponding axle in CHCl_3_. (b) CD (CHCl_3_) and CPL spectra of the enantiomers of **2** (toluene, 100 µM, *λ*_ex_ = 475 nm).

The rotaxane DFT structures also indicated the potential for intramolecular π‐stacking (Figure 2c and Section S5). Therefore, to identify these interactions in solution, and their impact on chromophore electronics, rotaxanes **1** a nd **2** were characterised by UV–vis spectroscopy. Relative to their respective axles, both NDI and PDI chromophores showed a significant red shifting of their lowest energy absorption band (Δ*λ* = 35 nm; Figures 3a and S1). This evidences intramolecular π‐stacking interactions between the macrocycle's alkoxybenzene–bipyridine unit and the RDI, consistent with the calculated structures. Such interactions have also been reported in a PDI‐based catenane and macrocycle, with concomitant red‐shifting of absorbance spectra upon π‐stacking with aryloxy groups [37, 38]. Importantly, mixing an excess of methoxybenzene or 2,2‐bipyridine with the axle has no effect on the RDI's UV–vis spectrum (Figure S3), demonstrating that the mechanical bond plays an important role in preorganising the components to enable these π‐stacking interactions [39].

The UV–vis spectrum of NDI rotaxane **1** also exhibits an intramolecular charge transfer (ICT) band (400–500 nm, Figure S1), characteristic of π‐extended NDIs [40, 41]. This ICT band is present in the axle alone (Figure S1), confirming that the triazole heterocycle is the donor and the NDI is the acceptor, which is also in line with the predicted absorption spectrum of rotaxane **2** (Figure S30). This ICT explains the negligible fluorescence emission of **1** and its respective free axle. In contrast, PDI rotaxane **2** is emissive (Figure 2a) and, consistent with the intramolecular π‐stacking, the fluorescence is red‐shifted (Δ*λ* = 55 nm) and weaker relative to the free axle (*Φ* = 0.15 vs. 0.75).

The mechanical planar chiral enantiomers, ***S*_mp_**/***R*_mp_**, of both rotaxanes **1** and **2** were resolved by chiral high‐performance liquid chromatography (HPLC, see Section S4). As expected, there was only a single peak for the axle due to the rapid interconversion between the PDI's dynamic ***M*** and ***P*** enantiomers (Figure S25). This also explains why diastereoisomers [42] of PDI‐based rotaxane **2** were not observed by ^1^H NMR spectroscopy or HPLC.

Pleasingly, mirror image CD spectra in the visible region were measured for both enantiomers of PDI rotaxane **2**, thereby demonstrating the induction of single‐handed helicity to the twisted chromophore from the mechanical planar chiral rotaxane (Figure 3b). Furthermore, due to the PDI being emissive, the enantiomers of **2** exhibited equal and opposite CPL spectra, with the sign matching that of the lowest energy band in the CD spectrum (Figures 3b and S15, S16). We used time‐dependent DFT (ωB97x functional [43], def2‐SVP basis set [44]) to predict the CD spectra of an enantiomer of **2** and so identify the preferred PDI helicity associated with each mechanical planar chiral rotaxane enantiomer (Section S5). The rotaxane enantiomers ***S*_mp_** and ***R*_mp_** were found to induce ***P*** and ***M*** helicity in the PDI respectively. Importantly, the +/− sign of the lowest energy CD band is consistent with ***P***/***M*** helicity in related twisted PDIs [18, 45], providing further support for this assignment. For rotaxane **1**, the NDI absorptions also gave rise to opposite CD spectra for the ***S*_mp_** and ***R*_mp_** rotaxane enantiomers (Figure S2), which enabled their assignment by analogous DFT calculations (Section S5).

For both **1** and **2**, persistent chiral induction was demonstrated by CD spectroscopy, which showed no loss of the N/PDI CD signal after heating at 120°C for 24 h due to the configurational stability of the mechanical planar chiral rotaxanes (Figures S6–S9). The CD spectra of **2** were also maintained in a range of polar, non‐polar and protic solvents (Figure S13) due to the chiral rotaxane's persistent co‐conformation, in line with the ^1^H NMR spectroscopic studies (vide supra). The same was also found for CPL spectra (Figures S15 and S16), except in methanol where the emission was quenched. Together, these results demonstrate that mechanical bonding provides a robust mechanism for chiral induction to planar and twisted chromophores in mechanical planar chiral rotaxanes.

The dissymmetry factor, *g*_abs/lum_, quantifies the differential absorption/emission of circularly polarised light and is typically < 10^−2^ for discrete organic molecules [3]. In rotaxane **1**, the electronic transitions centred on the NDI (< 400 nm) [46] have *g*_abs_ = 3 × 10^−4^ (375 nm), which is of similar magnitude to a previous mechanical planar chiral rotaxane integrating a planar anthracene group [23]. In contrast, the helical PDI in rotaxane **2** exhibits a stronger chiroptical response, with *g*_abs/lum_ = 1 × 10^−3^ for the twisted chromophore's main absorption (or emission) band at 550 nm (or 635 nm). This result demonstrates the benefits of chiral induction to a non‐planar chromophore and is consistent with reports that twisted aromatics may exhibit a larger magnetic transition dipole moment, which increases rotatory strength [47]. Such ‘forbidden’ transitions [3] also include ICT bands [48, 49], and indeed amplified CD is both measured (Figure S2) and predicted (Figure S31) for the NDI rotaxane's ICT band (450 nm), where the *g*_abs_ = 2 × 10^−3^.

## 4 Chiral Self‐Assembly

**FIGURE 4:**
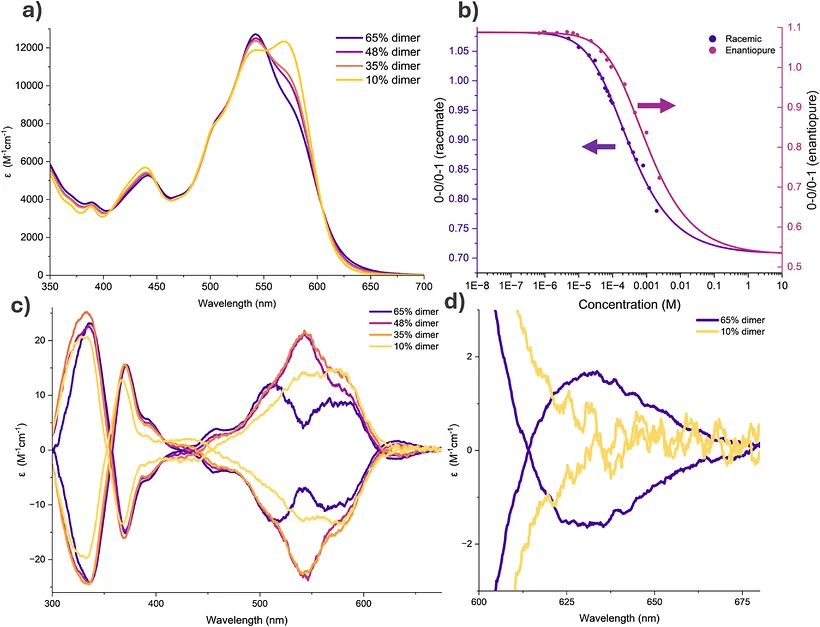
(a) UV–vis spectra of enantiopure PDI rotaxane **2**‐***R*_mp_** (*n*‐hexane, 10%–65% dimer = 0.05–2 mM). (b) Concentration‐dependent UV–vis spectra (0‐0/0‐1) of **2** racemate (1:1 ***S*_mp_**: ***R*_mp_**) and enantiopure (***R*_mp_**) with lines showing the fit to the dimer model. (c) CD spectra of ***S*_mp_**/***R*_mp_** enantiomers of **2** upon increasing dimer population (*n*‐hexane). (d) Expanded 600–675 nm region of the CD spectra in (c) to show the evolution of the Cotton effect upon PDI dimerisation.

While in most solvents rotaxane **2** is monomeric (toluene, CHCl_3_ and MeOH), PDI's are known to undergo self‐assembly in apolar solvents, driven by π–π stacking between their relatively large π‐surfaces [50]. We therefore investigated our PDI‐based rotaxane in hexanes to assess the role of mechanical bonding and mechanical planar chirality on the chromophore's (chir)optical properties at the supramolecular level. In comparison to CHCl_3_, the UV–vis spectrum of **2** in *n*‐hexane exhibits a decrease in the vibronic peak ratio (0‐0/0‐1) of the main PDI absorption band (Figure 4a), indicative of PDI self‐assembly due to (H‐type) interchromophore coupling [1]. Supporting evidence for rotaxane self‐assembly was obtained by ^1^H NMR spectroscopy, which led to a broadening of signals in cyclohexane‐d_12_ compared to those in CDCl_3_ and toluene‐d_8_. Notably, increasing the concentration of **2** (from 0.8 to 27 mM; Figure S20) results in a significant upfield shift of the PDI protons H_b‐h_ (Δ*δ* = 0.1 ppm), indicative of intermolecular π–π stacking interactions [51]. Moreover, the titration of CDCl_3_ into this sample of **2** (up to 33% *v*/*v*) produced progressive sharpening of the ^1^H NMR spectrum together with downfield shifts of both macrocycle and axle proton signals (Δ*δ* up to 0.4 ppm; Figure S21), consistent with a shift in the equilibrium back towards the rotaxane's monomeric state. The solution‐phase self‐assembly of interlocked molecules into discrete structures typically requires metal–ligand interactions [52], and indeed NDI rotaxane **1** did not aggregate in *n*‐hexane (Figure S4), likely due to its smaller π‐surface.

**FIGURE 5:**
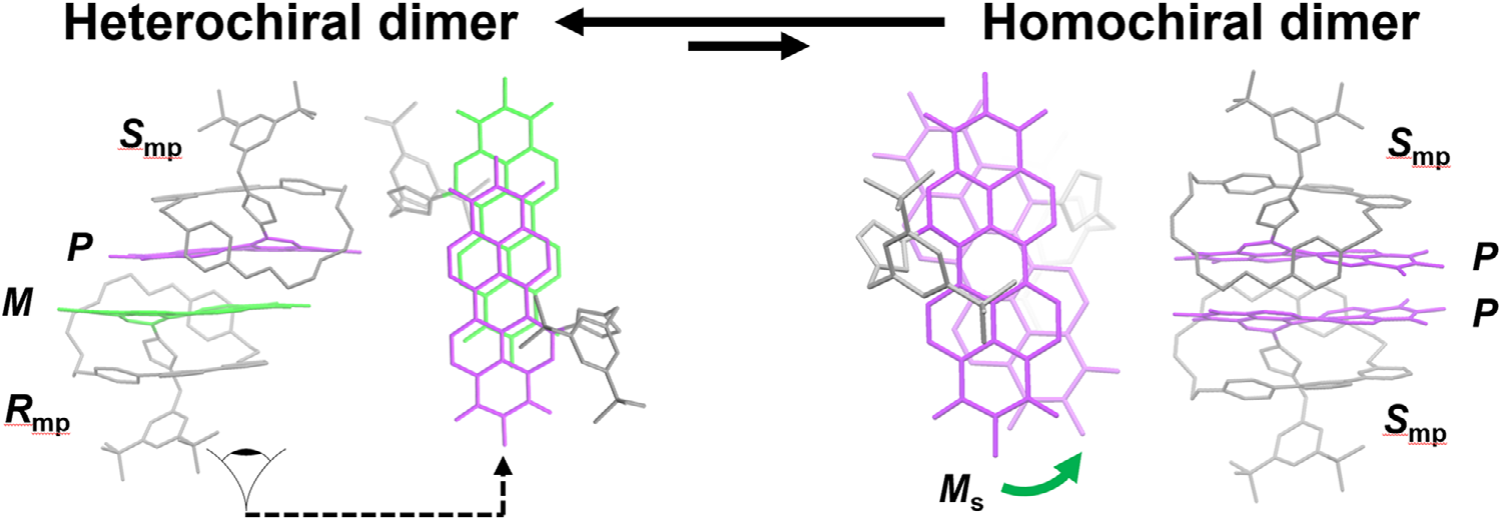
DFT structures of hetero‐ (***S*_mp–_ *R*_mp_** = ***PM***) and homochiral (***S*_mp–_ *S*_mp_** = ***PP***) dimers of PDI rotaxane **2**, and their equilibrium in the racemate as determined by UV–vis spectroscopy in *n*‐hexane. The PDI units are shaded purple/green to show ***P***/***M*** chirality. The macrocycles have been removed from the birds‐eye views of the structures for clarity.

To further characterise the unusual self‐assembly behaviour of the PDI‐based rotaxane, we measured the concentration dependence of the 0‐0/0‐1 ratio of **2** by UV–vis spectroscopy. A good fit of the binding data was achieved with the dimer model (Figures 4b and S5) [53], which is sensible given that the intramolecular PDI–bipyridine π‐stack in **2** shields one of the PDI's π‐surfaces (Figure 2c). Since PDIs often assemble into extended aggregates [54], this demonstrates that mechanical bonding can control the size of supramolecular π‐stacks. We also quantified dimer formation in distinct enantiopure (***S*_mp_** or ***R*_mp_**) and racemic (1:1 ***S*_mp_**: ***R*_mp_**) samples to isolate the importance of rotaxane chirality on self‐assembly. Interestingly, dimerisation (*K*_d_) was stronger in the racemate (*K*_d, rac_ = 3, 400 M^−1^ ± 10%) compared to the enantiopure sample (Figure 4b), meaning that heterodimerisation is favoured (*K*_d, hetero_ = 11, 600 M^−1^ ± 14%) over homodimerisation (*K*_d, homo_ = 950 M^−1^ ± 16%) and so the racemate is composed of 86% heterodimers (Figure 5) [55]. This highlights the importance of enantiopurity in chiral materials [56], due to the potential for contamination from the opposite enantiomer to alter the self‐assembly outcome.

Heterochirality is unusual in π‐stacks between non‐planar aromatics [38, 57] with chiral complementarity typically favouring a homochiral, and co‐facial, geometry [58, 59, 60]. However, we propose that the macrocycle of **2**, which is located at one of the PDI's naphthalene subunits, restricts co‐facial π–π stacking, instead directing PDI self‐assembly via the unhindered naphthalene subunit to generate a slipped‐stack dimer (Figure 5), a favoured geometry in heterochiral π‐stacks of twisted PDIs [15, 61, 62]. Indeed, this geometry is consistent with the larger 0‐0/0‐1 ratio of the (predominantly) heterochiral PDI rotaxane dimer in comparison to the homochiral dimer, 0.71 versus 0.53 (Figures 4b and S10, S11), since the long‐axis displacement of PDIs results in weaker interchromophore coupling [63]. The slipped‐stack geometry was also confirmed by calculations, with predicted UV–vis spectra showing weaker H‐type coupling (greater Davydov splitting) in the heterochiral dimer relative to the homochiral structure (Section S5).

Analogous concentration dependent CD spectra were recorded with enantiopure solutions of PDI‐based rotaxane **2** in *n*‐hexane (***S*_mp_** and ***R*_mp_**). The CD of the main PDI absorption band was enhanced upon rotaxane self‐assembly, giving a *g*_abs_ = 2 × 10^−3^ at a dimer population of 48% (Figure 4c). The CPL spectra of **2** were largely unchanged at low and high concentrations (*g*_lum_ = 1 × 10^−3^; Figures S15 and S17), consistent with the negligible changes in rotaxane fluorescence observed upon dimerisation (Figure S14). These findings suggest that emission originates from the PDI's locally excited state and that excimer formation may be disfavoured by the sterically encumbering rotaxane architecture.

At larger dimer populations (65%), a Cotton effect is observed at ∼625 nm in the CD spectrum (Figure 4d), characteristic of supramolecular chirality in the PDI dimer, that is, due to rotationally displaced chromophores. Given the H‐type interchromophore coupling (Figure 4a), the +/− sign of the Cotton effect indicates right/left‐handed supramolecular chirality in the dimer [64], which we label as ***P*_s_**/***M*_s_** [65]. Therefore, there is an additional element of chiral induction upon the self‐assembly of PDI rotaxane **2**; with ***S*_mp_**/***R*_mp_** mechanical planar chirality inducing ***P***/***M*** helicity in the molecular PDI *and* ***M*_s_**/***P*_s_** supramolecular chirality in the homochiral dimers [66]. This preference for single‐handed dimers was confirmed by calculations on rotaxane **2**‐***S*_mp_**. At this level of theory (Section S5), the homochiral PDI dimer (i.e., ***S*_mp_–*S*_mp_** = ***PP***) with ***M*_s_** supramolecular chirality was found to be lower in energy than an analogous structure with ***P*_s_** chirality, and has a predicted CD spectrum that is more consistent with experiment.

## 5 Summary and Conclusions

In conclusion, we have used mechanical planar chirality to induce single‐handed helicity in a discrete chromophore, which affords persistent chiroptical properties including, for the first time, CPL from a mechanical planar chiral rotaxane. While this strategy is also compatible with a planar NDI (**1**), the stronger chiroptical response of the PDI‐based rotaxane **2** demonstrates the added value of chiral induction to twisted chromophores. The rotaxanes are prepared by active metal templation and resolved by HPLC, with their various chiral elements assigned by a combination of experiment and theory. By design, the rotaxanes exhibit intramolecular hydrogen bonding and π–π interactions which, alongside their chiral configurational stability, ensure persistent CD and CPL, representing a distinct approach to (non‐)covalently engineered chiral PDIs (Figure 1). Unusually for interlocked molecules, rotaxane **2** undergoes self‐assembly, with mechanical bonding found to impact the size (dimer), geometry (slipped‐stack) and chirality (heterochiral) of the π‐stacked structure. Moreover, in addition to the induction of molecular chirality, mechanical planar chirality is also shown to induce single‐handedness at the supramolecular level in the homochiral PDI dimers.

Overall, this work broadens our understanding of chiral induction in mechanically interlocked molecules and, underpinned by versatile, high‐yielding templated syntheses, establishes a strategy for controlling the chiroptical and supramolecular properties of twisted chromophores more generally.

## Author Contributions

**Thomas E. Lawson**: investigation, writing – original draft. **Denis Hartmann**: investigation, writing – review and editing. **Artemijs Krimovs**: investigation, writing – review and editing. **Qilong Sun**: investigation. **Robert Pal**: investigation, supervision, funding acquisition, writing – review and editing. **Timothy A. Barendt**: conceptualisation, funding acquisition, supervision, writing – review and editing.

## Conflicts of Interest

The authors declare no conflicts of interest.

## Supporting information




**Supporting File 1**: All synthetic method descriptions, product characterisation data, spectroscopic data and other supplementary Schemes S1 and S2, Figures (S1–S39) and Tables (S1–S6), including NMR spectra, UV–vis spectra, CD spectra and chiral HPLC chromatograms, so on, and computational data can be found in the Supporting Information. The relevant computational structures are available in the folder named “DFT structures”. The authors have cited additional references within the Supporting Information [67, 68, 69, 70, 71].

**Supporting File 2**: anie73891‐sup‐0002‐SuppMat.zip.

## Data Availability

The data that supports the findings of this study are available in the Supporting Information of this article.
