# Supplementary material for: A Mechanical Planar Chiral Rotaxane Induces Single‐Handed Helicity in a Twisted Perylene Diimide
Source: Angew Chem Int Ed Engl. 2026 Jul 31;65(39):e1877364. doi: 10.1002/anie.1877364 (PMC13592320; doi:10.1002/anie.1877364)
Supplement: Supplementary file 1 [file ANIE-65-e1877364-s001.pdf]

Supporting Information

A Mechanical Planar Chiral Rotaxane Induces Single-Handed Helicity in a Twisted Perylene Diimide

Thomas E. Lawson,[1] Denis Hartmann,[1] Artemijs Krimovs,[2] Qilong Sun,[1] Robert Pal,[2] Timothy A. Barendt[1,*]

[1]School of Chemistry, University of Birmingham, Edgbaston, Birmingham, B15 2TT, United Kingdom

[2]University of Durham, Department of Chemistry, South Road, Durham, DH1 3LE, United Kingdom

Contents:

1) Synthesis 2
2) Photophysics 10
3) Further ^{1}H NMR Spectroscopy 18
4) Chiral HPLC 23
5) Computational Chemistry 25
6) NMR Spectra 34
7) Mass Spectra 46
8) References 52

1) Synthesis

General Synthesis

Reagents were purchased from commercial sources (Merck, Acros Organics, Fluorochem and Alfa Aesar) and used without further purification. Solvents were used as supplied (analytical/HPLC-grade from Fisher or Sigma-Aldrich). If required, solvents were dried over molecular sieves (3 Å) overnight before use and stored under N_2 atmosphere. Petroleum ether (PE) over a boiling point range of 40–60 °C was used. Eluent mixtures are reported in volume:volume or %vol. Column chromatography was carried out using Merck Silica Gel 60 Å, 230-400 mesh, 40-63 µm particle size. Preparative TLC was performed using 20 × 20 cm plates with 1 cm silica thickness. NMR spectroscopy measurements were recorded using a Bruker AVIII 300, AVIII 400, AV Neo 400 or AV Neo 500 instrument and peaks were referenced to the residual solvent peak. MALDI-MS measurements were carried out on a Bruker ultrafleXtreme MALDI-TOF. Electrospray Ionisation Mass Spectrometry (ESI-MS) measurements were carried out on a Waters Xevo G2-XS TOF Mass Spectrometer.

Compounds **b-d**,[1] **e**,[2] **f**,[3] **h**,[4] were synthesised via literature procedures.

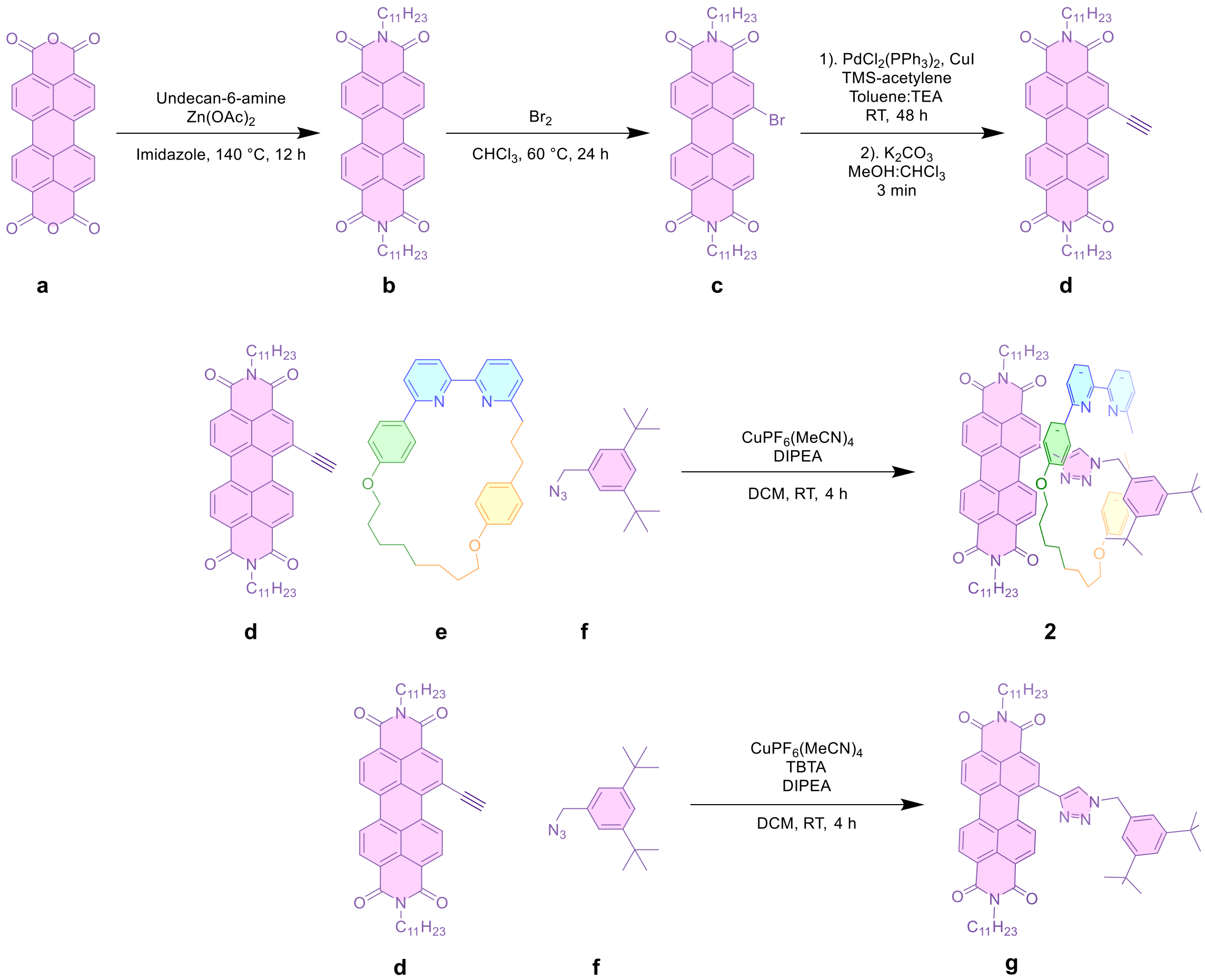

Scheme S1: Multistep synthesis of PDI [2]rotaxane (**2**) and free axle (**g**)

PDI [2]Rotaxane (2)

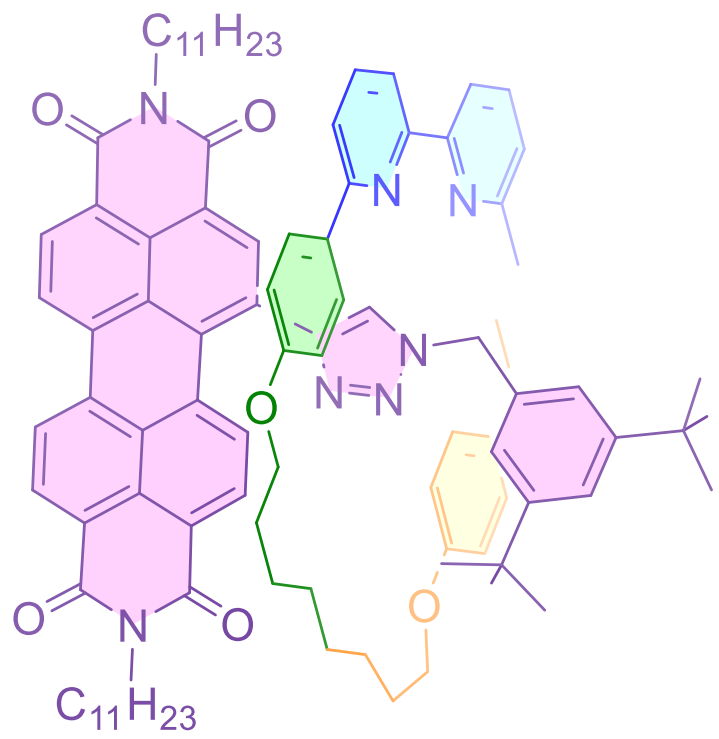

To a round-bottom flask under nitrogen atmosphere was added **d** (45.46 mg, 0.062 mmol, 1 equiv.), **e** (34.08 mg, 0.069 mmol, 1.1 equiv), $CuPF_6(MeCN)_4$ (21.1 mg, 0.056 mmol, 0.9 equiv), **f** (18.5 mg, 0.075 mmol, 1.2 equiv.) in DCM (5 mL). DIPEA (8.13 mg, 0.063 mmol, 1 equiv.) was added and the reaction stirred at room temperature for 4 h. KCN (40 mg, 0.62 mmol) in MeCN (2 mL) was added and stirred for a further 2 hours. Solvent was removed by rotary evaporation and the crude material was washed with DCM:water. To obtain the pure product preparative thin-layer-chromatography (prep TLC, DCM:hexane) was yielded the desired product as a purple solid (59.8 mg, 0.04 mmol, 65 % yield).

^{1}H NMR: (400 MHz, 1,1,2,2-tetrachloroethane-*d2*, 373 K) δ 10.21 (s, 1H), 8.64 (d, J = 8.1 Hz, 1H), 8.61 (s, 1H), 8.52 (d, J = 7.9 Hz, 1H), 8.44 – 8.36 (m, 3H), 8.24 (d, J = 8.1 Hz, 1H), 7.54 (t, J = 7.8 Hz, 1H), 7.31 (d, J = 1.8 Hz, 1H), 7.27 (d, J = 1.8 Hz, 2H), 7.16 (d, J = 7.8 Hz, 1H), 7.13 (d, J = 7.8 Hz, 1H), 7.04 (t, J = 3.8 Hz, 2H), 7.02 (s, 1H), 6.85 (d, J = 8.1 Hz, 1H), 6.82 (d, J = 8.5 Hz, 3H), 6.70 (d, J = 7.8 Hz, 1H), 6.48 (d, J = 8.4 Hz, 2H), 6.00 (s, 1H), 5.76 (d, J = 8.4 Hz, 2H), 5.27 (td, J = 9.4, 4.7 Hz, 1H), 5.15 – 5.06 (m, 1H), 4.67 (d, J = 14.4 Hz, 1H), 4.42 (dt, J = 9.5, 6.5 Hz, 1H), 4.30 (dt, J = 9.5, 6.5 Hz, 1H), 4.21 (d, J = 14.4 Hz, 1H), 3.88 (t, J = 7.2 Hz, 2H), 2.93 – 2.70 (m, 4H), 2.36 (s, 2H), 2.29 – 1.65 (m, 20H), 1.42 – 1.27 (m, 29H), 1.09 (s, 18H), 0.98 – 0.86 (m, 14H).

See full assignment of ^{1}H NMR spectrum in section 5

^{13}C NMR: (400 MHz, 1,1,2,2-tetrachloroethane-*d2*) δ 136.56, 136.48, 135.93, 134.29, 134.09, 132.95, 131.68, 131.28, 130.69, 130.54, 130.06, 129.63, 129.54, 128.90, 128.20, 128.07, 127.84, 127.66, 126.76, 125.96, 124.37, 123.24, 122.67, 122.11, 121.59, 121.33, 119.90, 119.28, 117.81, 115.38, 114.80, 69.33, 67.92, 54.79, 54.65, 54.03, 38.31, 37.88, 35.37, 34.71, 34.08, 32.68, 32.64, 31.93, 31.89, 31.25, 29.95, 29.80, 29.71, 28.89, 26.81, 26.79, 26.48, 26.37, 22.67, 22.58, 22.54, 14.04, 13.99.

ESI-MS: found 1458.8673, $[M-H]^-$ requires 1458.8680

MonoTriazole-C11-PDI (g)

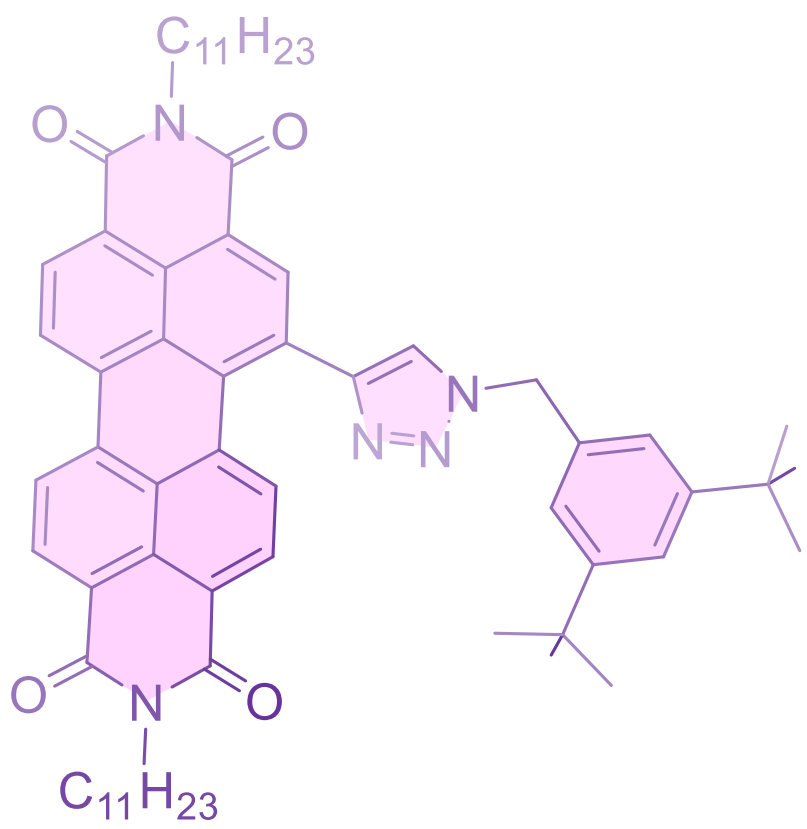

To a round-bottom flask under nitrogen atmosphere was added **d** (22.73 mg, 0.031 mmol, 1 equiv), TBTA (3.34 mg, 0.006 mmol,0.2 equiv), $CuPF_6(MeCN)_4$ (2.34 mg, 0.006 mmol, 0.2 equiv), **f** (9.26 mg, 0.037 mmol, 1.2 equiv) in DCM (5 mL). DIPEA (4.1 mg, 0.031 mmol) was added and the reaction stirred at room temperature for 4 h. Solvent was removed by rotary evaporation and the crude material was washed with DCM:water. To obtain the pure product preparative thin-layer-chromatography (prep TLC, DCM:hexane) was yielded the desired product as a purple solid (21.5 mg, 0.022 mmol, 71 % yield).

^{1}H NMR: (400 MHz, Chloroform-*d*) δ 8.72 – 8.50 (m, 5H), 8.19 (s, 1H), 7.95 (d, 1H), 7.69 (s, 1H), 7.48 (t, *J* = 1.8 Hz, 1H), 7.24 (d, *J* = 1.8 Hz, 2H), 5.67 (s, 2H), 5.21 – 5.09 (m, 3H), 2.24 (s, 4H), 1.85 – 1.78 (m, 4H), 1.34 (s, 18H), 1.30 – 1.23 (m, 24H), 0.82 (m, 12H).

See full assignment of ^{1}H NMR spectrum in section 5

^{13}C NMR: (400 MHz, Chloroform-*d*) δ 164.68, 164.39, 163.64, 163.14, 152.16, 148.52, 136.40, 135.69, 134.92, 134.29, 134.15, 133.92, 133.31, 131.75, 131.60, 131.00, 130.88, 130.52, 129.80, 129.42, 129.17, 128.95, 128.00, 127.45, 123.61, 123.28, 122.90, 122.61, 121.47, 55.41, 54.74, 54.65, 34.97, 32.32, 32.27, 31.77, 31.71, 31.43, 29.71, 26.60, 26.55, 22.57, 22.55, 14.06, 14.03

ESI-MS: found 966.5869, $[M-H]^-$ requires 966.5902

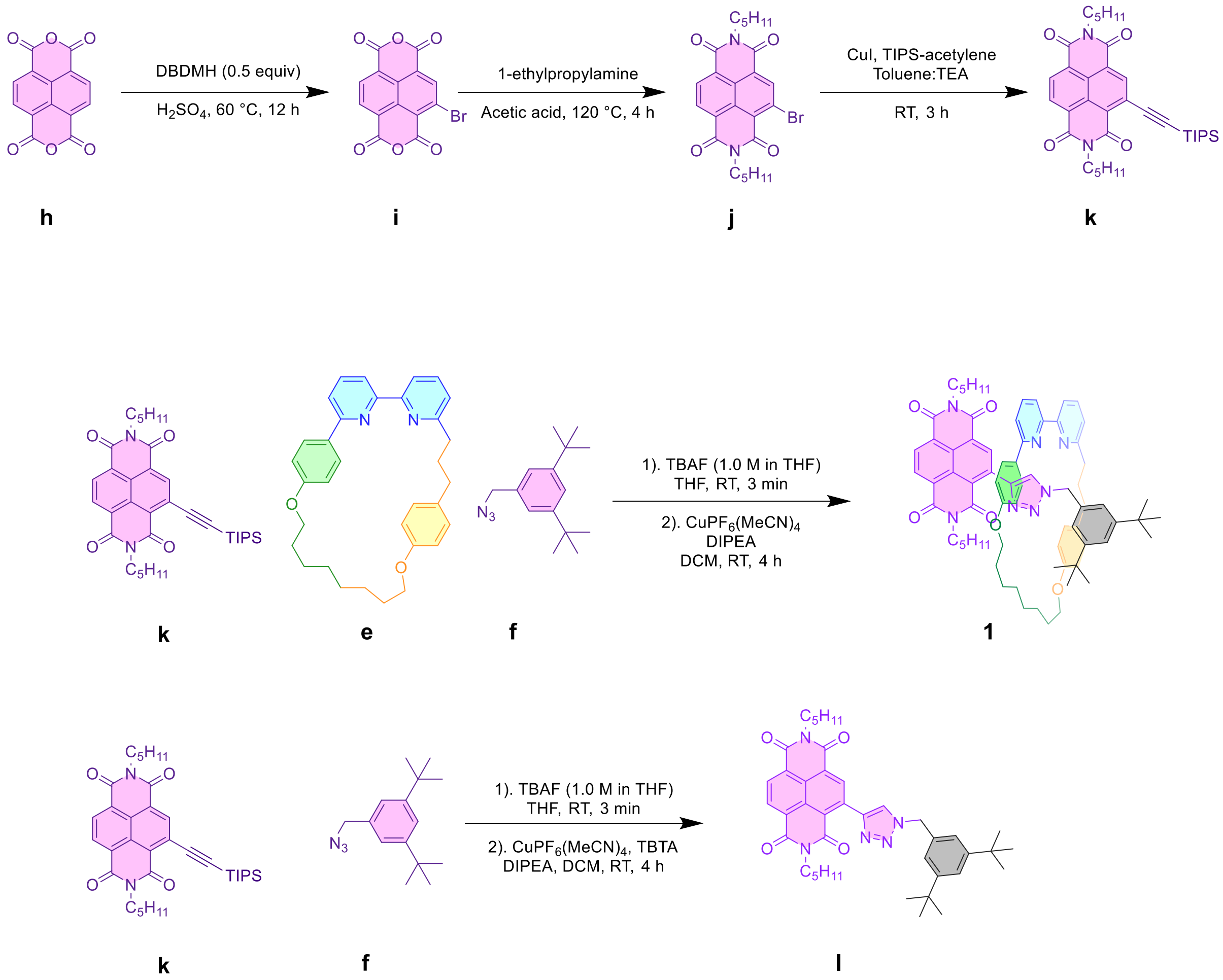

Scheme S2: Multistep synthesis of NDI [2]rotaxane (**1**) and free axle (**l**)

MonoBr-C5-NDI (j)

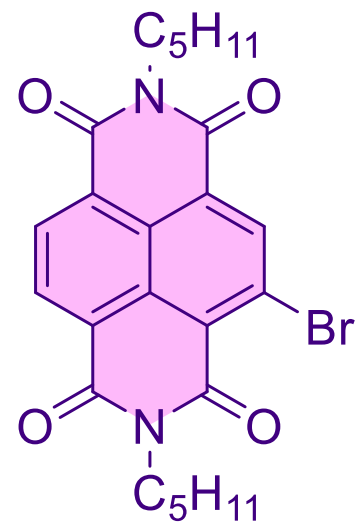

Compound **h** (2.5 g, 9.32 mmol, 1 equiv), was dissolved in concentrated sulphuric acid (100 mL) and stirred to give a homogenous cream solution. 1,3-dibromo-5,5-dimethylhydantoin (1.49 g, 5.22 mmol, 0.56 equiv.) was added and the reaction stirred at 60 °C overnight. The reaction was then poured into and ice/water mixture and the resulting yellow precipitate was collected via filtration and dried in an oven to remove water. This gives a mixture of **i** as well as a mixture of NDAs with different levels of bromination. This crude material is then redissolved in acetic acid (200 mL) and 1-ethylpropylamine (6.5 mL, 52.2 mmol, 5.6 equiv.) was added. The reaction is then stirred at 120 °C for 4 hours, then poured into ice/water and the precipitate collected via filtration. The precipitate is washed with excess water and hexane to remove excess amine and acid. Compound **j** is then able to be isolated via silica column chromatography (DCM:hexane), this yielded the desired product as an off-white solid (170 mg, 0.35 mmol, 3.76 % yield across two steps).

^{1}H NMR: (400 MHz, Chloroform-*d*) δ 8.90 (s, 1H), 8.82 – 8.69 (m, 2H), 5.01 (m, 2H), 2.19 m, 4H), 2.00 – 1.82 (m, 4H), 0.87 (m, 12H)

See full assignment of ^{1}H NMR spectrum in section 5

^{13}C NMR: (400 MHz, Chloroform-*d*) δ 163.22, 162.67, 162.22, 161.42, 138.55, 131.69, 131.48, 130.75, 128.67, 128.47, 126.81, 126.00, 125.83, 123.97, 58.58, 58.32, 24.89, 24.86, 11.30, 11.21

MS: MS data not available due to poor ionisation by both ESI and MALDI methods.

Mono-TIPSAlkynyl-C5-NDI (k)

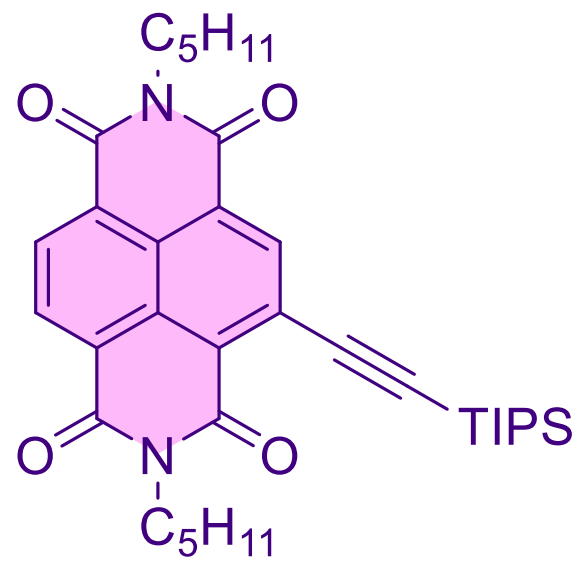

TIPS-acetylene (39 uL, 0.175 mmol, 1 equiv.) and CuI (33.4 mg, 0.175 mmol, 1 equiv.) are stirred in DMF:TEA (1:1, 10 mL) at 90 °C for 30 min, after which **j** (85 mg, 0.175 mmol, 1 equiv) is added and the reaction is stirred for a further 30 min. After cooling the solvent was removed by rotary evaporation and the product was isolated via silica column chromatography to give **k** as a pale yellow solid (101.1 mg, 172.3 mmol, 98.4 % yield).

^{1}H NMR: (400 MHz, Chloroform-*d*) δ 8.75 – 8.63 (m, 3H), 4.99 (m, 1H), 2.19 (m, 4H), 1.88 (m, 4H), 1.28 – 1.11 (m, 21H), 0.86 (m, 12H)

See full assignment of ^{1}H NMR spectrum in section 5

^{13}C NMR: (400 MHz, Chloroform-*d*) δ 163.21, 161.62, 136.58, 131.19, 130.76, 129.48, 128.80, 127.82, 127.18, 126.95, 126.53, 126.26, 125.74, 125.26, 107.32, 105.15, 58.18, 58.04, 24.95, 18.66, 11.38

MALDI-MS: found 586.032, [$M^{\cdot -}$] requires 586.320

MPC-NDI[2]Rotaxane (1)

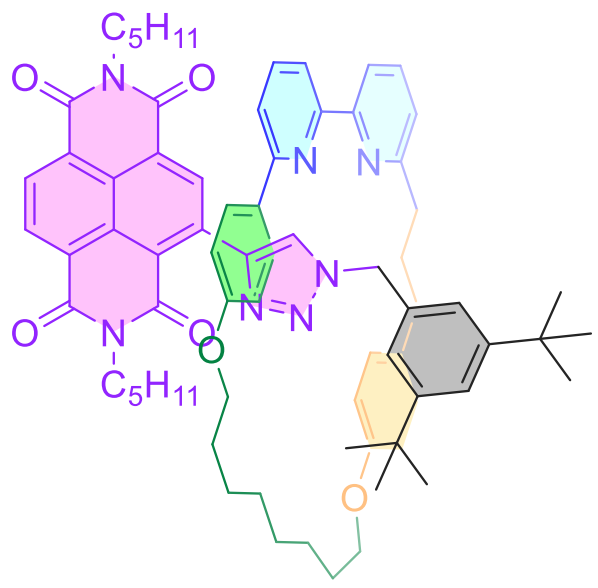

To a round-bottomed flask containing **k** (77 mg, 0.131 mmol, 1 equiv) in THF (10 mL), TBAF (1.0 M in THF, 131 uL, 0.131 mmol,1 equiv.) was added and stirred for 3 minutes. Deprotection was confirmed by MALDI-MS and the reaction was washed with 1 M HCl then extracted with DCM and purified by flash column chromatography (DCM:Hexane) to give the deprotected **k** as a yellow solid (21.7 mg, 0.05 mmol, 38 % yield).

To a round-bottom flask under nitrogen atmosphere was added (21.7 mg, 0.05 mmol, 1 equiv.) of deprtoected material, **5** (24.8 mg, 0.05 mmol, 1 equiv), $CuPF_6(MeCN)_4$ (16.9 mg, 0.045 mmol, 0.9 equiv), **6** (24.7 mg, 0.1 mmol, 2 equiv.) in DCM (5 mL). DIPEA (6.5 mg, 0.05 mmol, 1 equiv) was added and the reaction stirred at room temperature for 4 h. KCN (6.5 mg, 0.1 mmol) in MeCN (2 mL) was added and stirred for a further 2 hours. Solvent was removed by rotary evaporation and the crude material was washed with DCM:water. To obtain the pure product preparative thin-layer-chromatography (prep TLC, DCM:hexane) was yielded the desired product as a yellow solid (58.8 mg, 0.05 mmol, 99 % yield).

^{1}H NMR: (400 MHz, 1,1,2,2-tetrachloroethane-*d2*, 373 K) δ 10.06 (s, 1H), 8.82 (s, 1H), 8.71 – 8.62 (m, 2H), 7.81 (t, *J* = 7.7 Hz, 1H), 7.54 (d, *J* = 7.7 Hz, 1H), 7.43 – 7.35 (m, 4H), 7.32 (d, *J* = 7.6 Hz, 1H), 7.24 (t, *J* = 7.8 Hz, 1H), 6.98 (d, *J* = 8.3 Hz, 2H), 6.84 (dd, *J* = 8.3, 6.3 Hz, 3H), 6.56 – 6.49 (m, 2H), 6.11 – 6.03 (m, 2H), 5.14 (tt, *J* = 9.3, 5.9 Hz, 1H), 4.78 – 4.66 (m, 1H), 4.70 (s, 2H), 4.31 (dt, *J* = 9.1, 7.0 Hz, 1H), 4.11 – 3.97 (m, 3H), 3.18 (td, *J* = 13.3, 3.9 Hz, 1H), 3.03 – 2.92 (m, 1H), 2.96 – 2.87 (m, 1H), 2.71 – 2.56 (m, 2H), 2.36 (ddq, *J* = 14.8, 9.4, 7.5 Hz, 2H), 2.14 – 1.97 (m, 5H), 1.85 (s, 4H), 1.97 – 1.65 (m, 4H), 1.20 (d, *J* = 0.9 Hz, 17H), 1.05 (td, *J* = 7.4, 2.1 Hz, 6H), 0.66 (dt, *J* = 20.4, 7.4 Hz, 6H).

See full assignment of ^{1}H NMR spectrum in section 5

^{13}C NMR: (400 MHz, 1,1,2,2-tetrachloroethane-*d2*) δ 164.06, 163.35, 163.23, 163.19, 162.63, 161.80, 159.62, 157.99, 157.30, 152.19, 141.88, 137.86, 137.26, 136.17, 134.28, 132.51, 131.44, 130.15, 129.94, 129.62, 129.21, 128.34, 127.29, 126.15, 125.65, 124.74, 124.68, 123.03, 122.49, 122.26, 120.61, 120.01, 119.94, 118.31, 115.28, 114.91, 69.29, 67.96, 58.16, 57.32, 54.54, 38.21, 35.11, 34.74, 31.36, 29.90, 29.71, 29.51, 29.32, 28.94, 26.32, 26.15, 25.27, 25.15, 24.57, 11.37, 11.27.

ESI-MS:found 1168.6600, $[M+H]^+$ requires 1168.6634

MonoTriazole-C5-NDI (l)

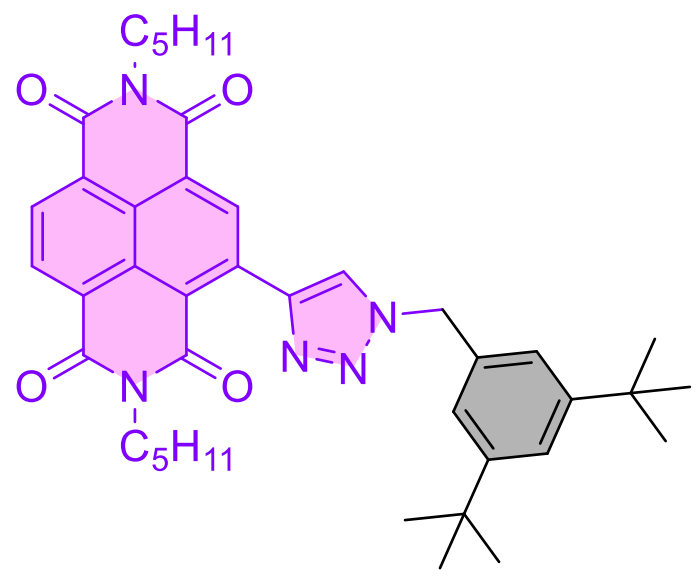

To a round-bottomed flask containing **k** (60 mg, 0.102 mmol, 1 equiv) in THF (10 mL), TBAF (1.0 M in THF, 102 uL, 0.102 mmol, 1 equiv.) was added and stirred for 3 minutes. Deprotection was confirmed by MALDI-MS and the reaction was washed with 1 M HCl then extracted with DCM and purified by flash column chromatography (DCM:Hexane) to give deprotected **k** as a yellow solid (17 mg, 0.04 mmol, 39 % yield).

To a round-bottom flask under nitrogen atmosphere was added (17 mg, 0.04 mmol, 1 equiv) of deprotected material, TBTA (4.2 mg, 0.008 mmol, 0.2 equiv), $CuPF_6(MeCN)_4$ (2.9 mg, 0.008 mmol, 0.2 equiv), **6** (19.4 mg, 0.08 mmol, 2 equiv.) in DCM (5 mL). DIPEA (5.1 mg, 0.04 mmol, 1 equiv.) was added and the reaction stirred at room temperature for 4 h. Solvent was removed by rotary evaporation and the crude material was washed with DCM:water. To obtain the pure product preparative thin-layer-chromatography (prep TLC, DCM:hexane) was yielded the desired product as a yelllow solid (26.2 mg, 0.04 mmol, 98 % yield).

^{1}H NMR: (400 MHz, Chloroform-*d*) δ 9.34 (s, 1H), 8.76 (d, *J* = 7.7 Hz, 1H), 8.70 (d, *J* = 7.6 Hz, 1H), 8.34 (s, 1H), 7.45 (t, *J* = 1.8 Hz, 1H), 7.24 (d, *J* = 1.8 Hz, 2H), 5.67 (s, 2H), 5.09 – 4.89 (m, 2H), 2.30 – 2.06 (m, 4H), 1.99 – 1.81 (m, 4H), 1.33 (s, 18H), 0.87 (m, 6H).

See full assignment of ^{1}H NMR spectrum in section 5

^{13}C NMR: NMR (400 MHz, Chloroform-*d*) δ 163.71, 163.58, 163.43, 163.30, 163.12, 151.93, 144.22, 136.51, 135.30, 133.58, 131.23, 129.90, 127.88, 126.84, 126.69, 126.27, 125.82, 122.85, 122.41, 121.55, 58.18, 58.10, 54.91, 34.95, 31.42, 24.92, 24.87, 11.31, 11.23.

ESI-MS: found 676.3890, $[M+H]^+$ requires 676.3857

2) Photophysics

General

UV-visible absorbance spectra were recorded on a Shimadzu UV-3600i Plus Spectrometer with a wavelength accuracy of ± 0.2 nm in the visible and UV region, a baseline flatness of ± 0.002 nm (200 – 3000 nm) and a noise level of <0.00008 Abs (900 nm).

Absolute fluorescence quantum yields were obtained on an Edinburgh Instruments FLS1000 photoluminescence spectrometer fitted with an integrating sphere. All samples were recorded in chloroform at 20 μM with a 7 - 8 nm excitation slit and 0.1 - 0.2 nm emission slit width. Experiments were carried out in solution using 1 cm path length quartz cuvettes with four transparent polished faces.

Circular dichroism (CD) measurements were taken on a JASCO J-1500 CD Spectrophotometer with a wavelength accuracy ± 0.2 nm (250 to 500 nm), ± 0.5 nm (500 to 800 nm), a photometric accuracy of ± 0.01 Abs and a CD root mean square noise < 0.007 mdeg (500 nm).

CPL was measured with a home-built (modular) spectrometer. The excitation source was a broad band (200 – 1000 nm) laser- driven light source EQ 99 (Elliot Scientific). The excitation wavelength was selected by feeding the broadband light into an Acton SP-2155 monochromator (Princeton Instruments, 7 μm slit); the collimated light was focused into the sample cell (1 cm quartz cuvette, temperature controlled at 20 ºC). Sample PL emission was collected perpendicular to the excitation direction with a lens (f = 150 mm). The emission was fed through a photoelastic modulator (PEM) (Hinds Series II/FS42AA) and through a linear sheet polariser (Comar). The light was then focused into a second scanning monochromator (Acton SP-2155, 7 μm slit) and subsequently on to a photomultiplier tube (PMT) (Hamamatsu H10723 series). The detection of the CPL signal was achieved using the field modulation lock-in technique. The electronic signal from the PMT was fed into a lock-in amplifier (Hinds Instruments Signaloc Model 2100). The reference signal for the lock-in detection was provided by the PEM control unit. The monochromators, PEM control unit and lock-in amplifier were interfaced to a desktop PC and controlled by a custom-written Labview graphic user interface. The lock-in amplifier provided two signals, an AC signal corresponding to (IL- IR) and a DC signal corresponding to (IL + IR) after background subtraction. The emission dissymmetry factor was therefore readily obtained from the experimental data, as 2 AC/DC. Errors of g_{lum} are ±5 x 10-5. The emission spectra were recorded with 2 nm step size and the slits of the detection monochromator were set to a slit width corresponding to a spectral resolution of 3.5 nm. CPL spectra (as well as total emission spectra) were obtained through an averaging procedure of several scans.

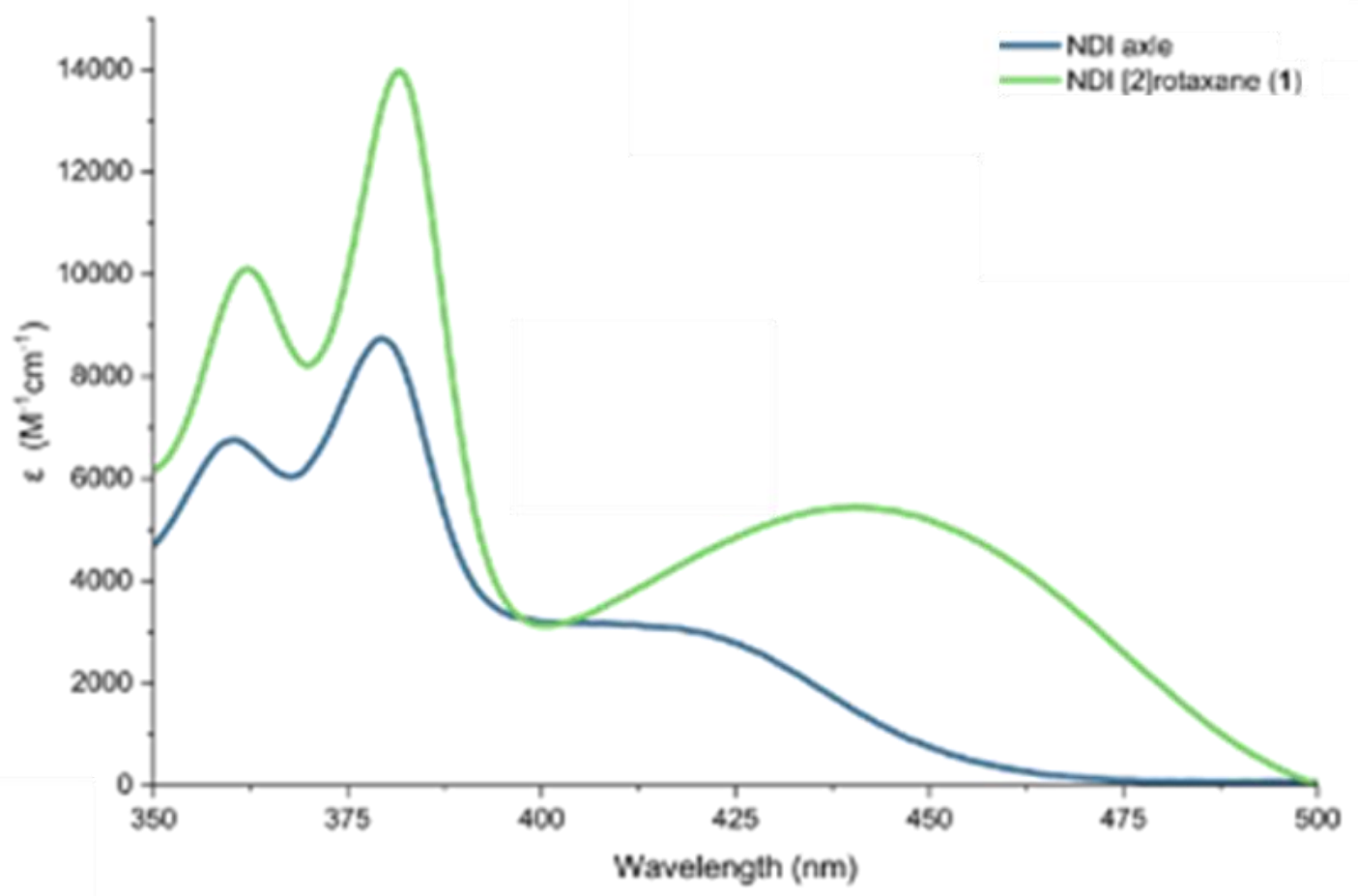

Figure S1: UV-vis spectra of NDI rotaxane **1** and its free axle (**I**) in chloroform at 15 μM

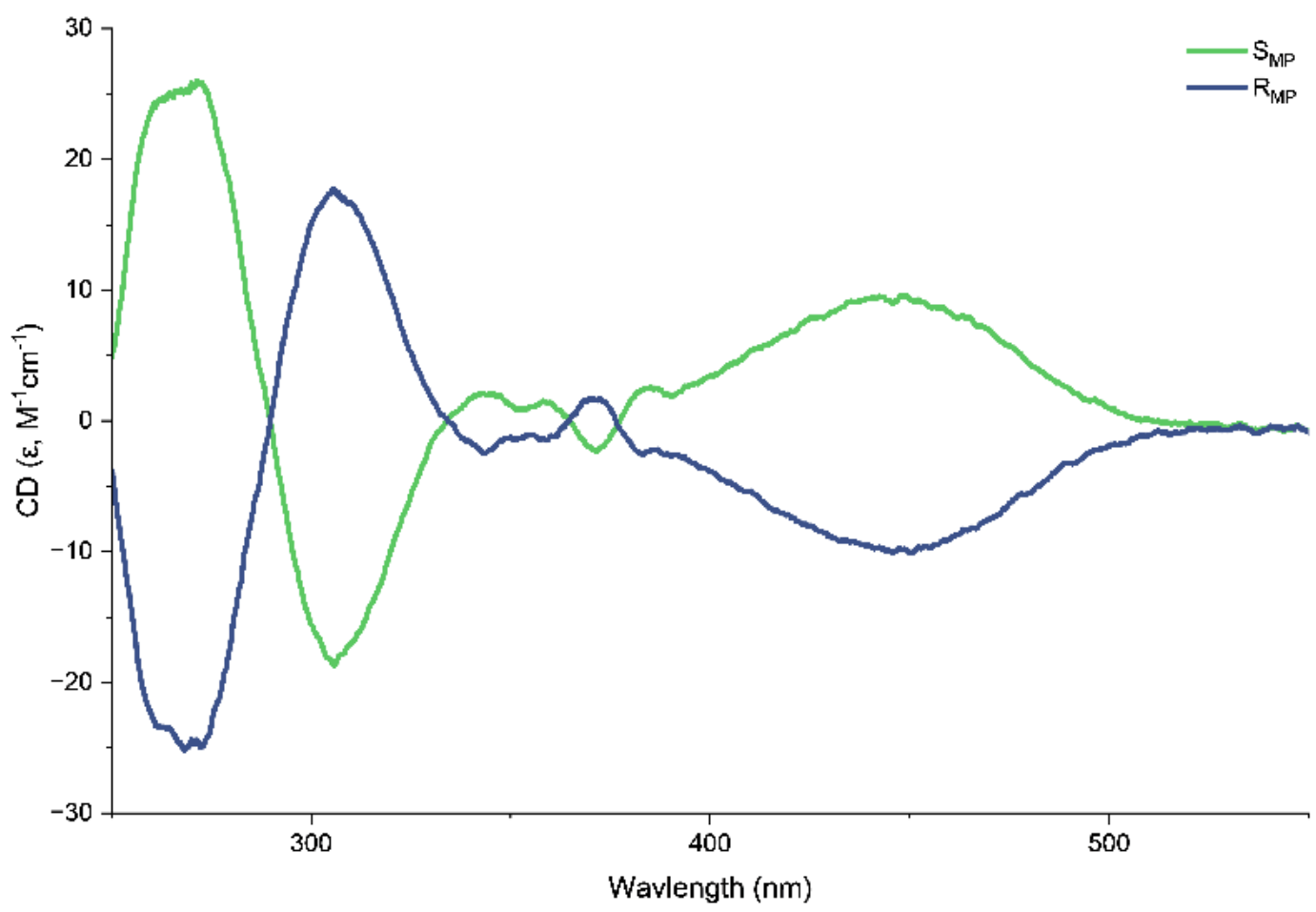

Figure S2: CD spectra of both enantiomers of NDI rotaxane **1** in chloroform at 15 μM

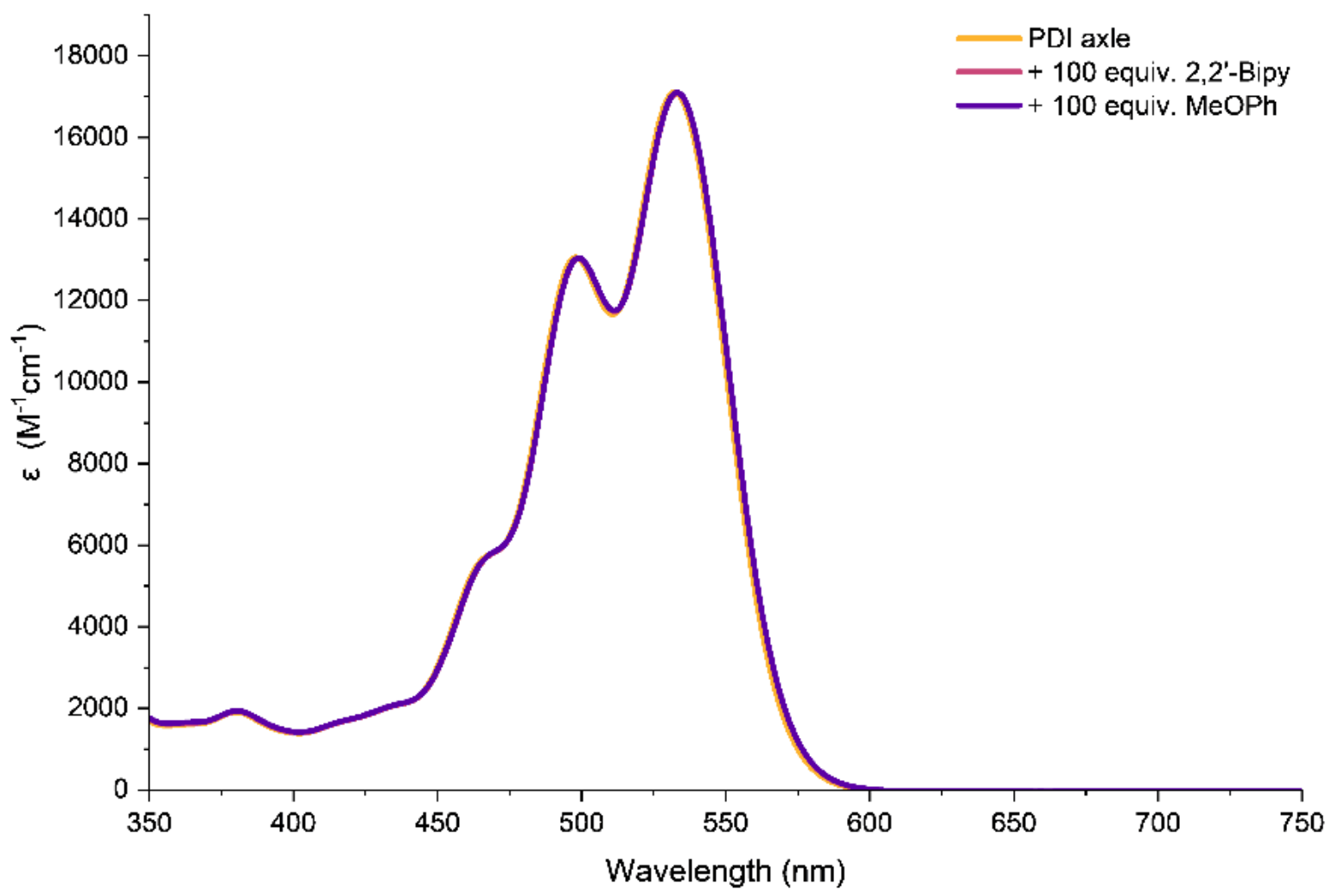

Figure S3: UV-vis spectra of PDI free axle (**g**) in chloroform at 20 µM before and after the addition of an excess of 2,2'-bipyridine and methoxybenzene

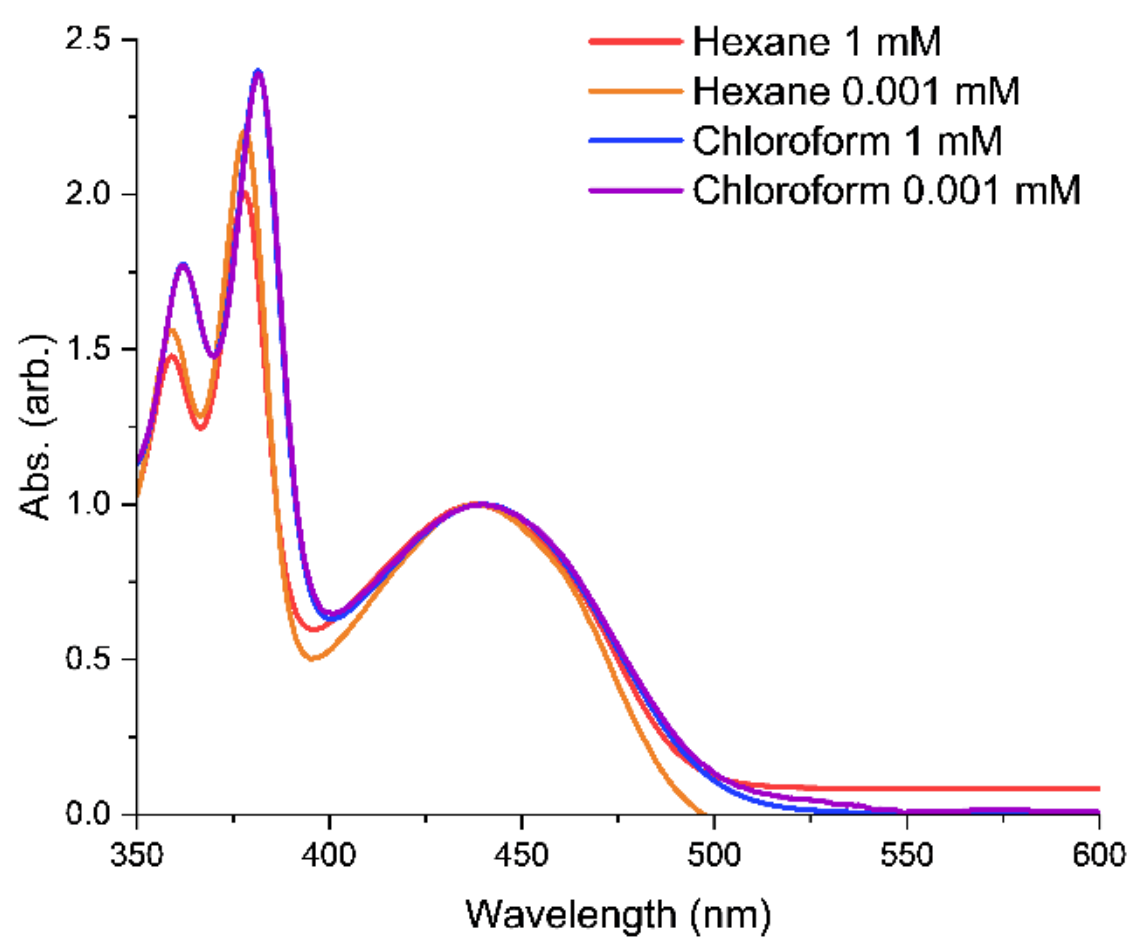

Figure S4: UV-vis spectra of NDI [2]rotaxane **1** in an apolar (hexane) and polar (chloroform) solvent at high and low concentrations

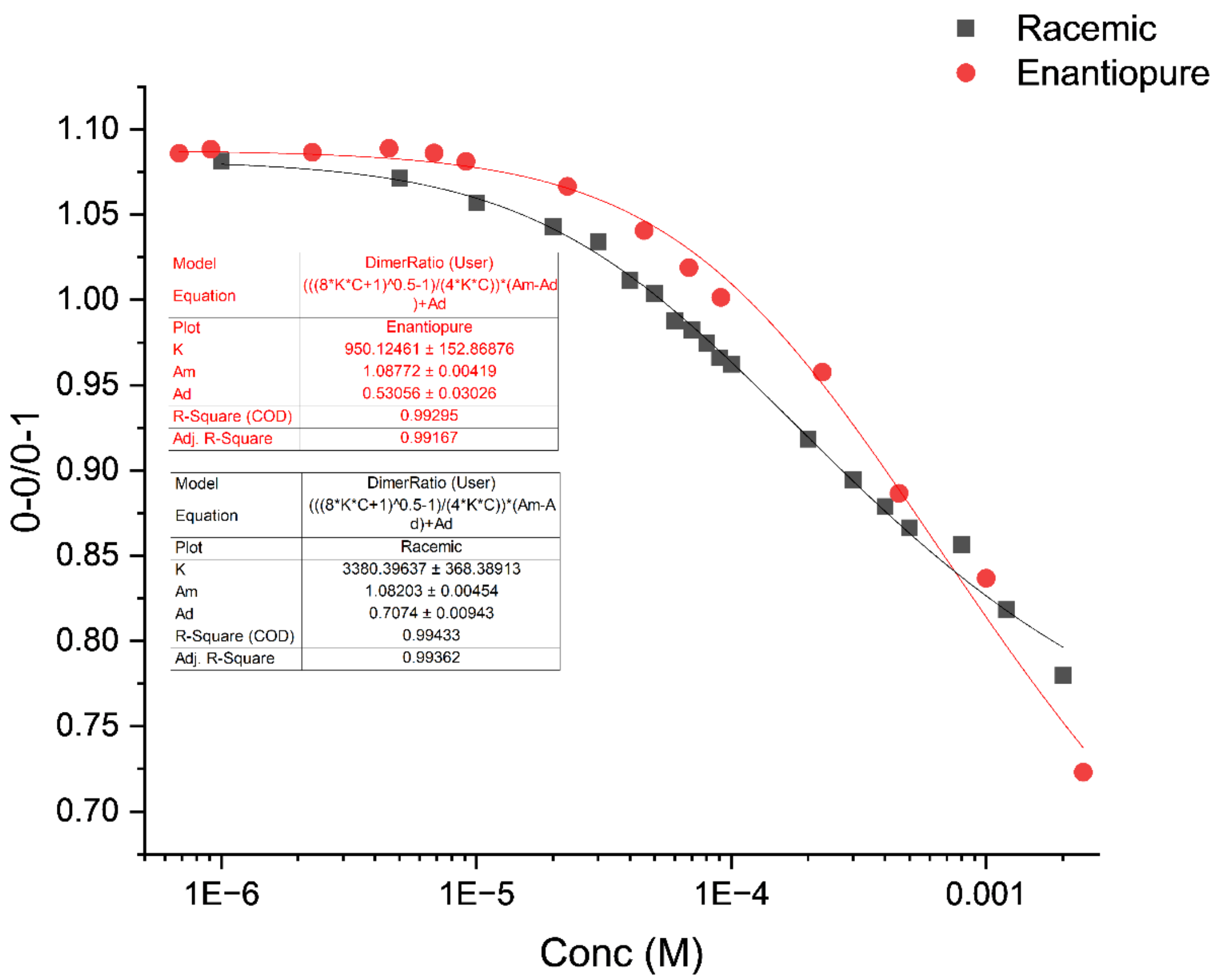

Figure S5: 0-0/0-1 ratio of racemic and enantiopure samples of PDI [2]rotaxane (**2**) plotted against the sample concentration in n-hexane, with each data set fitted to the dimer aggregation model.[5]

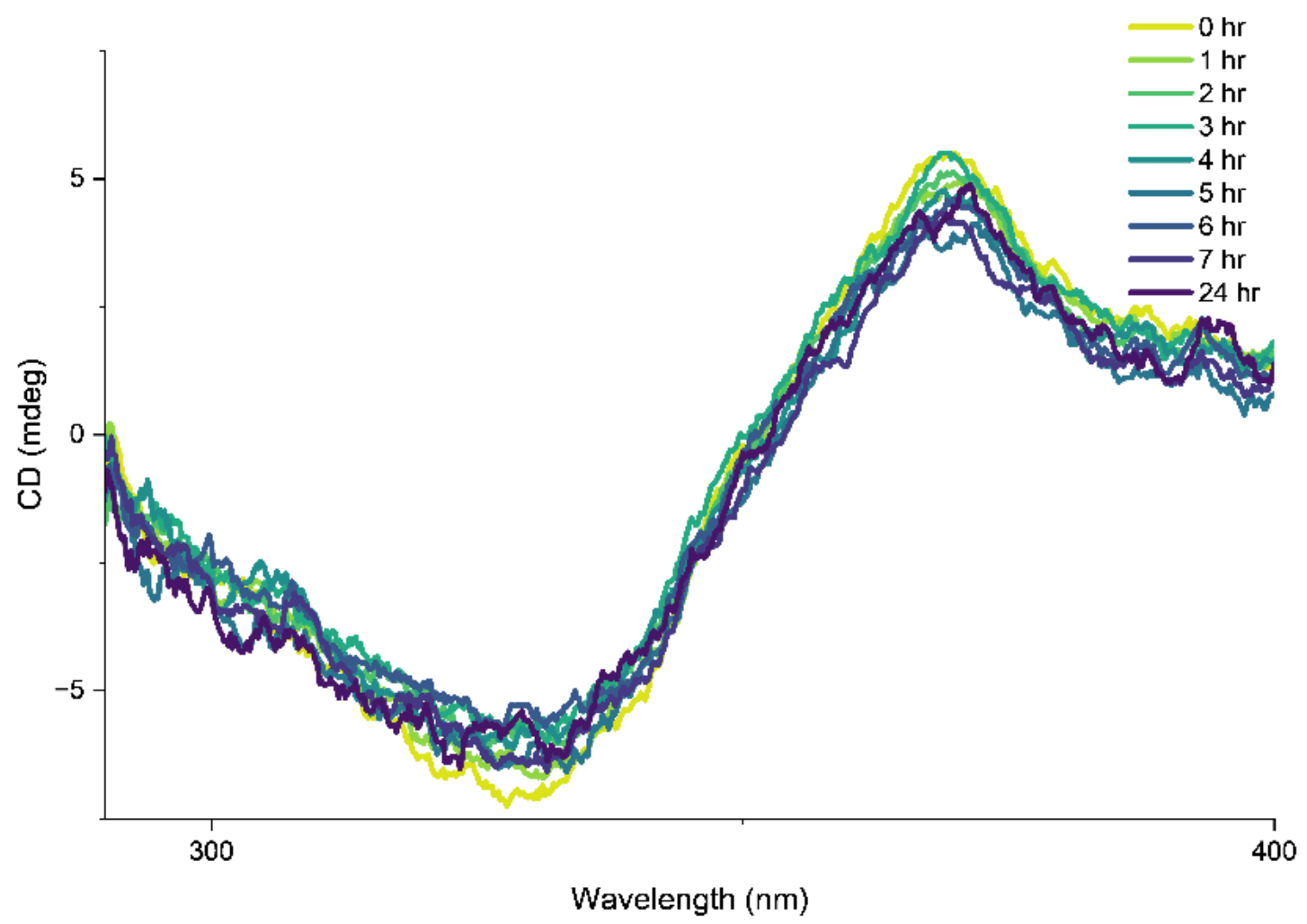

Figure S6: CD spectra of PDI rotaxane **2-*R*$_{mp}$** in 1,1,2,2-tetrachloroethane (5 μM) taken at regular intervals whilst heating at 120 °C over 24 hours.

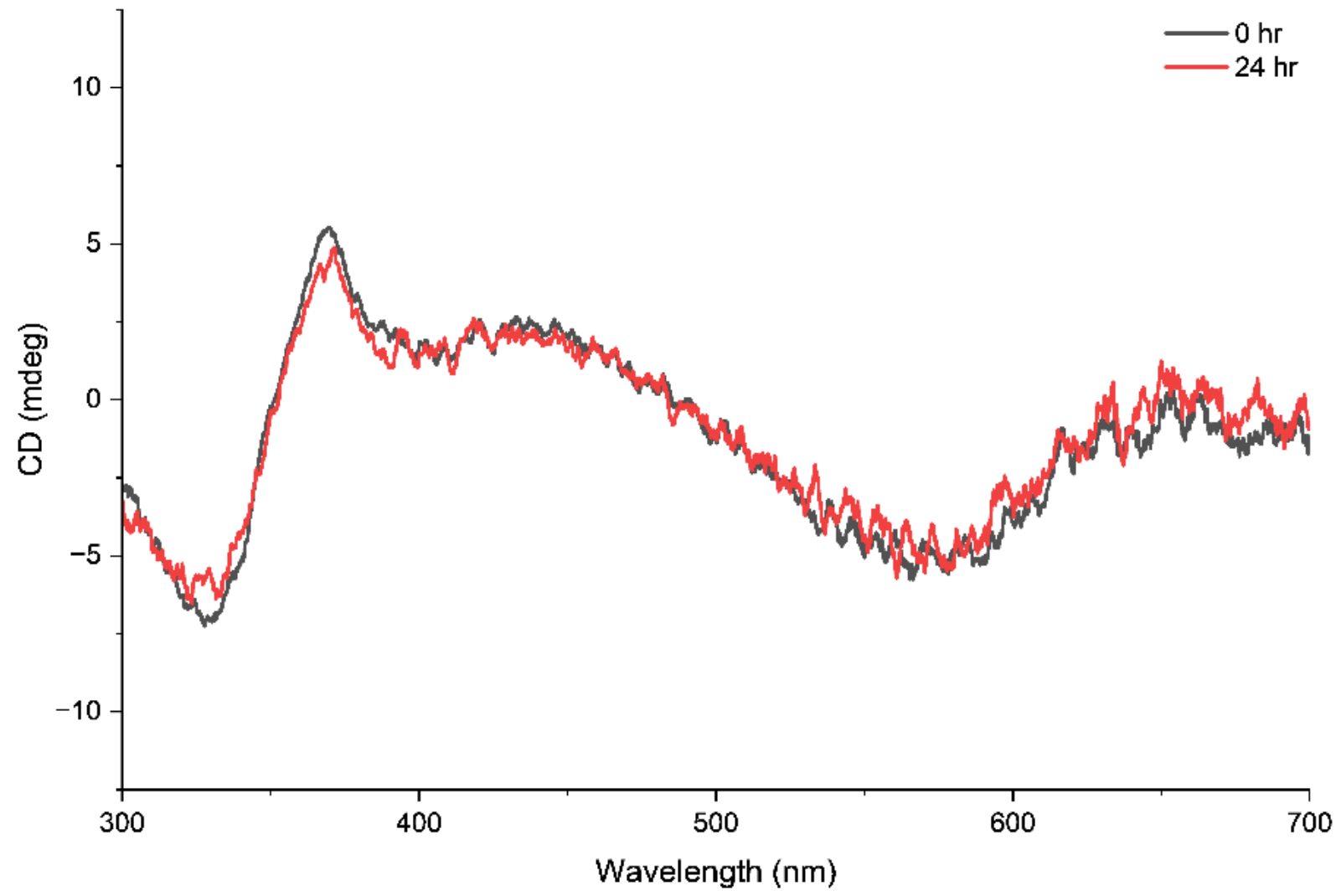

Figure S7: CD spectra of PDI [2]rotaxane **2-*R*$_{mp}$** in 1,1,2,2-tetrachloroethane (5 μM) before and after heating at 120 °C for 24 hours.

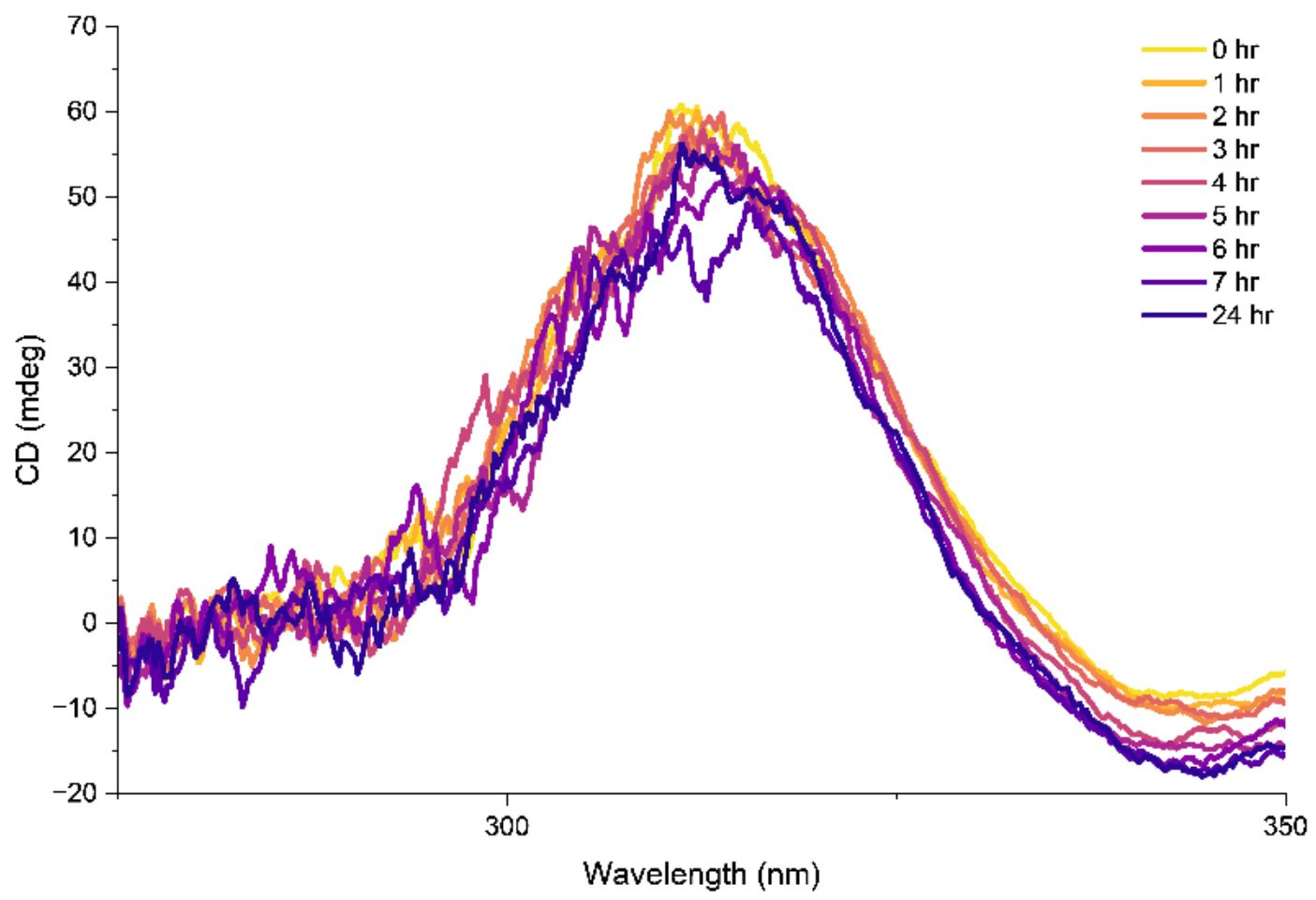

Figure S8: CD spectra of NDI rotaxane **1-*R*$_{mp}$** in 1,1,2,2-tetrachloroethane (30 μM) taken at regular intervals whilst heating at 120 °C over 24 hours.

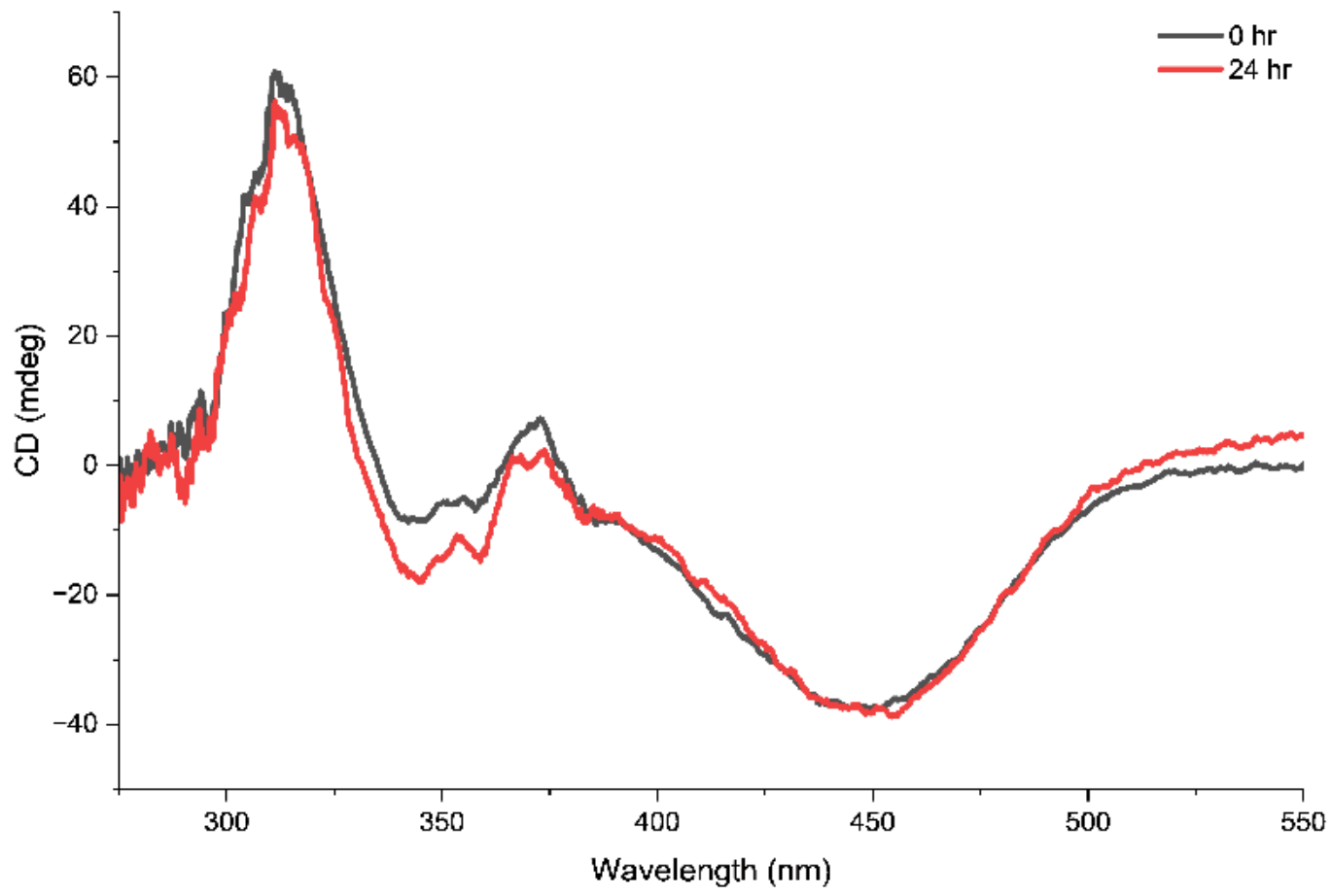

Figure S9: CD spectra of NDI [2]rotaxane (**1-*R*$_{\mathbf{mp}}$**) in 1,1,2,2-tetrachloroethane (30 μM) before and after heating at 120 °C for 24 hours.

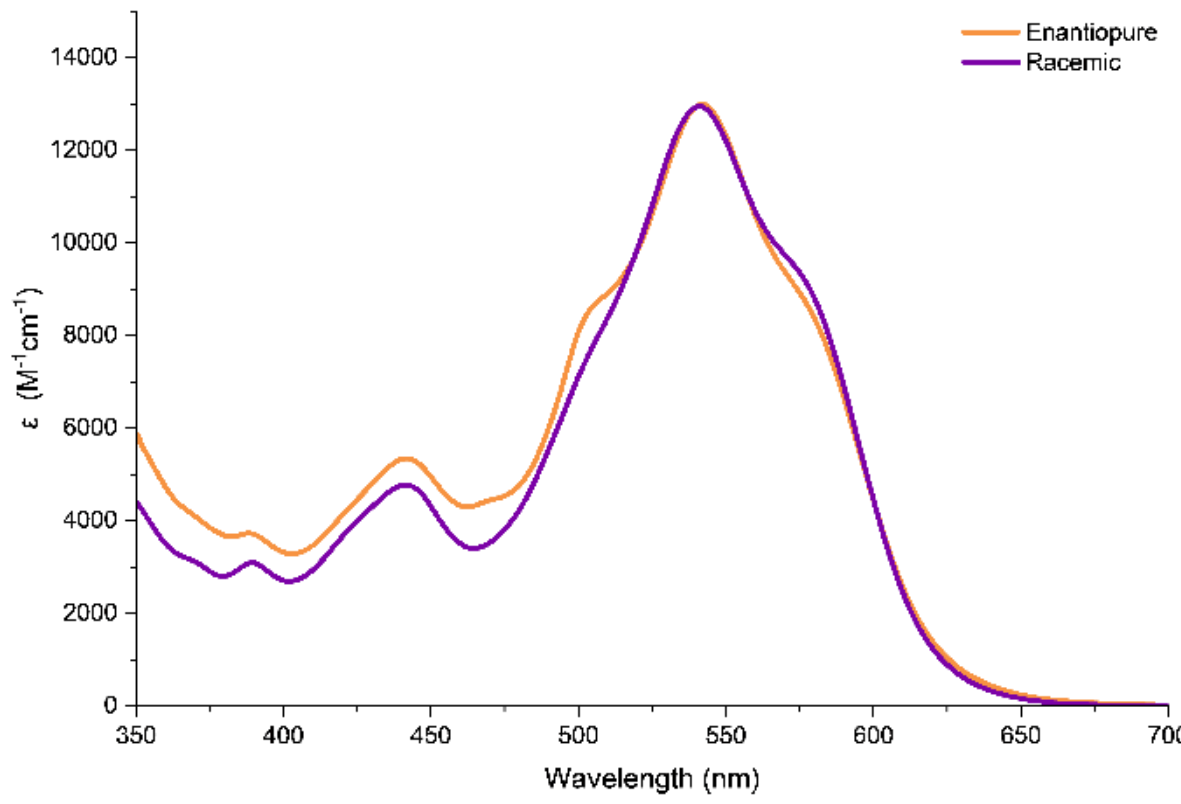

Figure S10: UV-Vis spectra of enantiopure and racemic PDI [2]rotaxane (**2**) at 2 mM in *n*-hexane

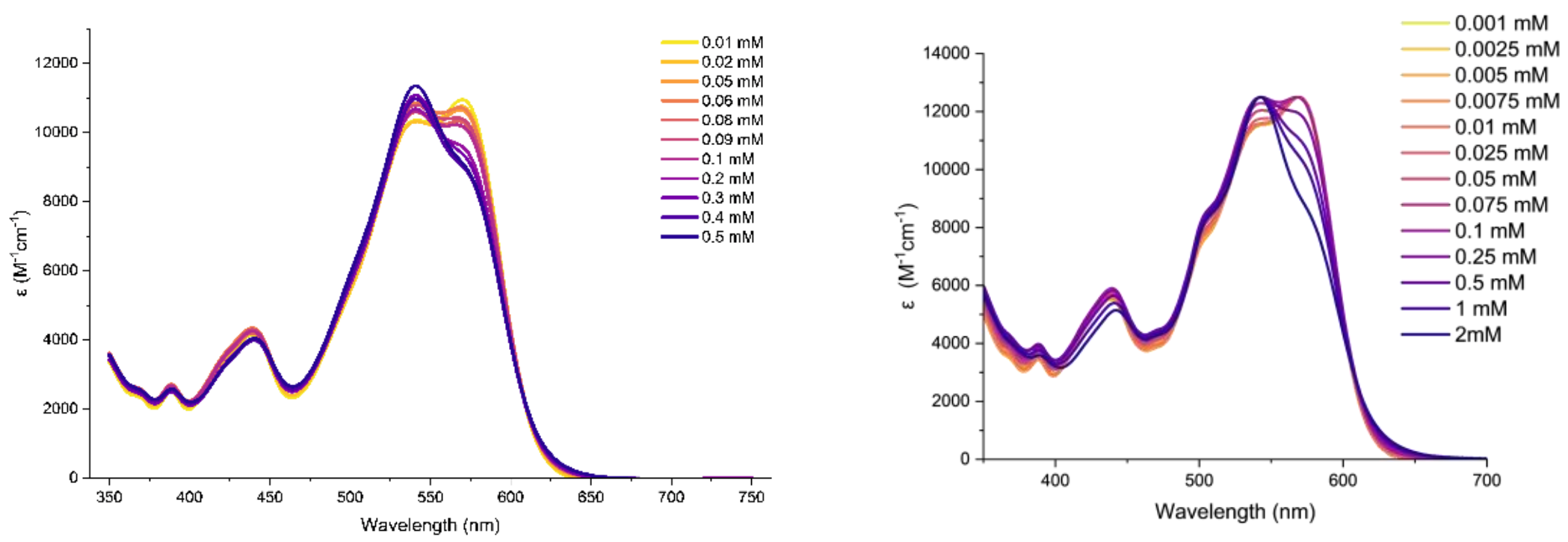

Figure S11: UV-vis spectra of racemic (left) and enantiopure (right) PDI [2]rotaxane (**2**) in *n*-hexane at various concentrations

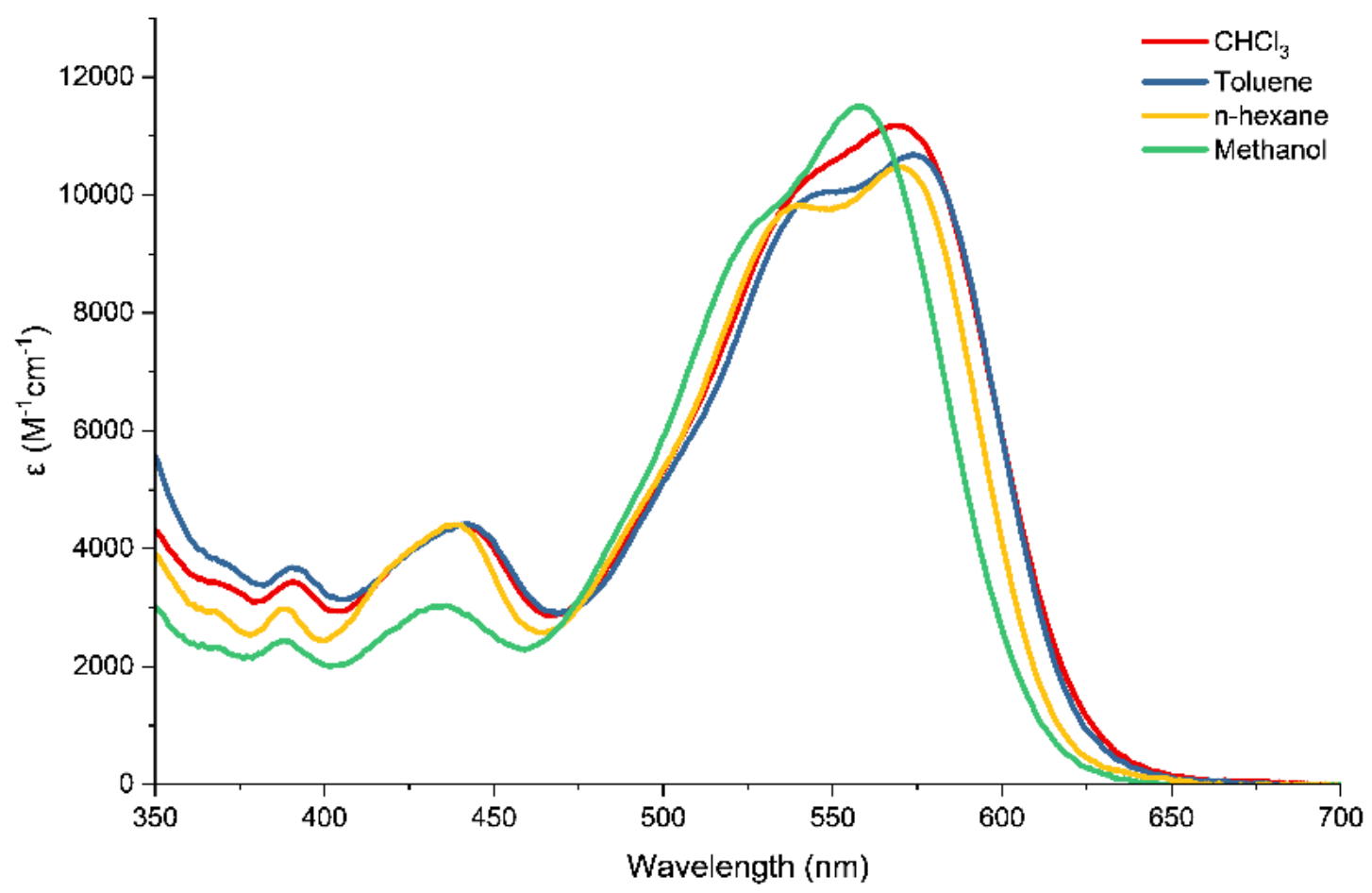

Figure S12: UV-Vis spectra of racemic PDI [2]rotaxane (**2**) at 20 μM in various solvents

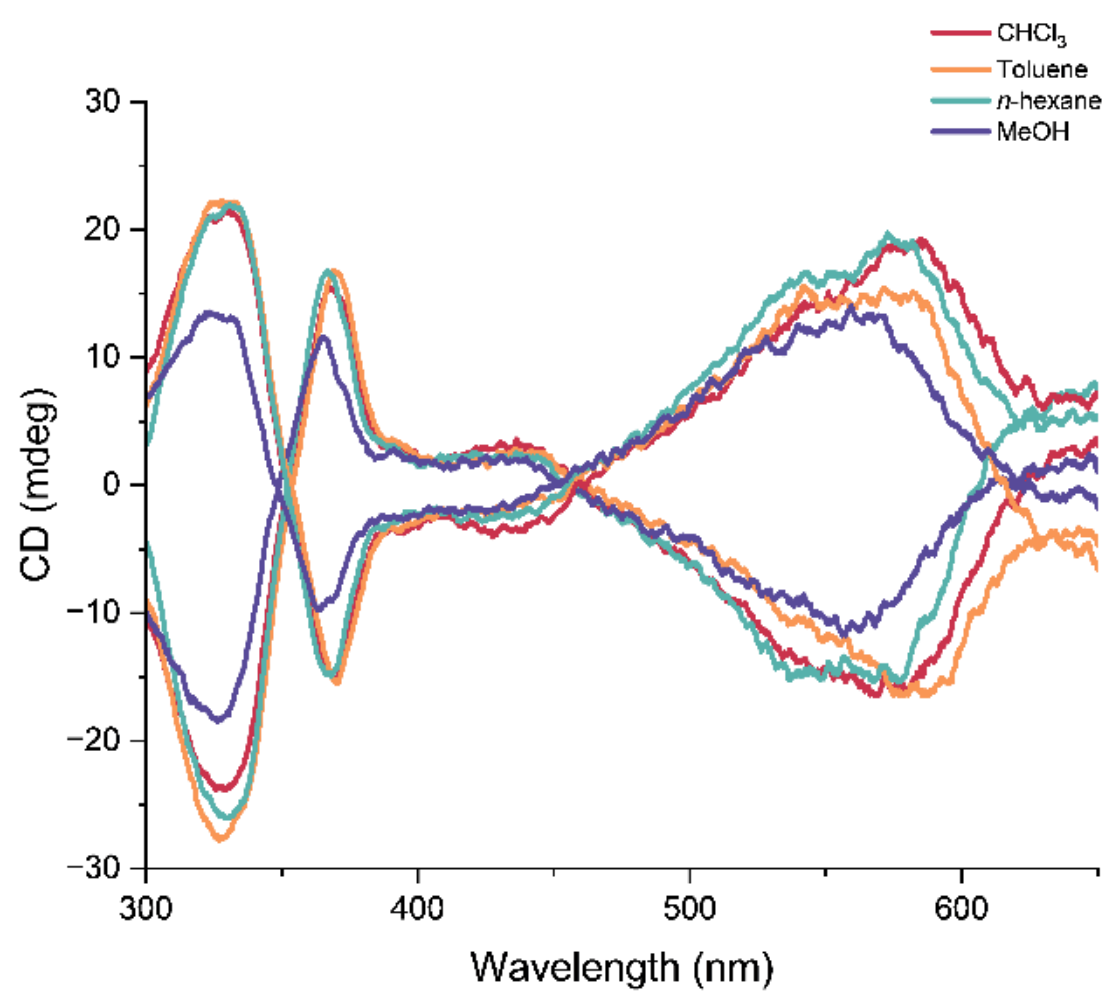

Figure S13: CD spectra of PDI [2]rotaxane **2** at 20 μM in various solvents

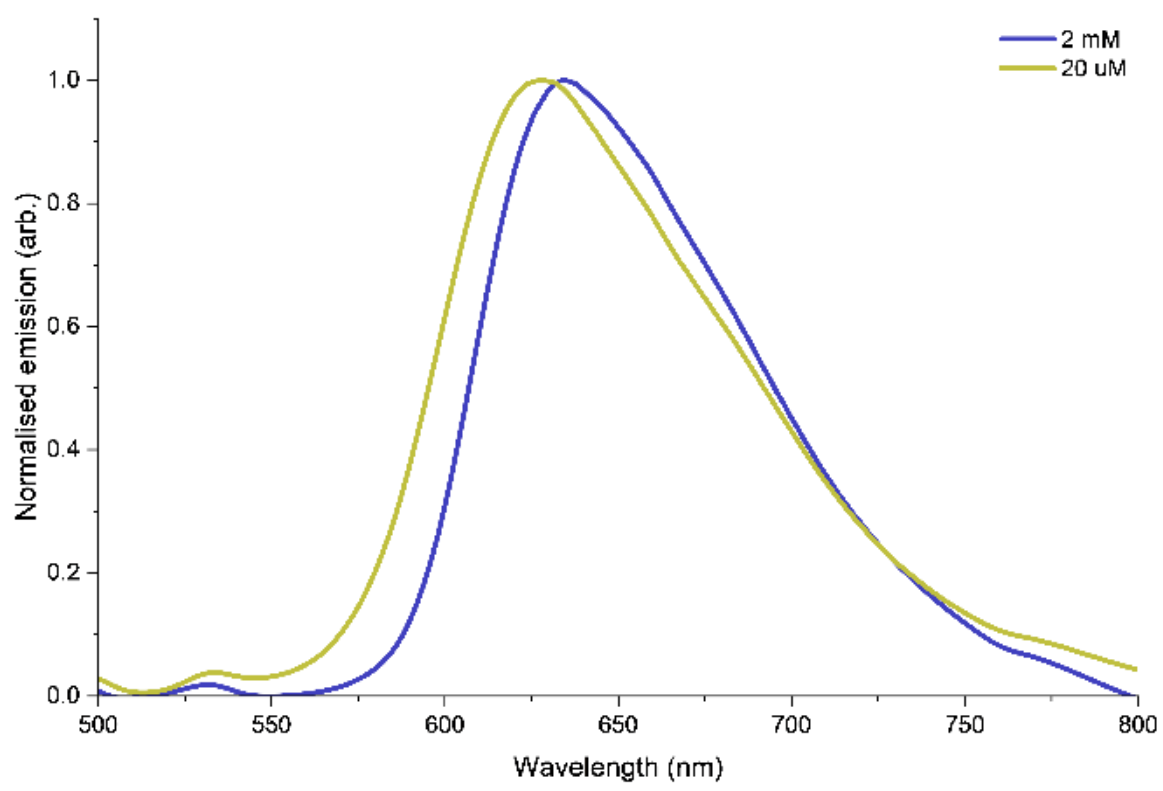

Figure S14: PL spectra (λ_{ex} = 475 nm) of PDI [2]rotaxane **2** at 20 μM and 2 mM in *n*-hexane

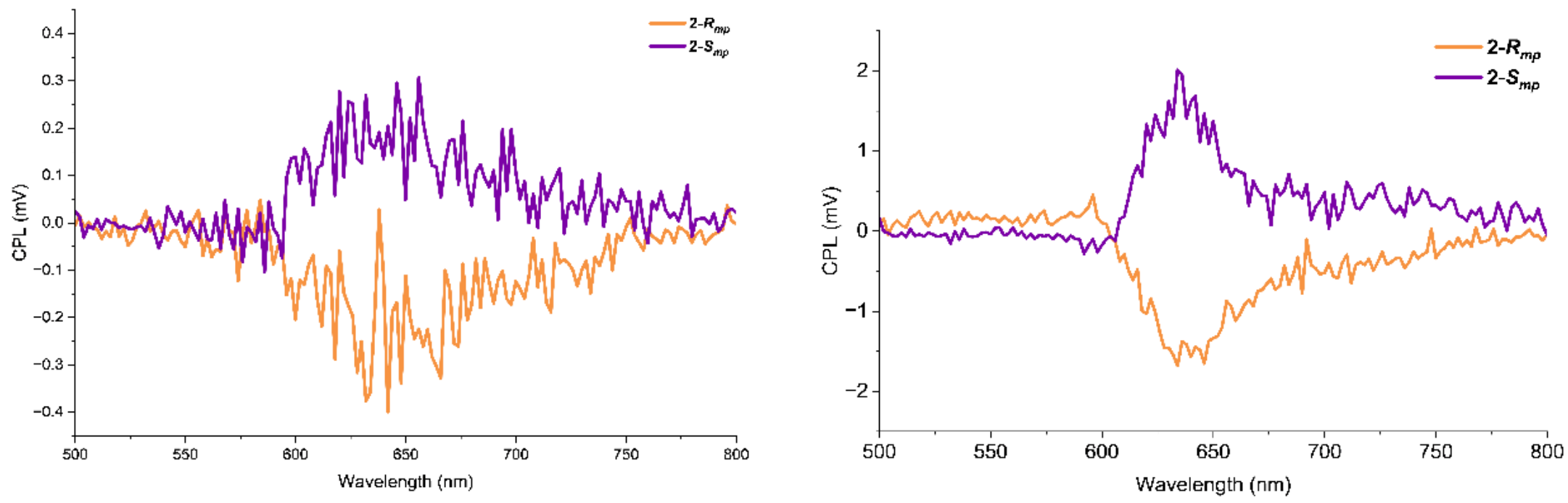

Figure S15: CPL spectra (λ_{ex} = 475 nm) of PDI [2]rotaxane (**2**) at 20 μM (left) and 2 mM (right) in *n*-hexane

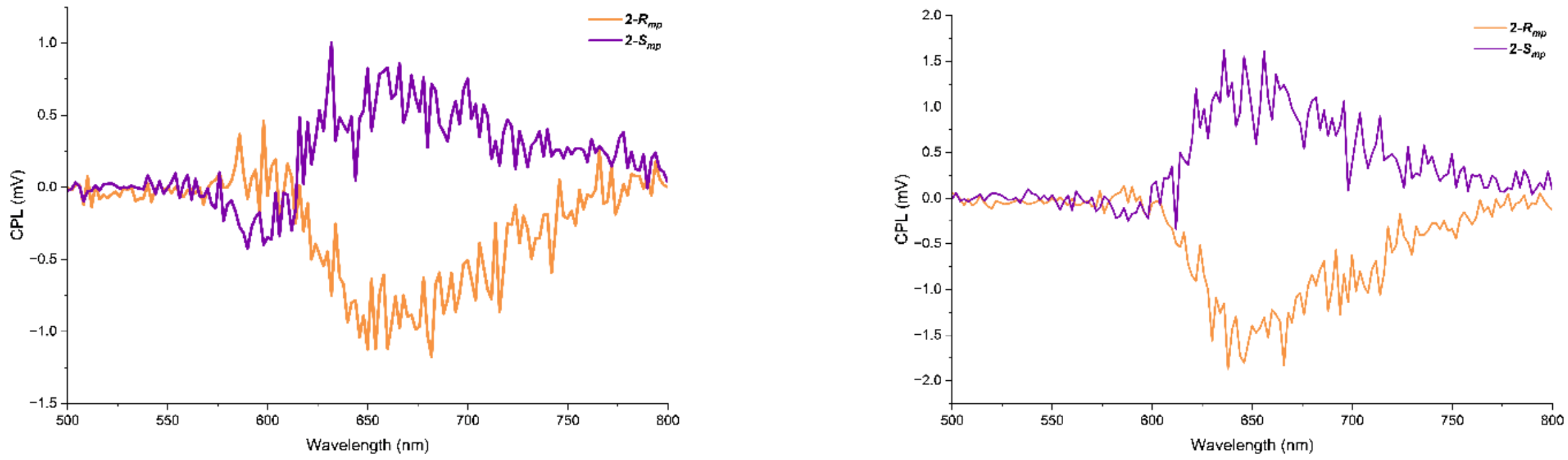

Figure S16: CPL spectra (λ_{ex} = 475 nm) of PDI [2]rotaxane (**2**) at 100 μM in chloroform (left) and toluene (right)

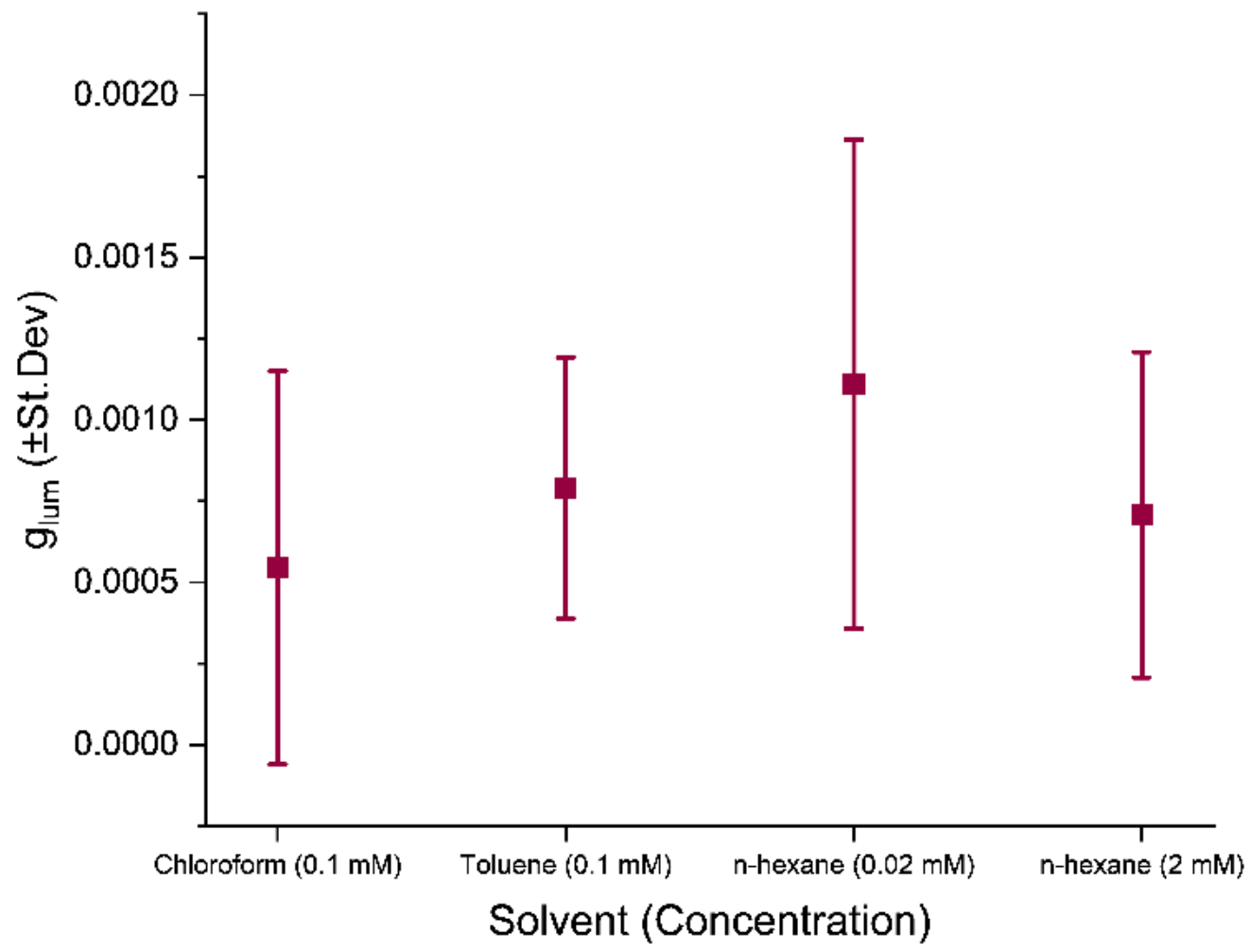

Figure S17: The g_{lum} values of PDI [2]rotaxane (**2**) recorded in three different solvents and at two different concentrations in *n*-hexane.

3) Further ^{1}H NMR Spectroscopy

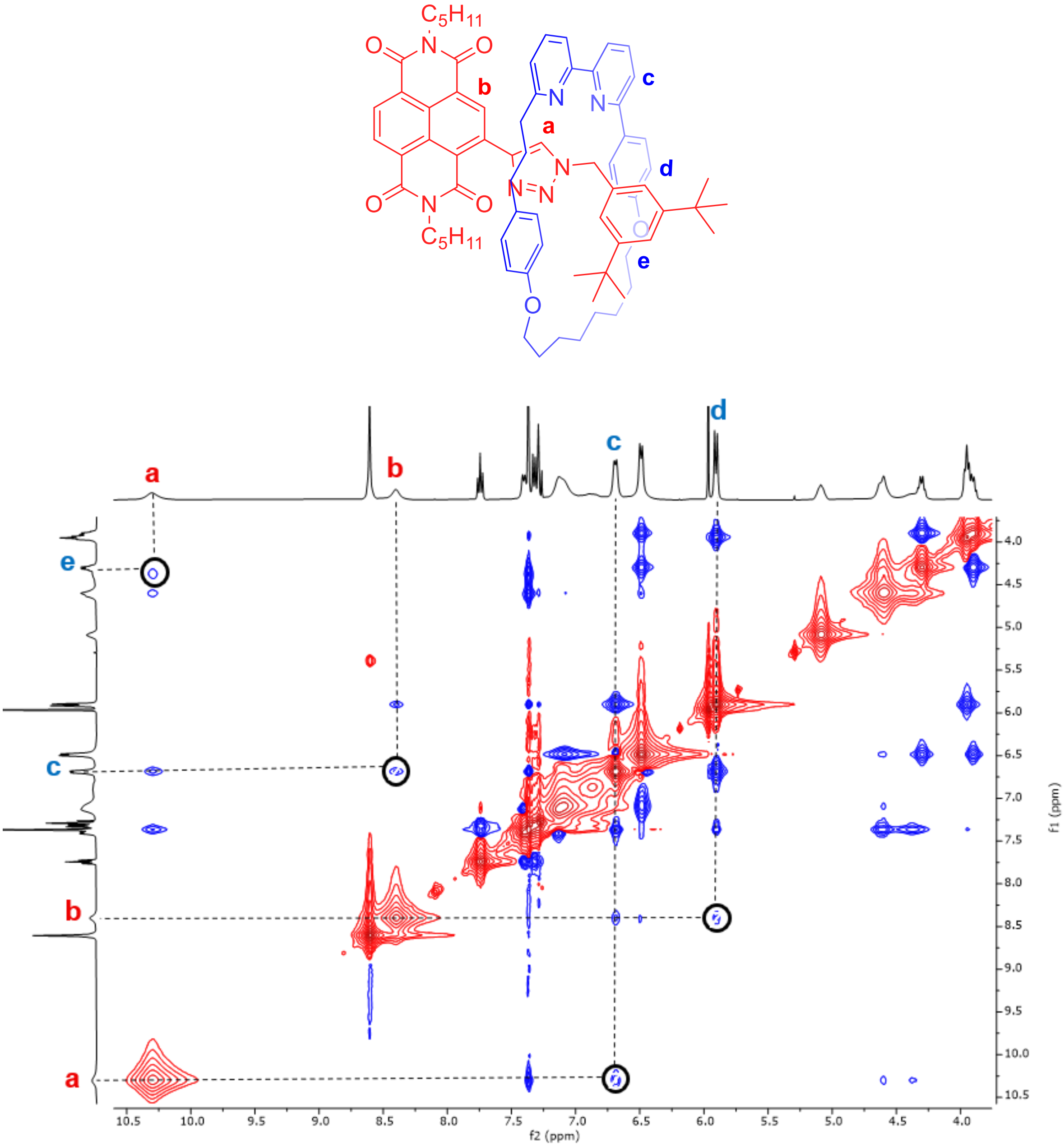

Figure S18: ^{1}H-^{1}H ROESY NMR spectra of NDI [2]rotaxane (**1**) (TCE-*d2*, 298 K, 400 MHz)

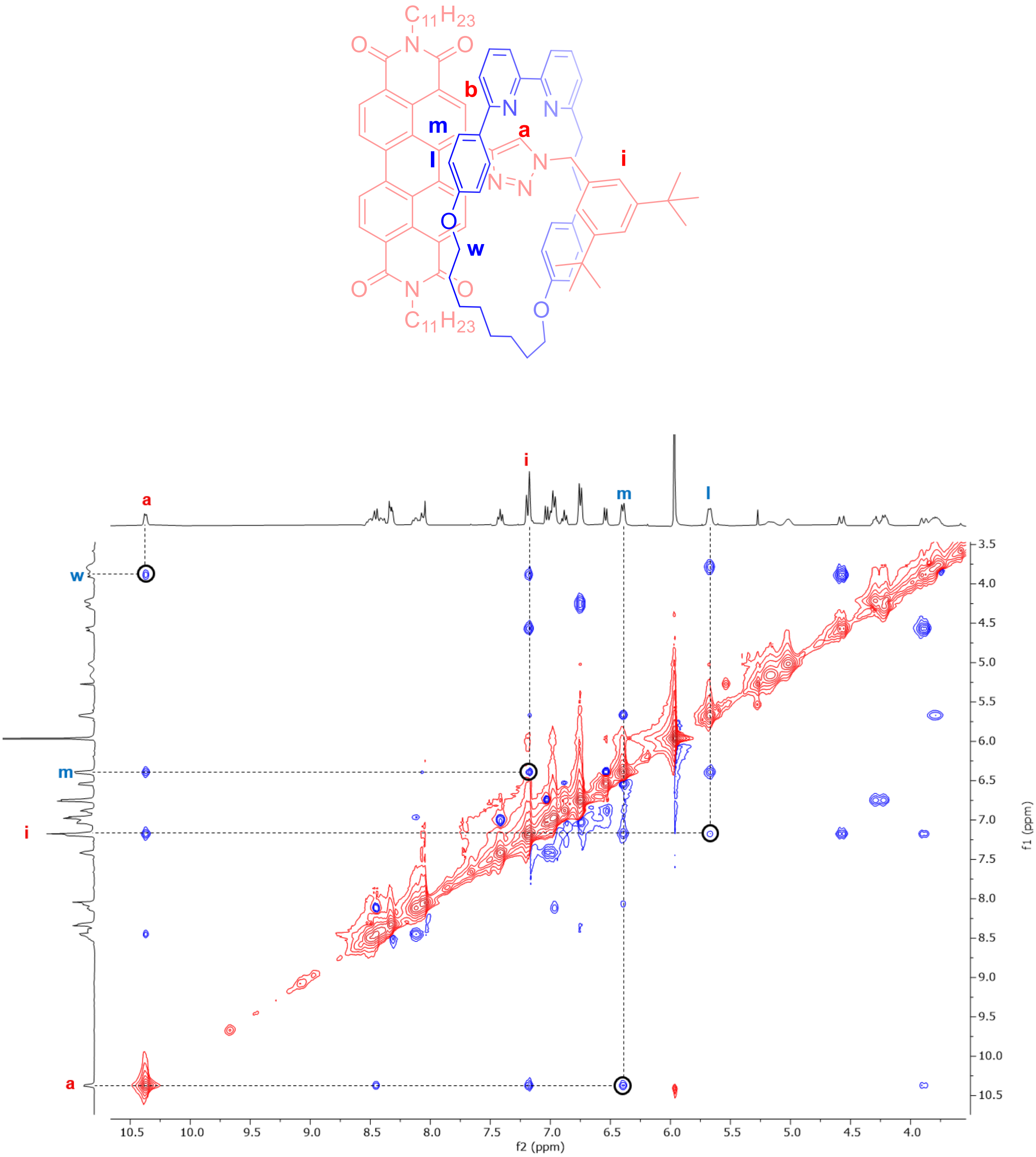

Figure S19: ^{1}H-^{1}H ROESY NMR spectra of PDI [2]rotaxane (**2**) (TCE-*d2,* 298 K, 400 MHz)

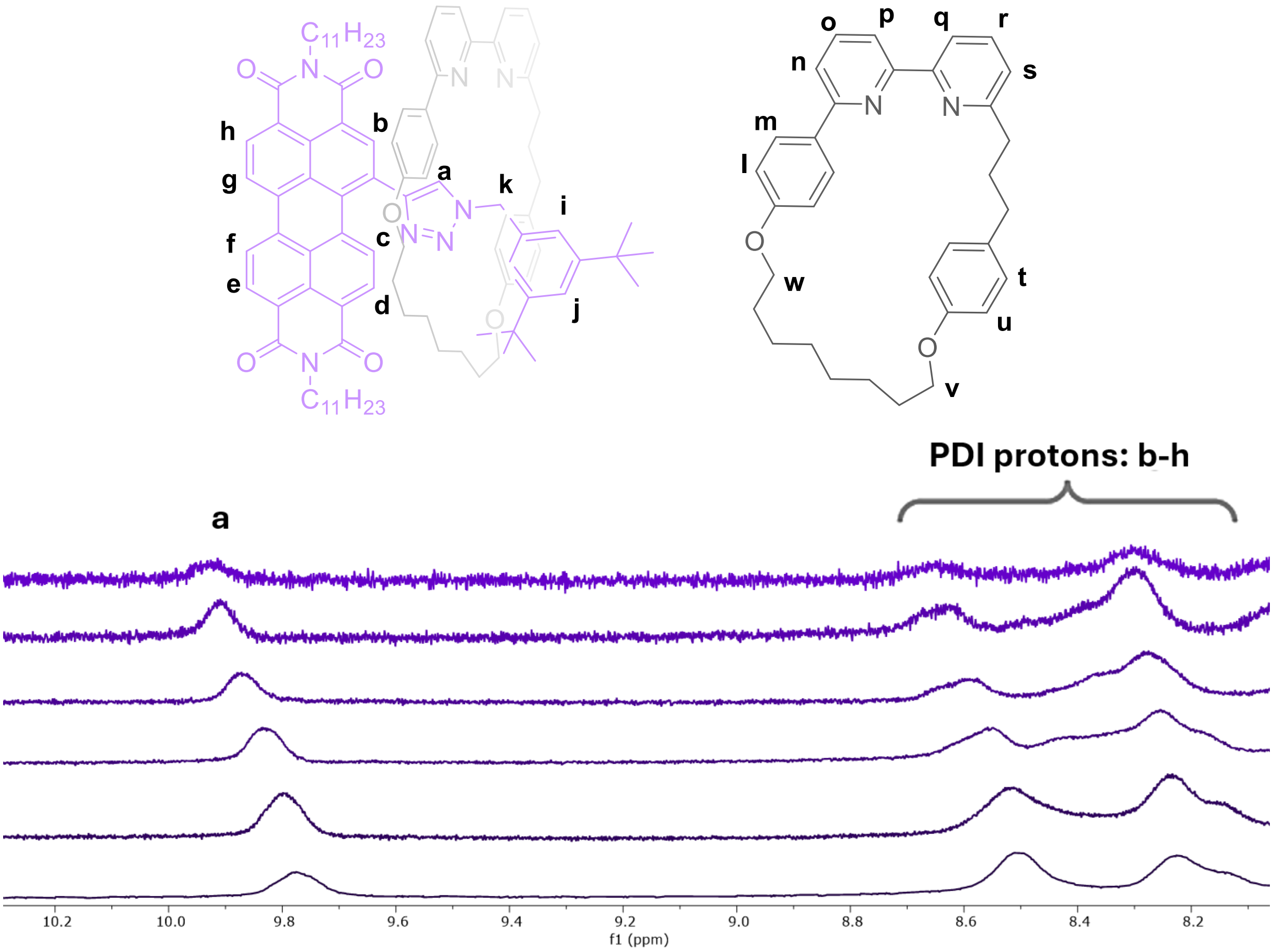

Figure S20: ^{1}H NMR spectra of PDI [2]rotaxane (**2**) (cyclohexane-*d12*, 298 K, 400 MHz) at various concentrations, (27.4 mM [bottom] – 0.8 mM [top])

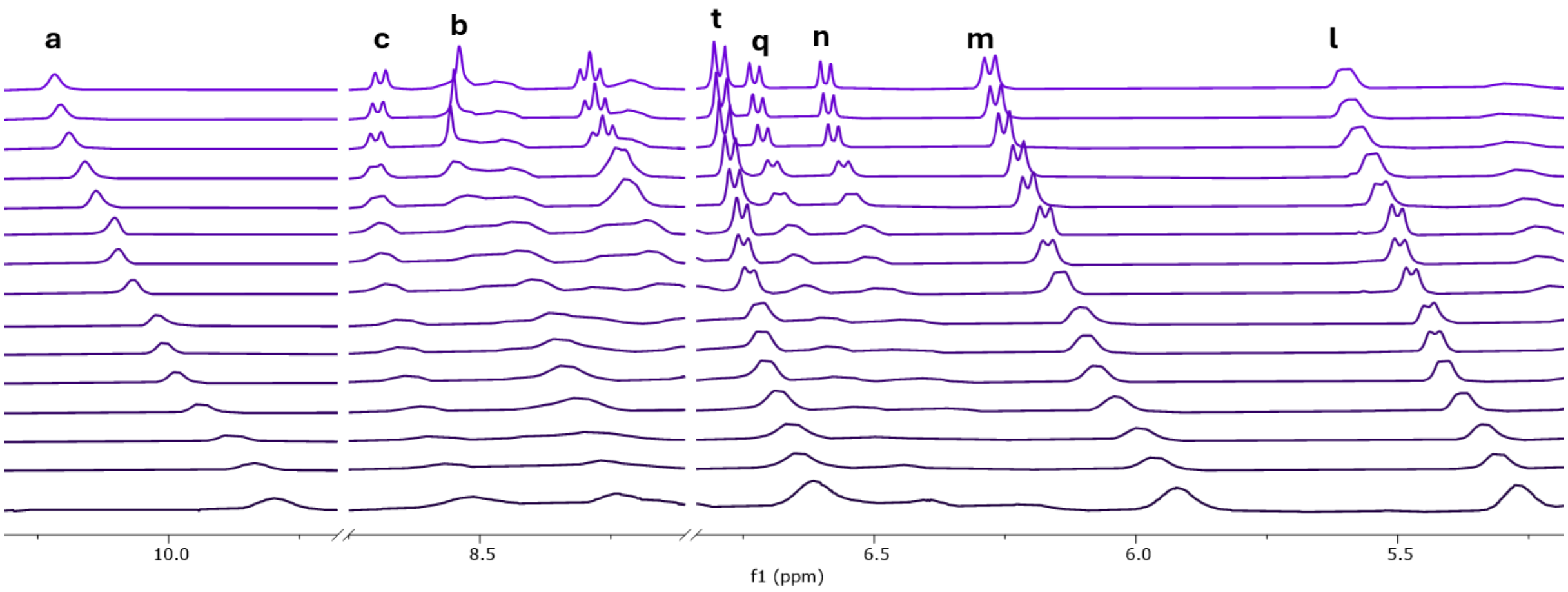

Figure S21: ^{1}H NMR spectroscopic titration of $CDCl_3$ into a solution of PDI [2]rotaxane (**2**) (cyclohexane-*d12*, 298 K, 400 MHz), from 0% $CDCl_3$ (bottom) to 33% $CDCl_3$ (top).

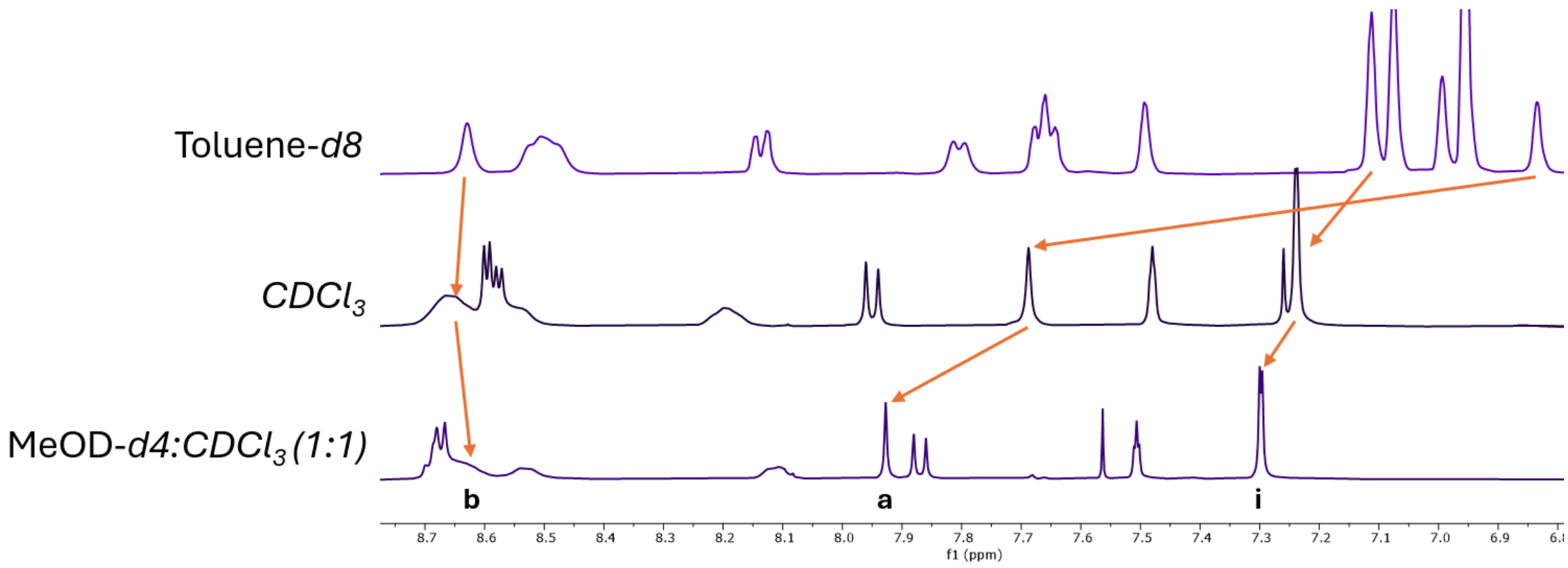

Figure S22: ^{1}H NMR spectra of PDI axle (**g**) in various solvents, with diagnostic peaks highlighted

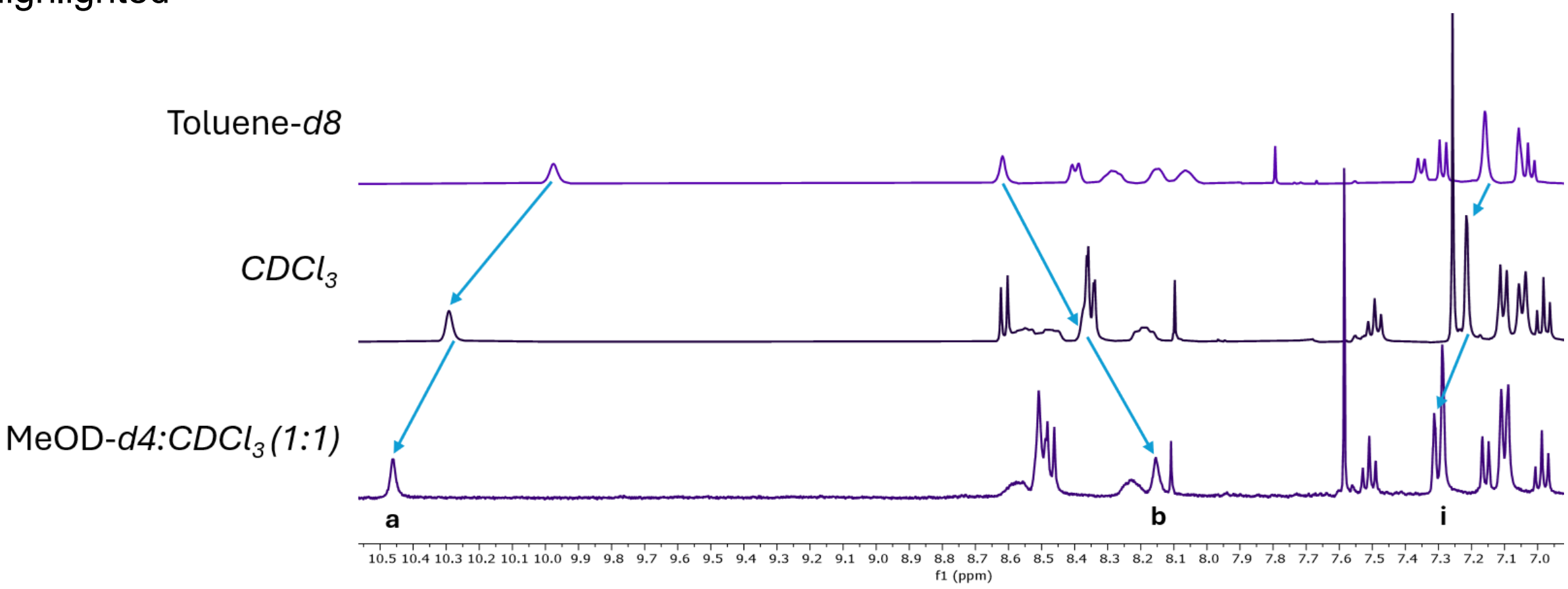

Figure S23: ^{1}H NMR spectra of PDI [2]rotaxane (**2**) in various solvents, with diagnostic peaks highlighted

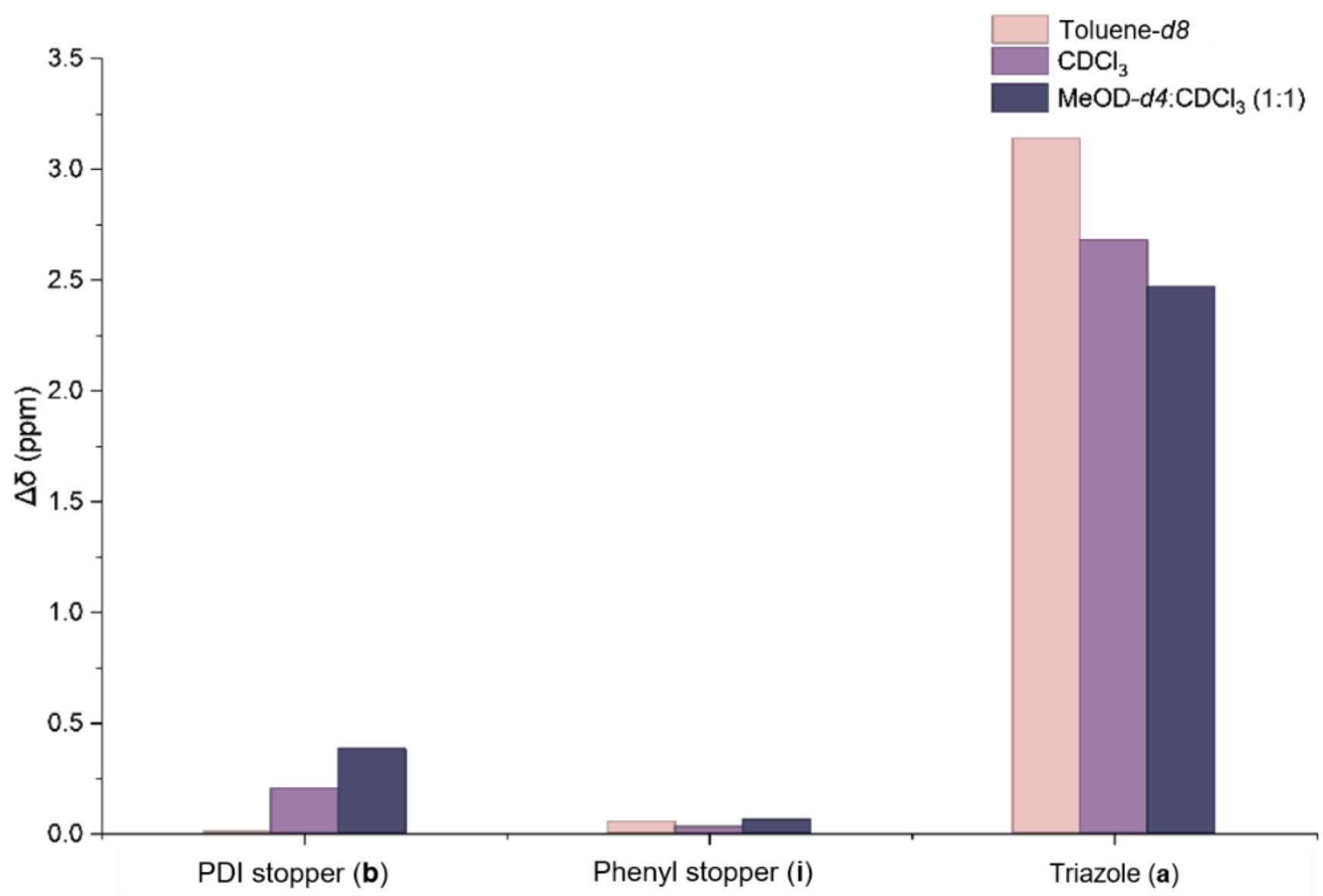

Figure S24: The difference in chemical shift (Δδ) of proton signals in PDI [2]rotaxane (**2**) and PDI free axle measured in various solvents.

Table S1: The difference in chemical shift (Δδ) of proton signals in PDI rotaxane **2** and PDI free axle measured in various solvents (red/yellow/green = large/medium/small shift).

	Toluene-*d8*	$CDCl_3$	MeOD-*d4*:$CDCl_3$
PDI stopper (**b**)	0.01	0.20	0.38
Phenyl stopper (**i**)	0.05	0.03	0.06
Triazole (**a**)	3.14	2.68	2.47

While the largest Δδ values are for H_a and indicate that the macrocycle resides at the triazole 'station' (Table S1), we note that the magnitude of Δδ decreases slightly (from 3.1 to 2.5 ppm; Fig. S24) with increasing solvent polarity and hydrogen-bonding ability. Examination of the stopper protons at either end of the axle reveals that Δδ increases for the PDI resonance H_b (from 0.01 to 0.38 ppm), whereas the phenyl stopper signal H_i remains largely unchanged (0.03 - 0.06 ppm). These observations suggest a greater tendency for the macrocycle to interact with the PDI unit, through π–π stacking interactions (Figs. 2 and 3), as solvent polarity increases. This is consistent with the solvent dependence of π–π interactions, which may be stronger in polar protic solvents such as MeOH through a combination of dispersion and solvophobic effects.[6]

4) Chiral HPLC

To resolve the enantiomers of [2]rotaxanes **1** and **2**, chiral HPLC was performed on a Phenomenex Lux 5 µm i-Amylose-1 (C 250 x 4.6 mm) column using a gradient of 0-5% IPA in *n*-hexane over 24 minutes at a flow rate of 1 ml/min.

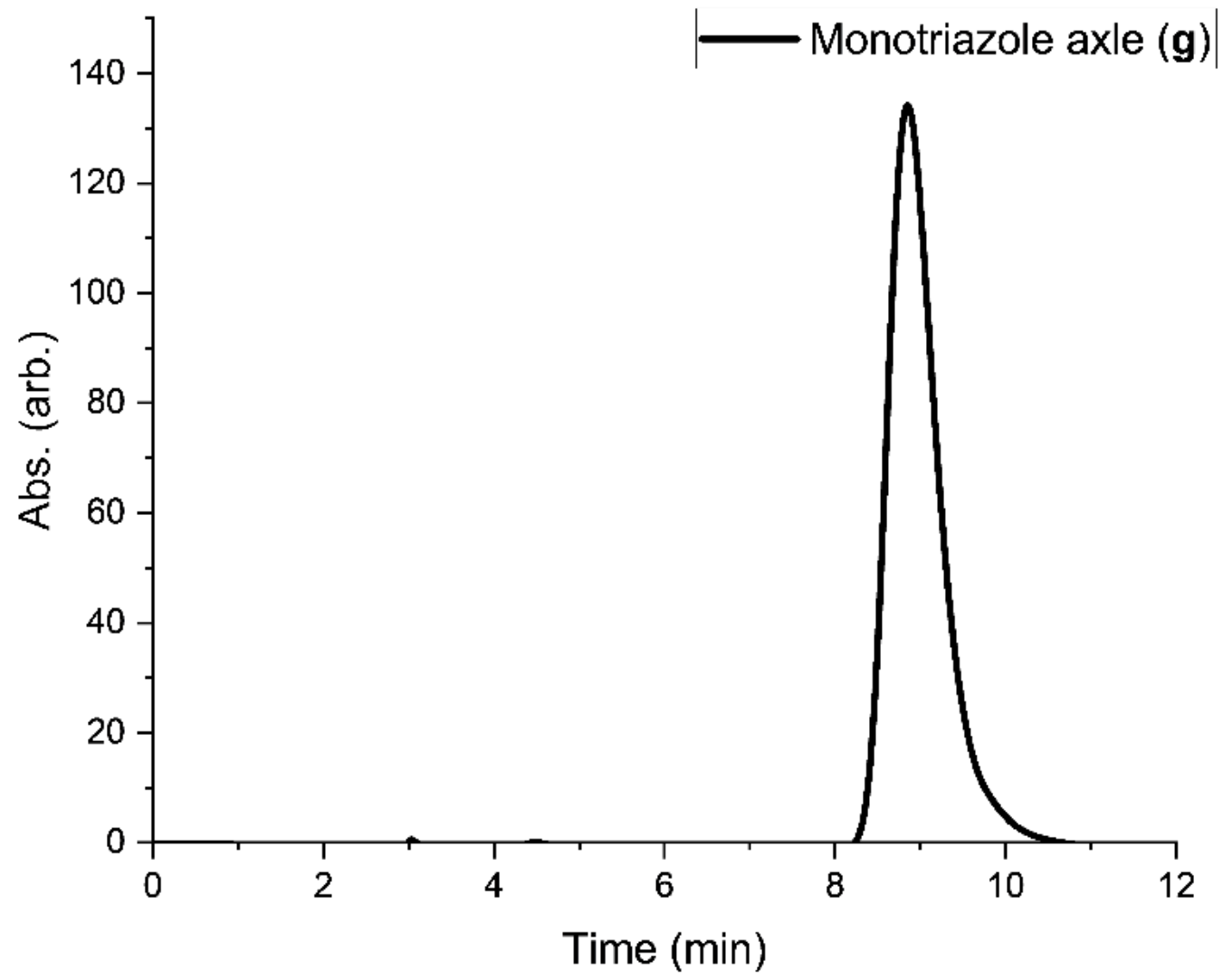

Figure S25: Chromatogram of the PDI axle (**g**)

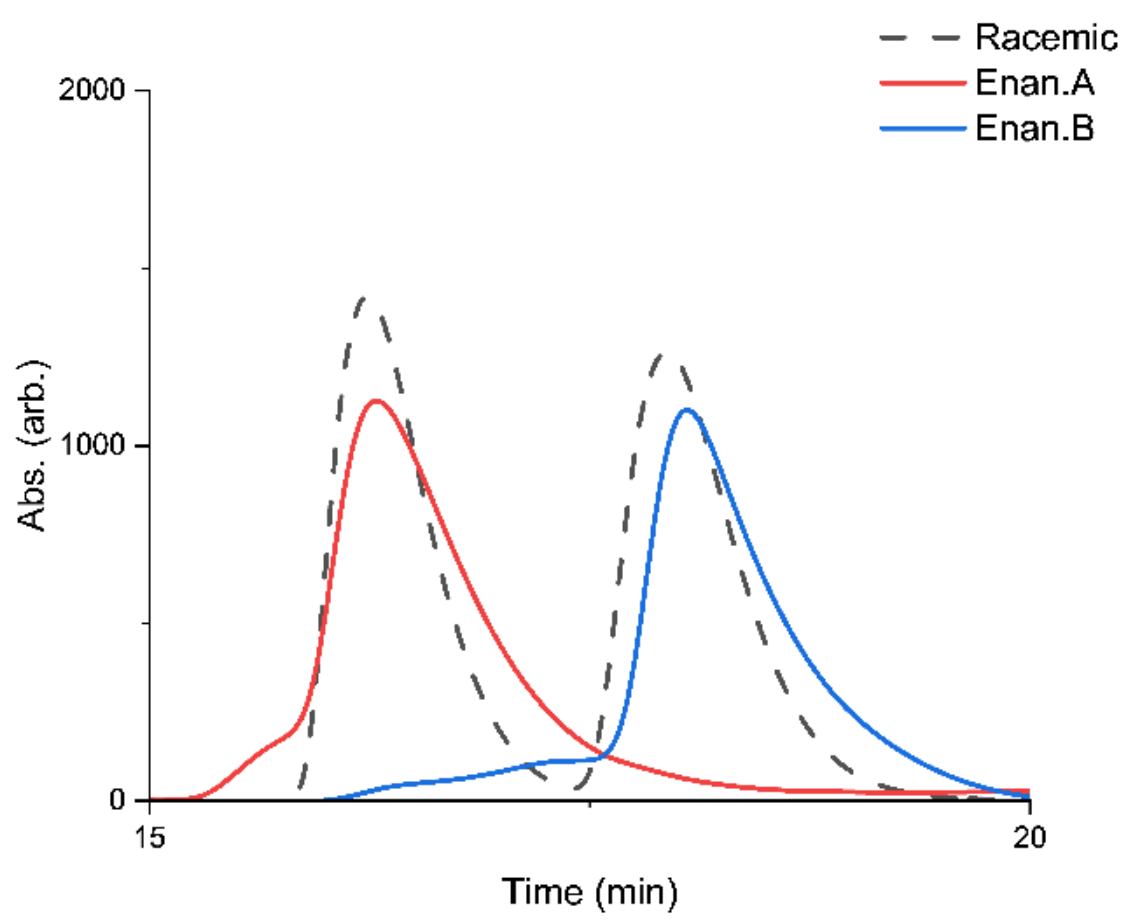

Figure S26: Chromatogram of the PDI [2]rotaxane (**2**) as a racemic mixture (dashed black trace), and reinjection of each isolated enantiomer (solid red and blue)

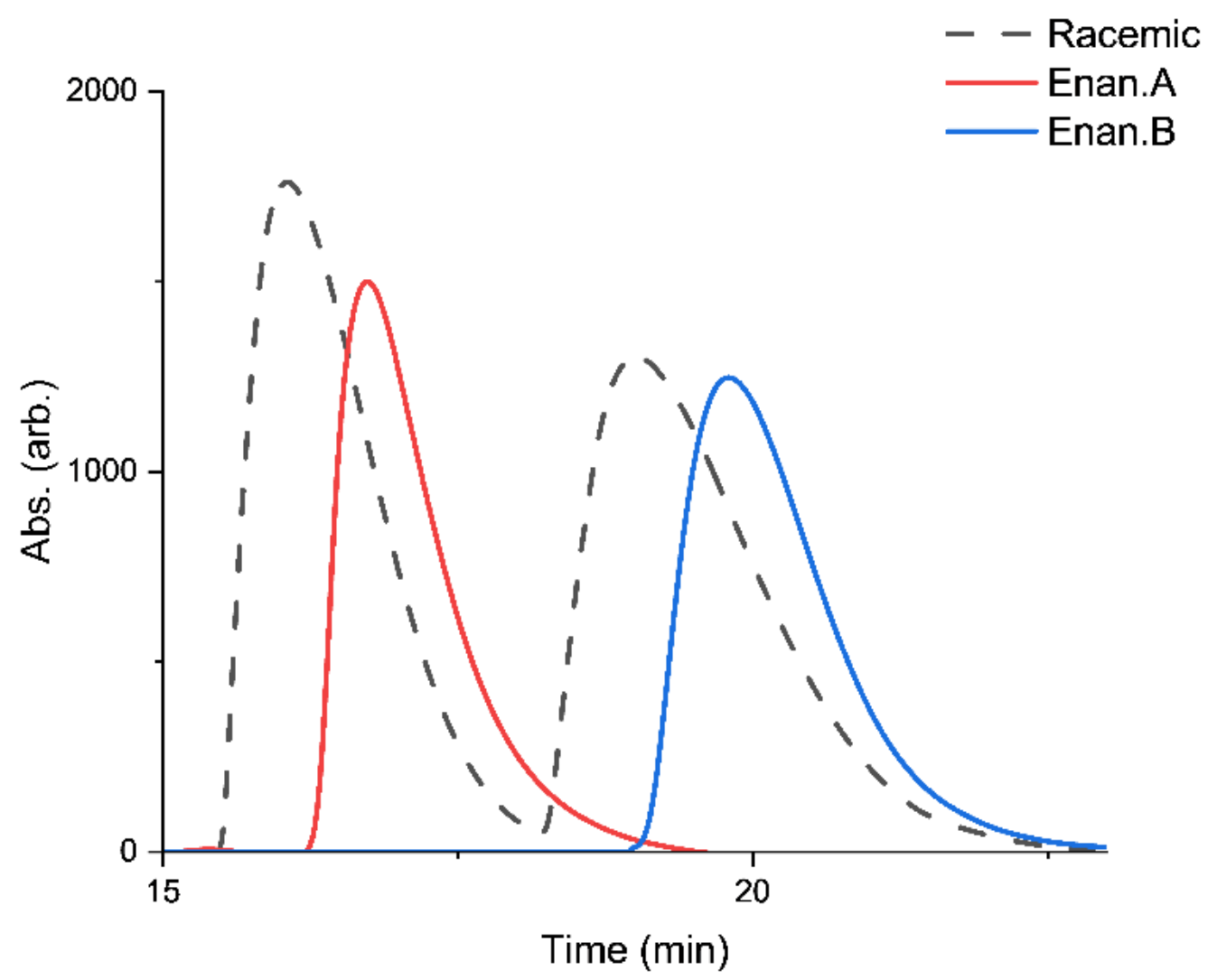

Figure S27: Chromatogram of the NDI [2]rotaxane (**1**) as a racemic mixture (dashed black trace), and reinjection of each isolated enantiomer (solid red and blue)

5) Computational Chemistry

General

Density functional theory (DFT) calculations were performed using ORCA 6.0.[7] All functionals and basis sets were chosen following the best-practice DFT protocols published by Grimme.[8] Solubilising imide groups were simplified to single methyl groups for all calculations.

Conformer searches

Conformers of NDI and PDI [2]rotaxanes **1** and **2** were predicted using a combination of the CREST[9] and the GFN2-xTB semiempirical tight-binding method.[10, 11] Structures obtained by CREST were subsequently optimised by means of DFT, using the B97-3c functional by Grimme and co-workers,[12] and the def2-TZVP basis set.[13]

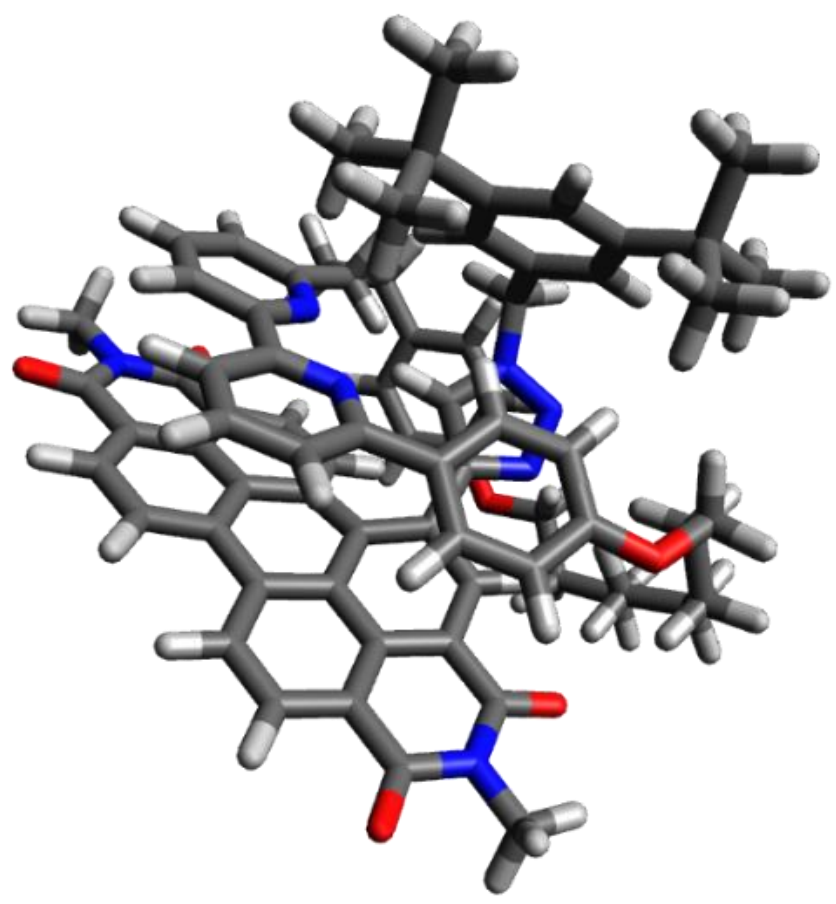

Figure S28: Lowest energy conformer of PDI [2]rotaxane (**2**) as predicted by CREST after subsequent optimisation by DFT (B97-3c/def2-TZVP).

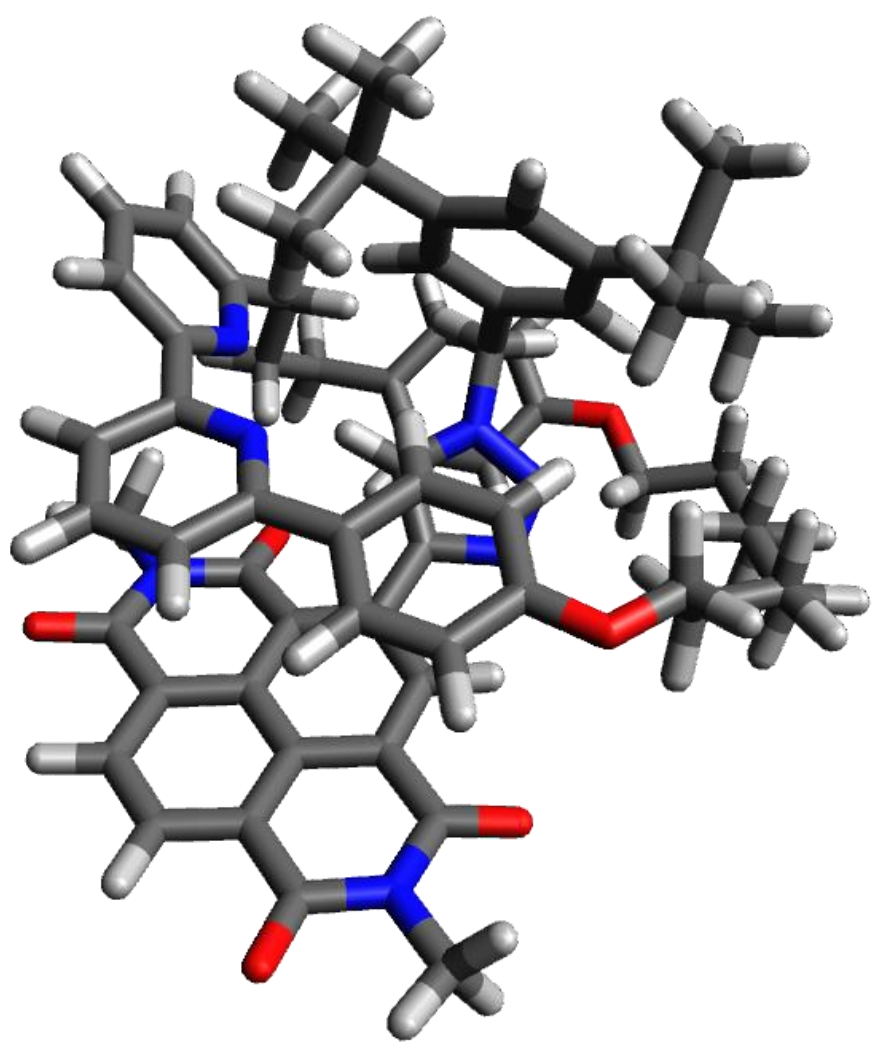

Figure S29: Lowest energy conformer of NDI [2]rotaxane (**1**) as predicted by CREST after subsequent optimisation by DFT (B97-3c/def2-TZVP).

Spectrum prediction

Vertical excitation and circular dichroism spectra were predicted using TD-DFT and were performed on the B97-3c optimised structures. Calculations for [2]rotaxane **2** were performed using the TD-ωB97x[14] functional and the def2-SVP basis-set.[7] Calculations for NDI [2]rotaxane **1** were performed using the TD-PBE0[15] functional and the def2-SVP basis-set.[7]

The chirality of both [2]rotaxanes **1** and **2** was assigned by comparing the sign of the lowest energy transition in the predicted CD spectrum of an enantiomer ($\boldsymbol{S}_{\mathbf{mp}}$) to that of the experimental CD spectrum. From this, mechanical planar chirality descriptors ($\boldsymbol{S}_{\mathbf{mp}}$/$\boldsymbol{R}_{\mathbf{mp}}$) are assigned for both [2]rotaxanes and, for PDI [2]rotaxane **2**, helical chirality labels (***M***/***P***) are also included.

Table S2: Vertical transitions calculated by TD-DFT for **2-$\boldsymbol{S}_{\mathbf{mp}}$** (ωB97x/def2-SVP).

Transition	Wavelength (nm)	f_{osc}(D2)	R (1e40*cgs)
1	493.2	0.410616	156.5997
2	358.9	0.04174	18.71574
3	330.3	0.084491	-28.3822
4	317.5	0.022222	-113.741
5	309.9	0.037945	-23.9397
6	299.7	0.000412	-0.58703
7	292.7	0.013102	-33.7722
8	284.5	0.000151	-2.45038
9	280.8	0.028953	-3.94642
10	280.1	0.102515	20.27427

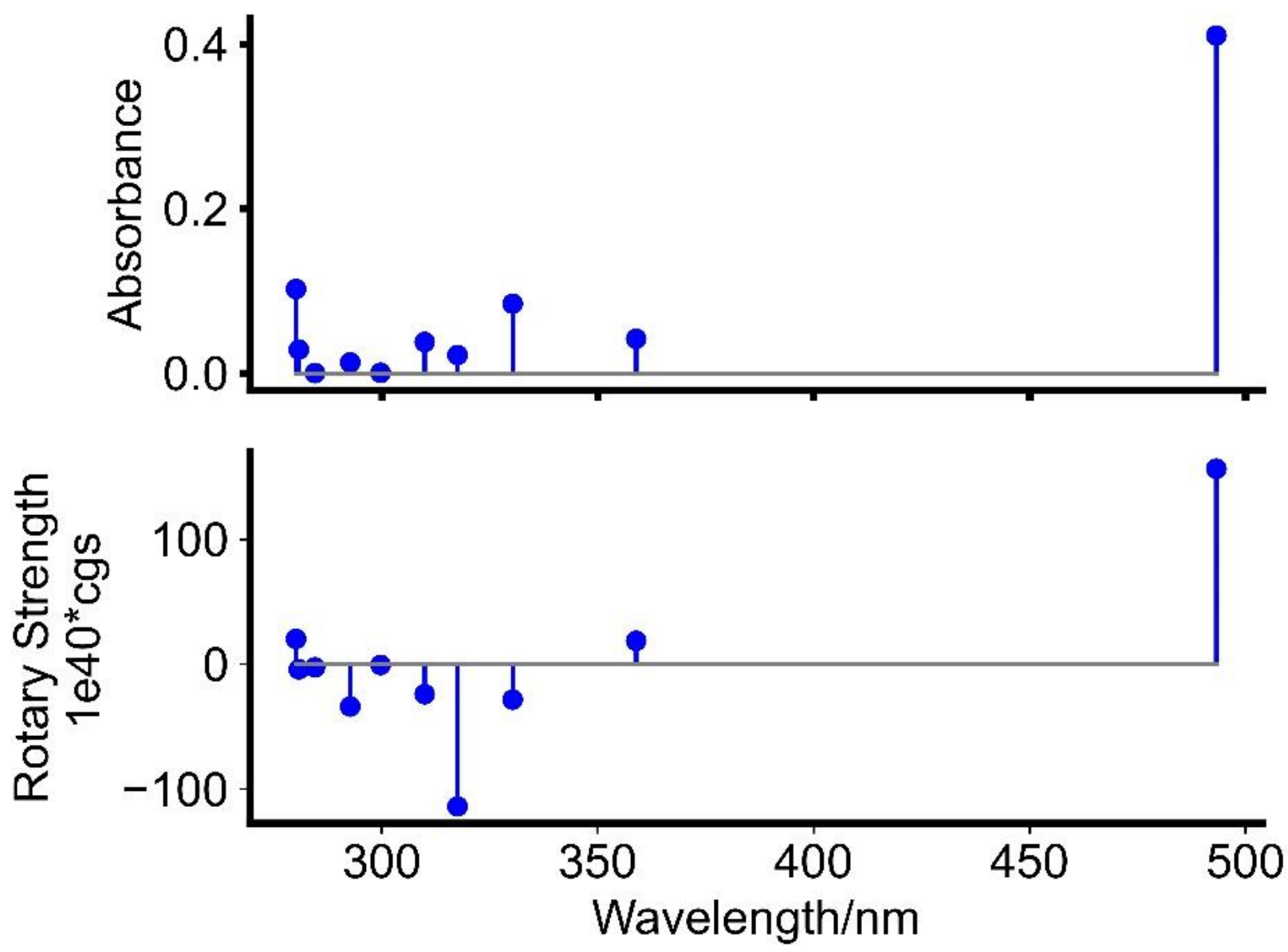

Figure S30: Calculated Absorbance and Rotatory Strength (R) for **2-$\boldsymbol{S}_{\mathbf{mp}}$**

Table S3: Vertical transitions calculated by TD-DFT for **1-*S*$_{mp}$** (PBE0/def2-SVP).

Transition	Wavelength (nm)	f_{osc}(D2)	R (1e40*cgs)
1	582	0.001897	3.03823
2	538.3	0.00727	0.33173
3	442.4	0.085379	93.18953
4	383.7	0.050521	5.81502
5	374.1	0.001714	0.65259
6	368.5	0.003963	4.8186
7	367.1	0.004134	6.23851
8	361.6	0.00054	-6.57336
9	353.8	0.055349	-22.9992
10	352.4	0.029809	9.32375
11	349.3	0.027156	-4.08728
12	342.2	0.014931	-6.9694
13	332.5	0.001662	9.2178
14	331.9	0.038695	3.08817
15	320.9	0.000753	4.09197

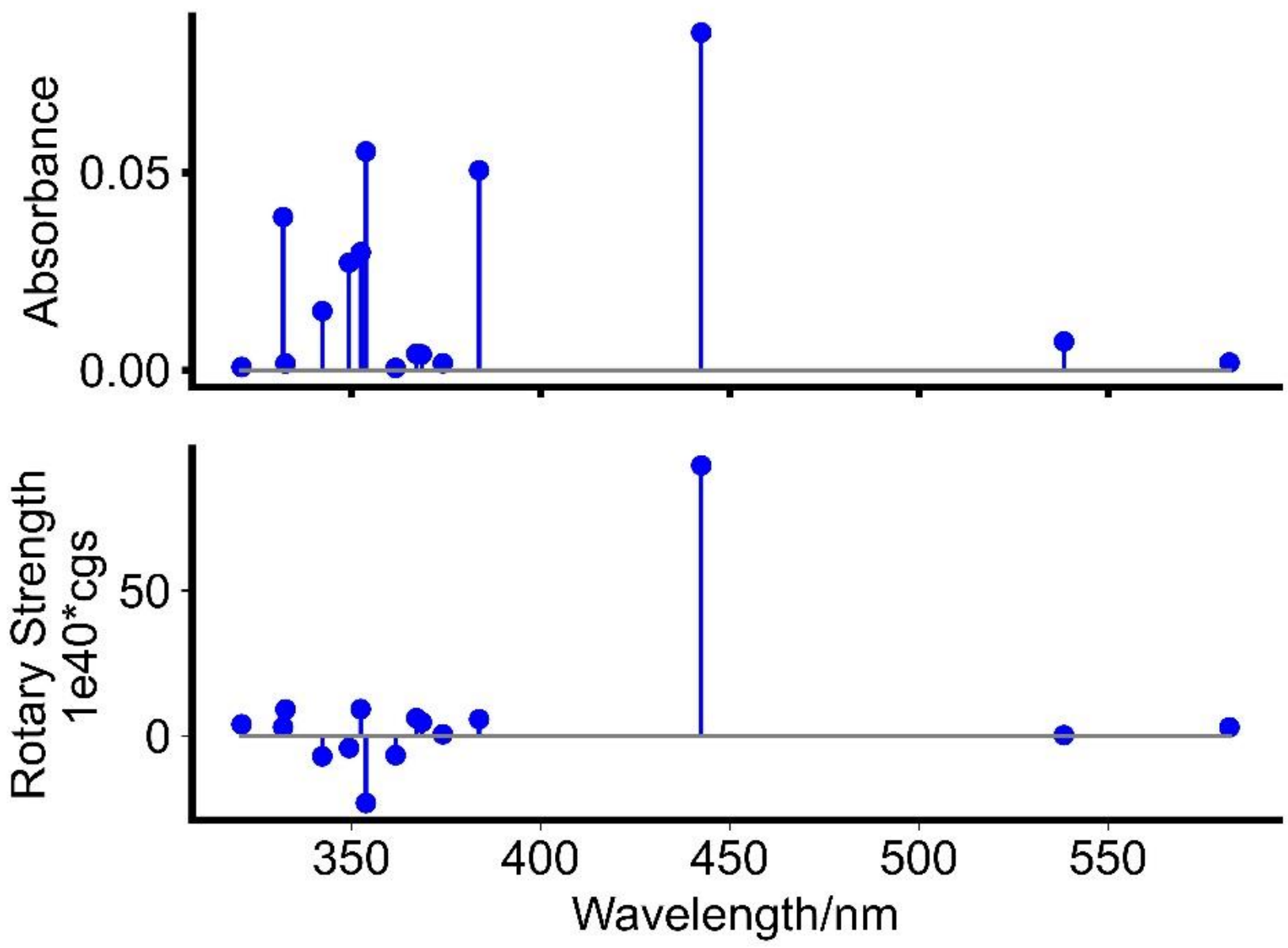

Figure S31: Calculated Absorbance and Rotatory Strength (R) for **1** (***S*$_{mp}$**).

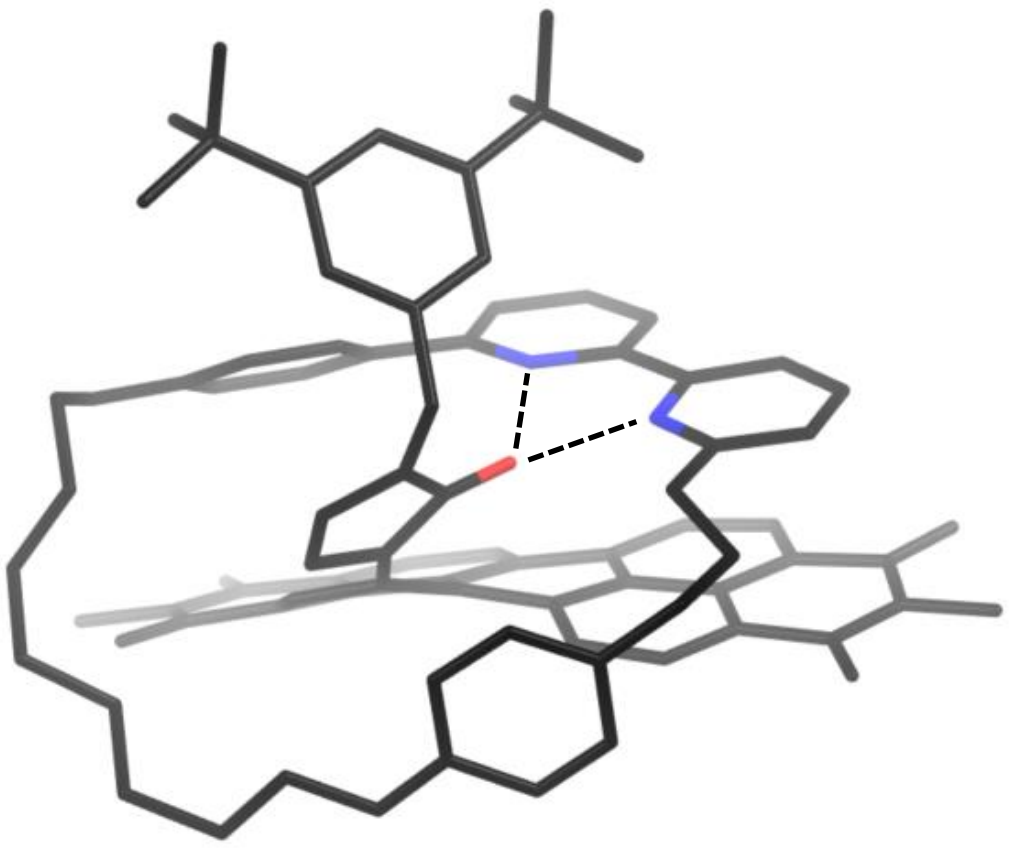

Figure S32: The calculated structure of **2** showing a hydrogen bond between triazole proton (red) and bipyridine unit (blue).

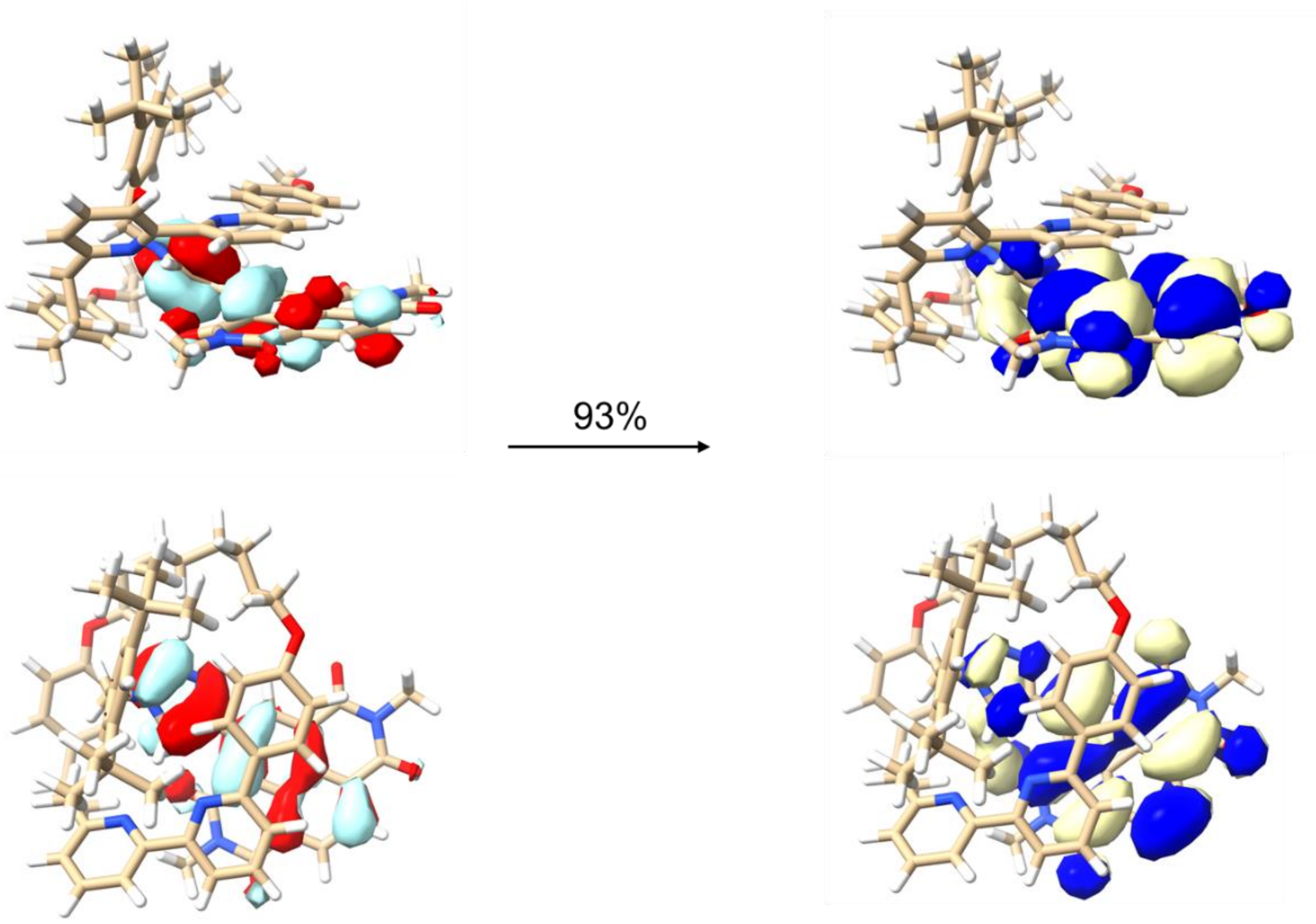

Figure S33: Orbital diagram of the major contribution (93%) to transition 3 (442 nm) for NDI rotaxane **1**, showing the (triazole → NDI) intramolecular charge transfer nature of this transition.

Rotaxane dimer structures

Structures of dimers of PDI [2]rotaxane **2** were prepared by geometric placement of the DFT-optimised structures obtained by CREST into homo- and heterochiral dimers, followed by DFT optimisation using the B97-3c functional by Grimme and co-workers,[12] and the def2-SVP basis set.[13] For the homochiral dimer (***PP***), both $\boldsymbol{M_s}$ and $\boldsymbol{P_s}$-supramolecular chirality of the two PDI units were considered and optimised. It was found that the $\boldsymbol{M_s}$-helical dimer arrangement was lower in energy by 6 kJ/mol.

For the resulting homo- and heterochiral dimer structures obtained by DFT, vertical transitions were predicted using the TD-ωB97x[14] functional and the def2-SVP basis-set.[7]

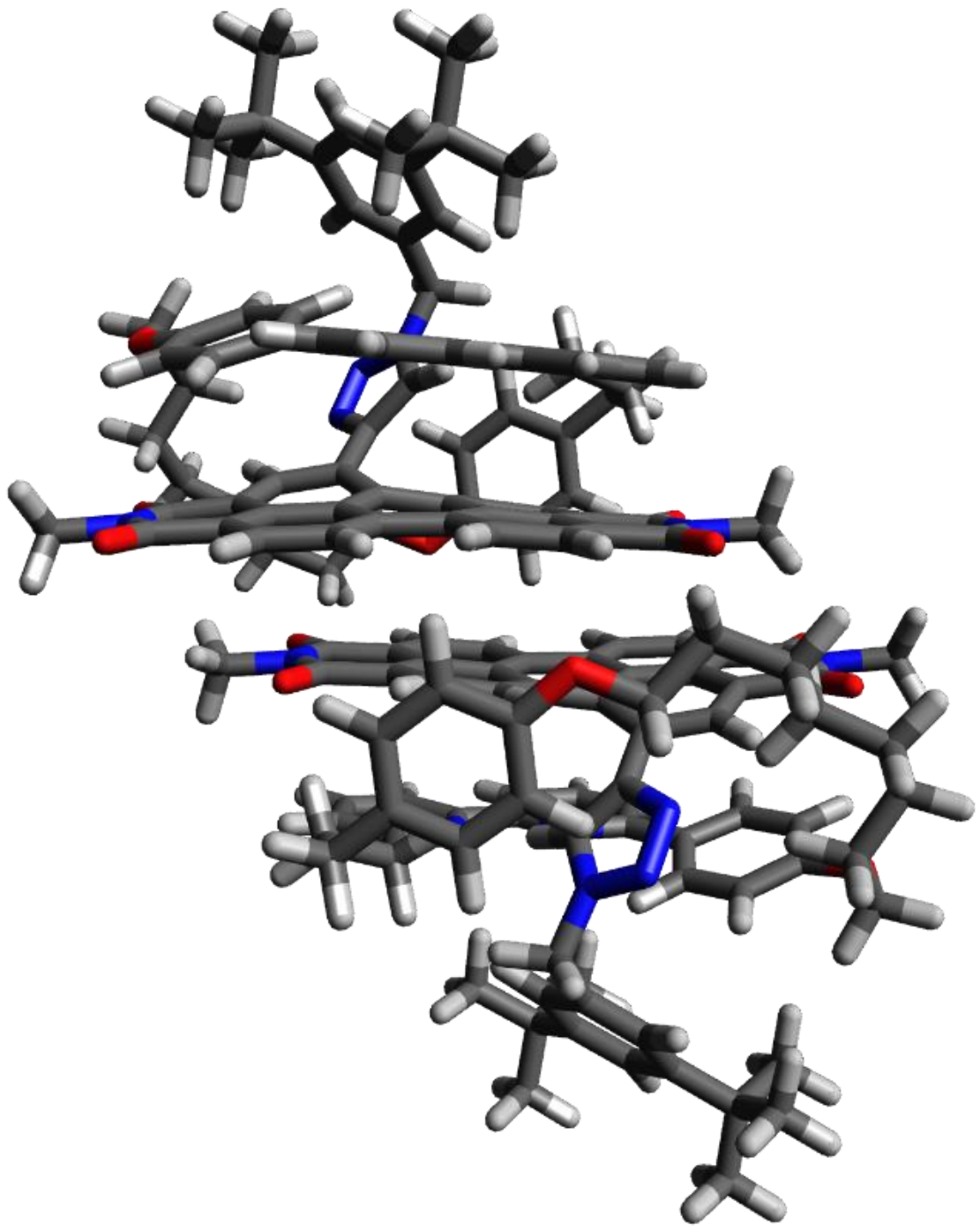

Figure S34: DFT-optimised structure of the heterochiral dimer (***MP***) of PDI [2]rotaxane **2**.

Table S4: Vertical transitions calculated by TD-DFT for the heterochiral dimer (***MP***) of PDI [2]rotaxane **2**.

Transition	Wavelength (nm)	$f_{osc}(D^2)$	R (1e40*cgs)
1	531.1	1.78E-05	1.51467
2	508.8	0.672168	-1.9767
3	400.8	7.95E-05	-0.22791
4	397.6	0.031064	1.04552
5	370.6	0.000303	-5.54526
6	369	0.029636	8.10013
7	341	0.001286	-2.30424
8	338.5	0.098178	0.85998
9	322	0.043846	-5.27167
10	321.3	3.17E-05	1.83705

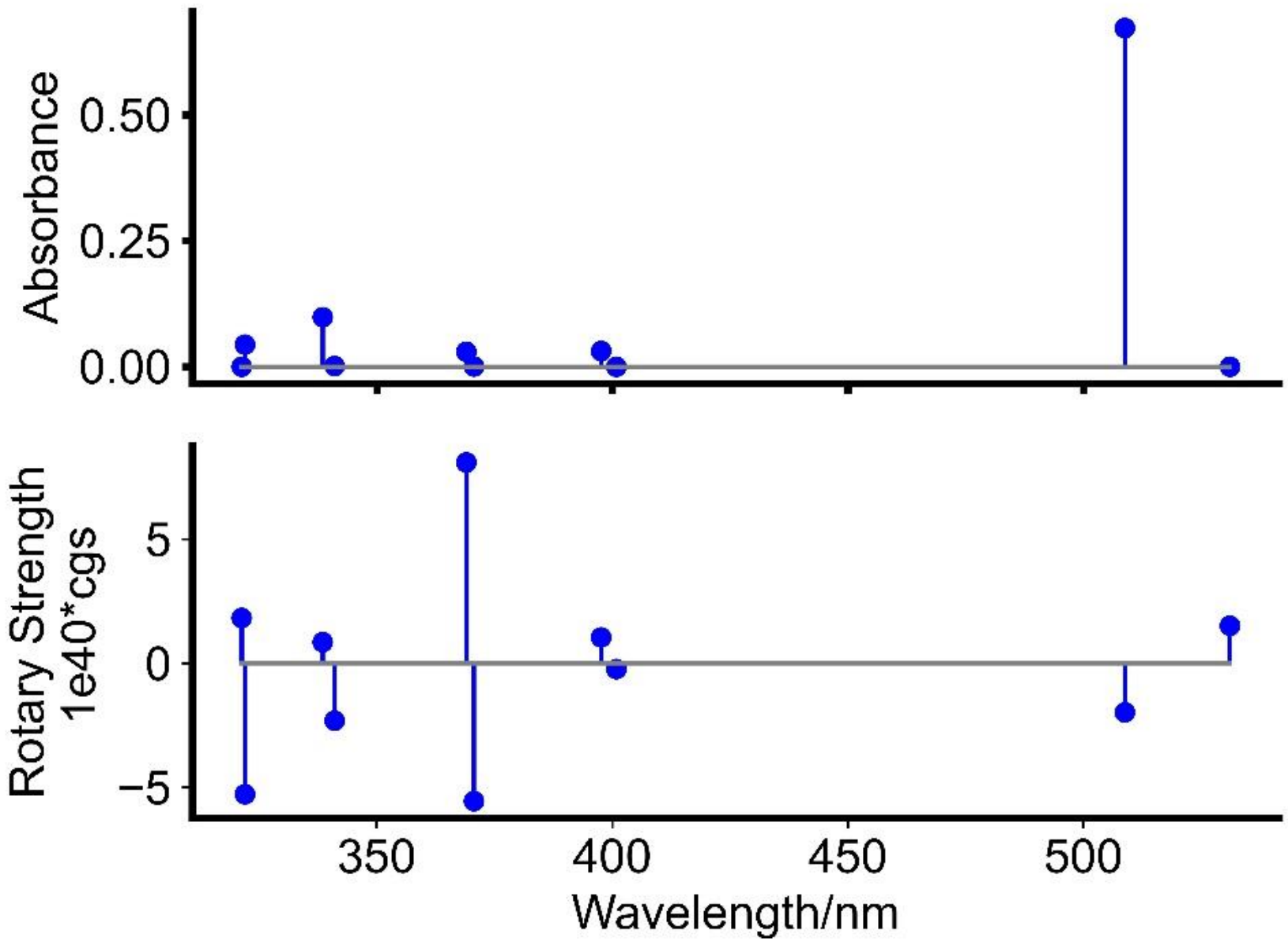

Figure S35: Calculated Absorbance and Rotatory Strength (R) for the heterochiral dimer (***MP***) of PDI [2]rotaxane **2**. Note the cancelling of CD signals throughout the spectrum, consistent with a racemate.

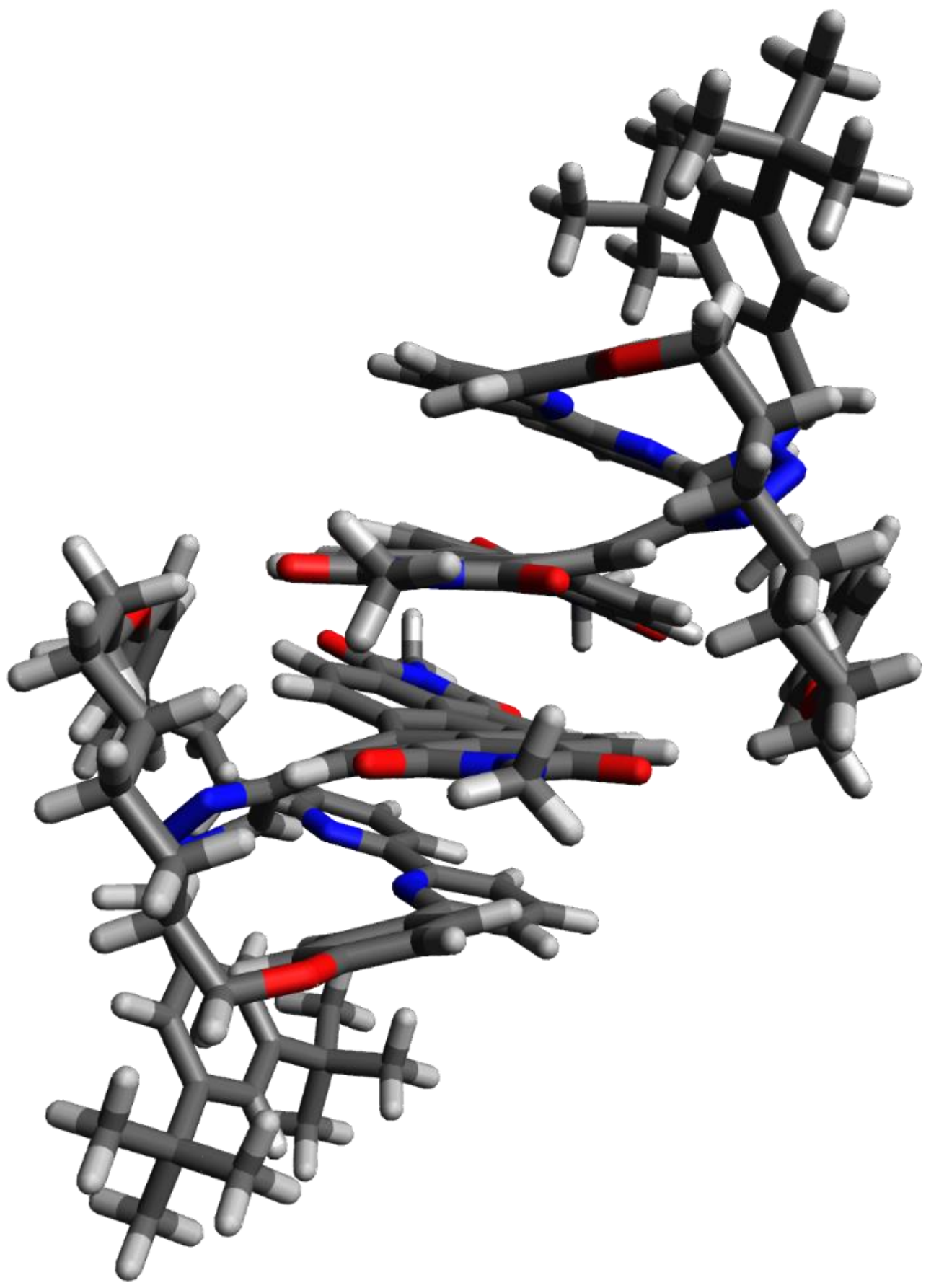

Figure S36: DFT-optimised structure of the homochiral dimer (***PP***) of PDI [2]rotaxane **2**-$\boldsymbol{S}_{\mathbf{mp}}$ with $\boldsymbol{M}_{\boldsymbol{s}}$.

Table S5: Vertical transitions calculated by TD-DFT for the homochiral dimer (***PP***) of PDI [2]rotaxane **2-*S*$_{\mathbf{mp}}$** with ***M$_s$*** supramolecular chirality. Note that the Davydov splitting, from transitions 1 and 2, is larger than for the heterochiral dimer (Table S3).

Transition	Wavelength (nm)	$f_{osc}(D^2)$	R (1e40*cgs)
1	538.7	0.011048	26.94694
2	495.6	0.47832	178.8724
3	421.3	0.020299	1.72877
4	418.3	0.016173	70.542
5	380	0.013335	38.02836
6	375.7	0.052181	57.09669
7	348.6	0.045697	102.4695
8	343.8	0.08355	-130.108
9	333.8	0.011034	21.98241
10	329.7	0.033281	-327.597

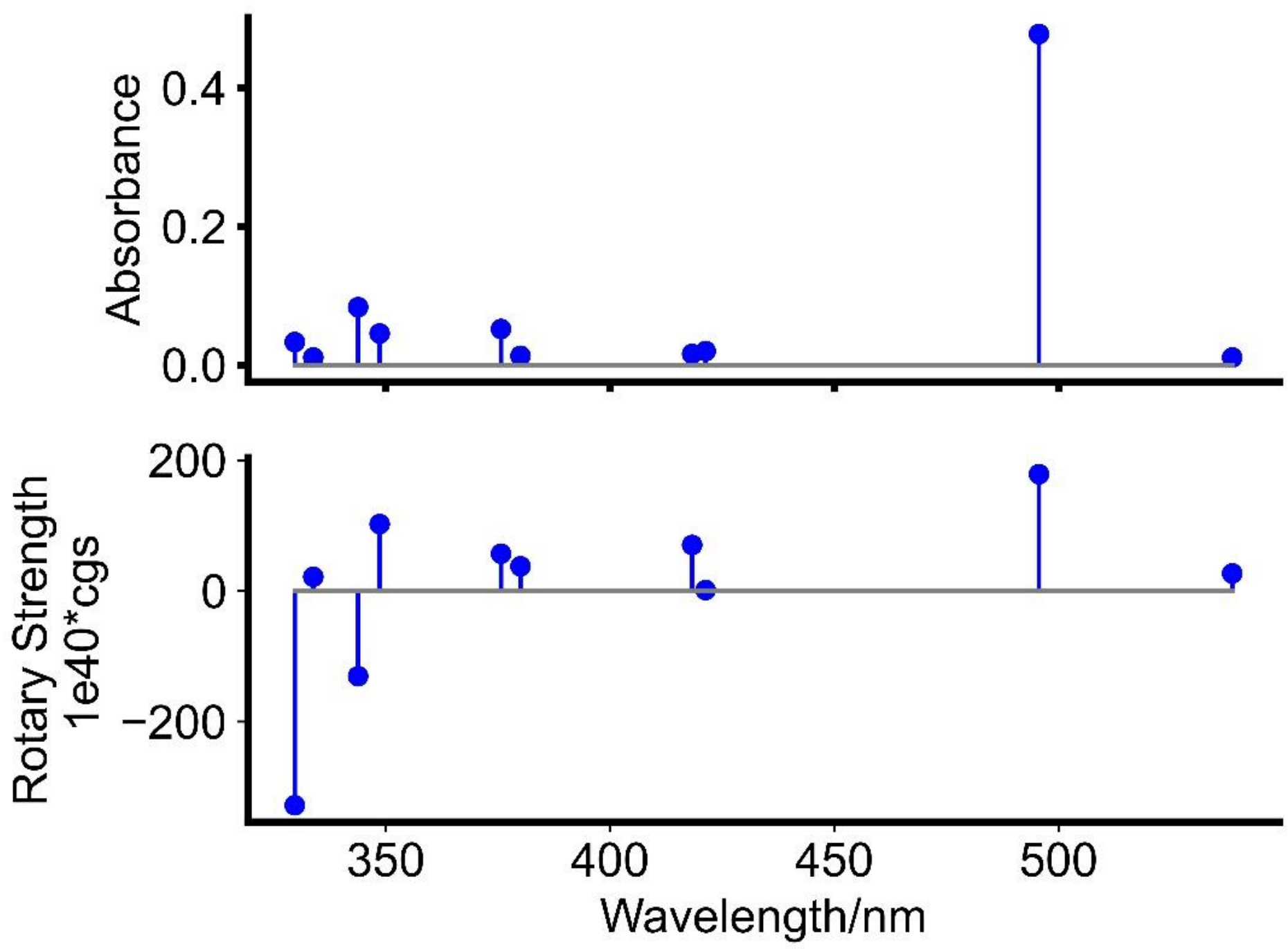

Figure S37: Calculated Absorbance and Rotatory Strength (R) for the homochiral dimer (***PP***) of PDI [2]rotaxane **2-*S*$_{\mathbf{mp}}$** with ***M$_s$*** supramolecular chirality.

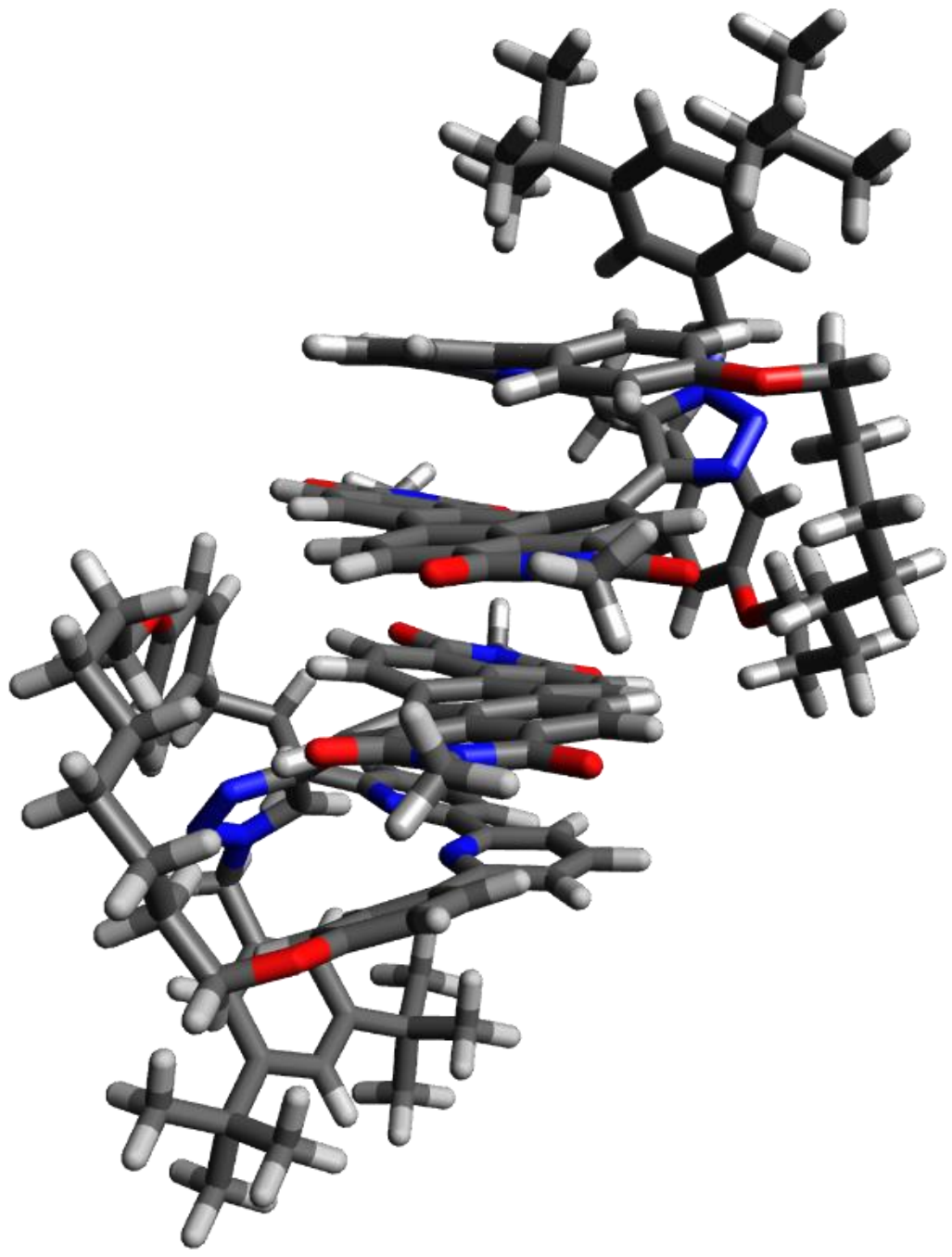

Figure S38: DFT-optimised structure of the homochiral dimer (***PP***) of PDI [2]rotaxane **2-*S*$_{mp}$** with ***P$_s$***. This structure was found to be 6 kJ/mol higher in energy at the chosen level of theory (B97-3c/def2-SVP) than the corresponding ***M$_s$***-chiral structure.

Table S6: Vertical transitions calculated by TD-DFT for the higher energy homochiral dimer (***PP***) of PDI [2]rotaxane **2-*S*$_{mp}$** with ***P$_s$*** supramolecular chirality.

Transition	Wavelength (nm)	$f_{osc}(D^2)$	R (1e40*cgs)
1	535.6	0.06668	348.5466
2	496.9	0.438912	-70.2369
3	420.3	0.006887	-18.5647
4	414.3	0.033734	37.72653
5	374.6	0.026214	12.97931
6	367.5	0.014803	-12.7088
7	345.6	0.087407	17.46398
8	342	0.068275	13.35041
9	328.1	0.02523	-203.646
10	323.5	0.024525	-14.3922

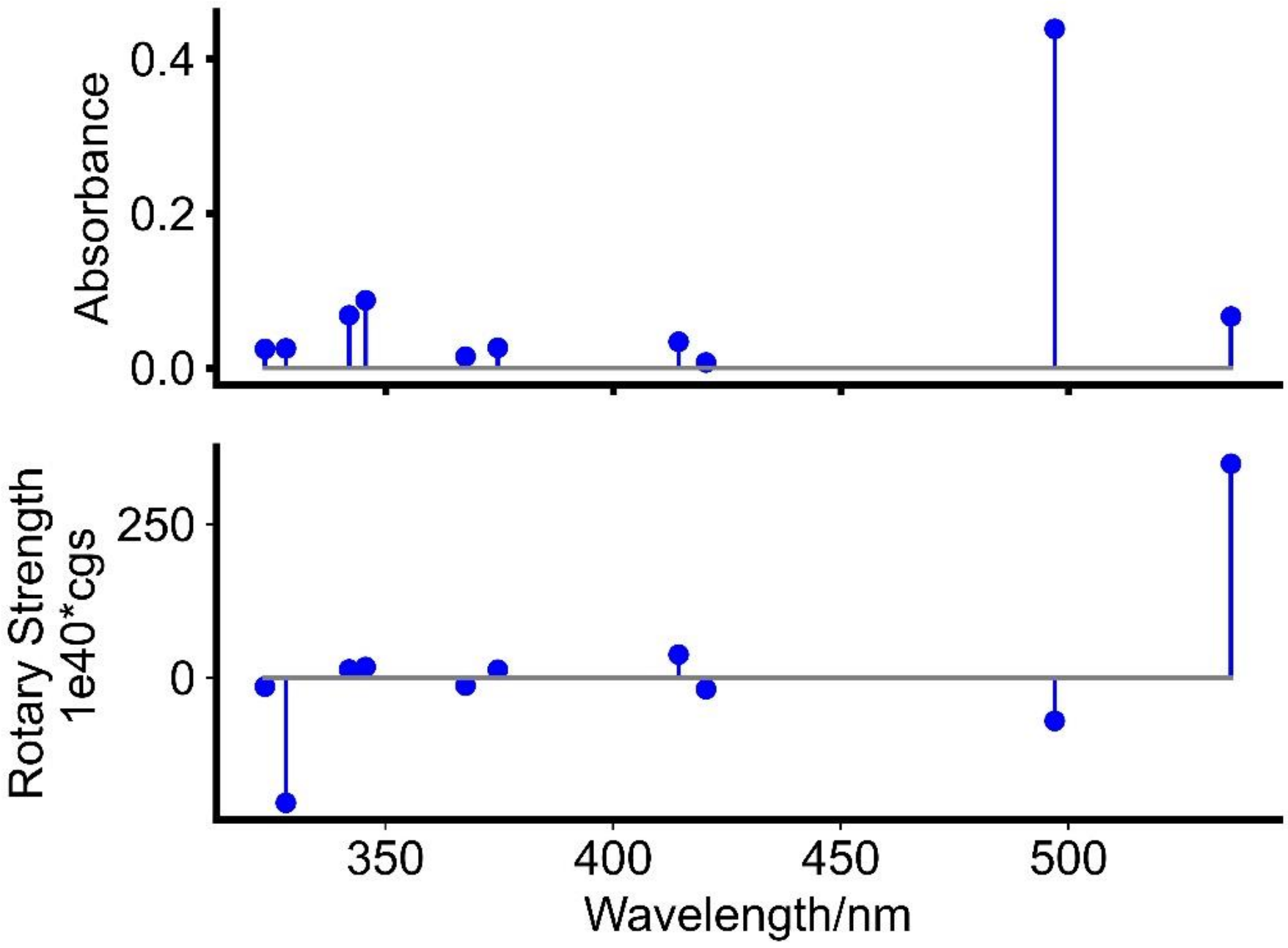

Figure S39: Calculated Absorbance and Rotatory Strength (R) for the higher energy homochiral dimer (***PP***) of PDI [2]rotaxane **2-*S*$_{mp}$** with ***P$_s$*** supramolecular chirality.

6) NMR Spectra

PDI [2]rotaxane (2)

^{1}H NMR (400 MHz, TCE, 373 K)

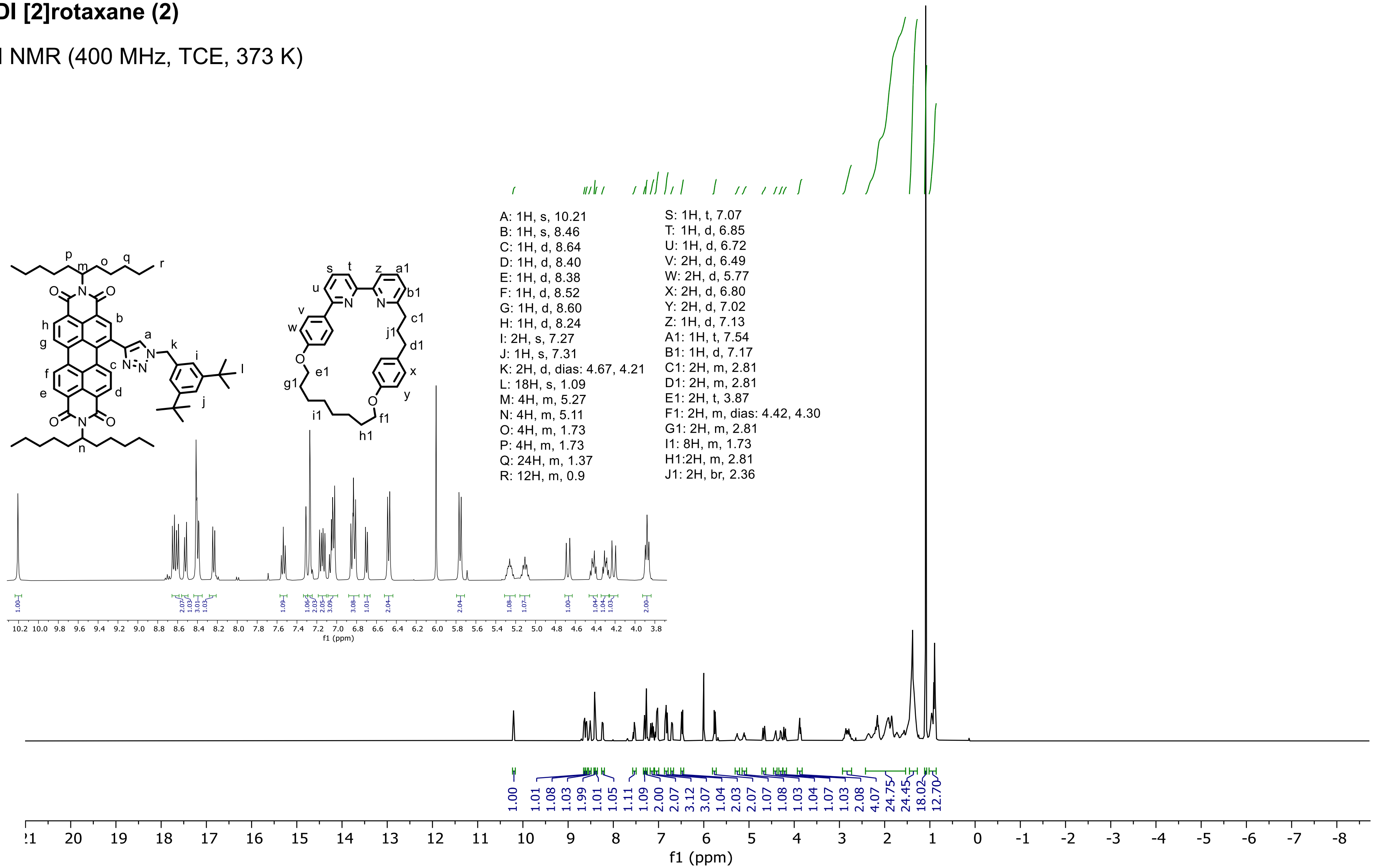

^{13}C NMR (400 MHz, TCE, 373 K)

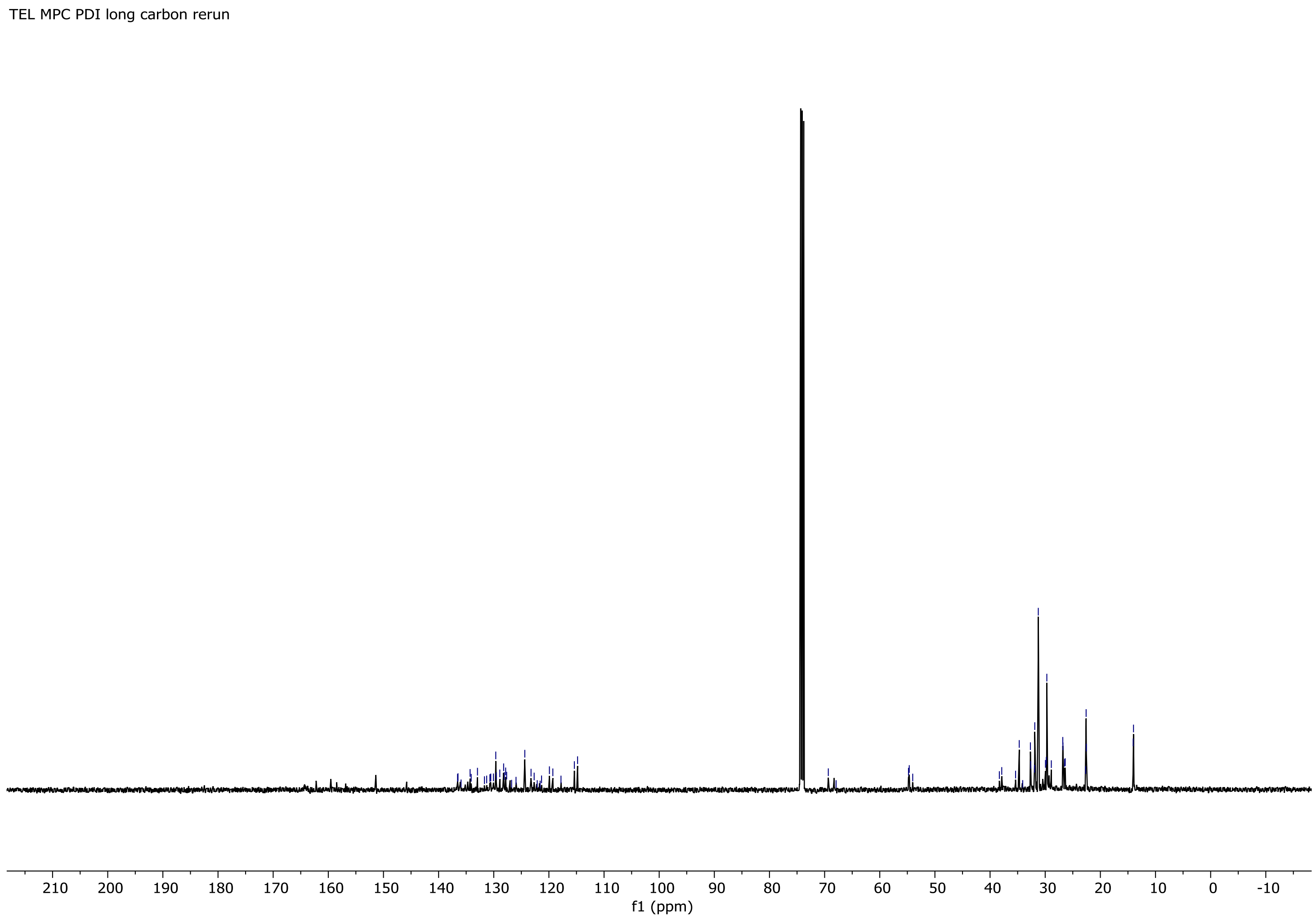

MonoTriazole-C11-PDI (g)

^{1}H NMR (400 MHz, $CDCl_3$)

TEL MonoTrz PDI 2D
1D1H.icon CDCl3 {C:\DATA\TBARENDT\2025} TABarendt 7

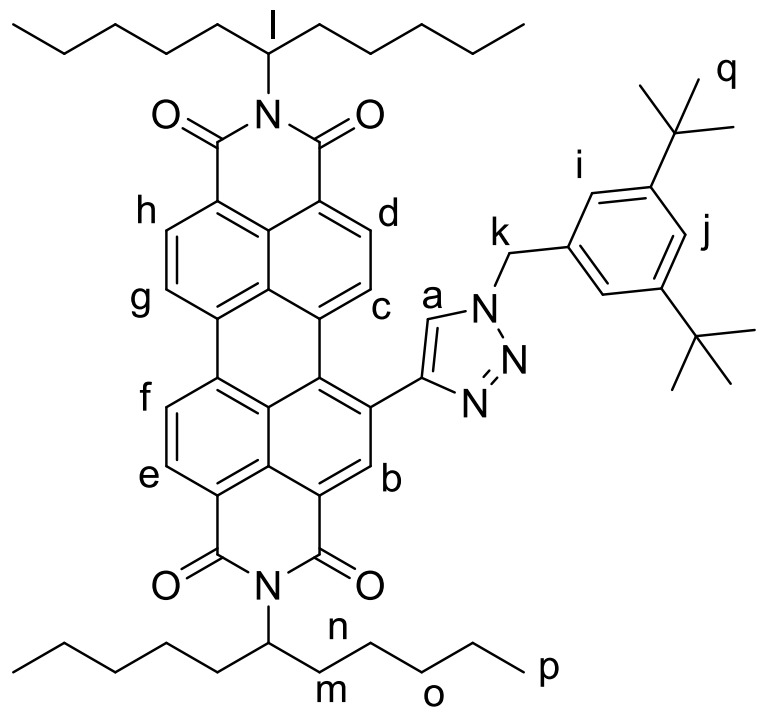

a: 1H, s, 7.68
b: 1H, br, 8.53
c: 1H, d, 7.95
d: 1H, br, 8.19
e-h: 4H, m, 8.56-8.71
i: 2H, d, 7.23
j: 1H, s, 7.47
k: 2H, s, 5.66
l: 2H, m, 5.16
m: 4H, m, 2.22
n: 4H, m, 1.82
o: 24H, m ,1.26
p: 12H, m ,0.83
q: 18H, s, 1.34

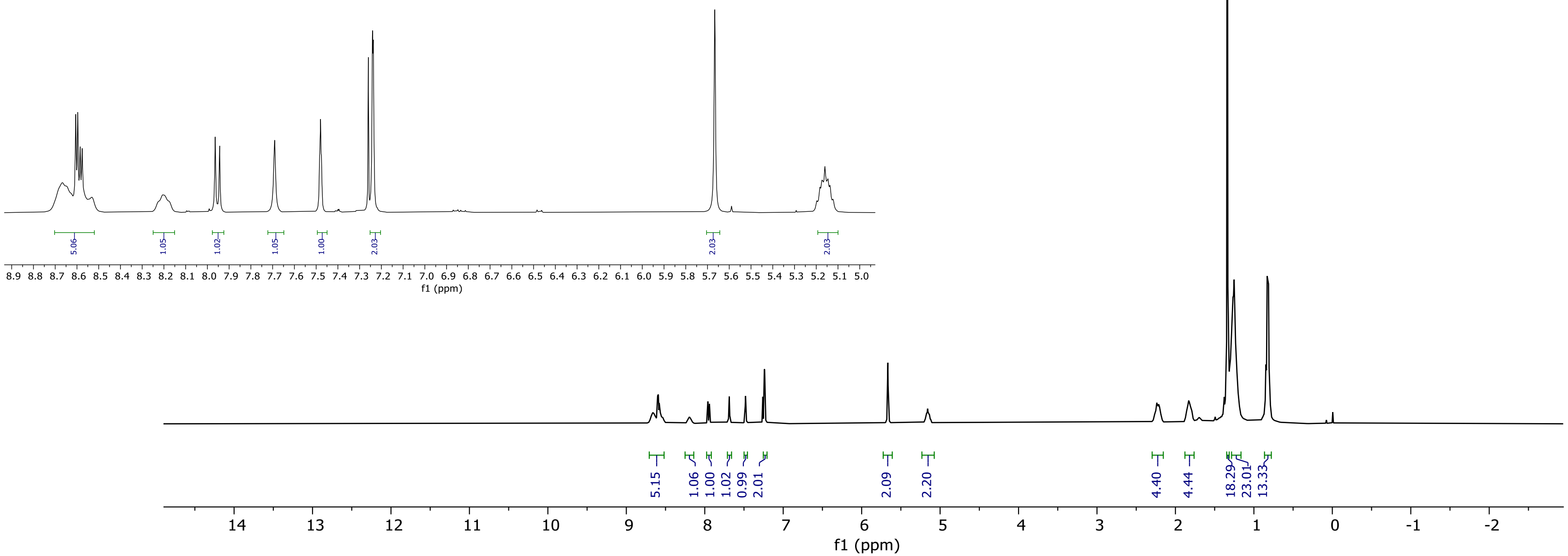

^{13}C NMR (400 MHz, $CDCl_3$)

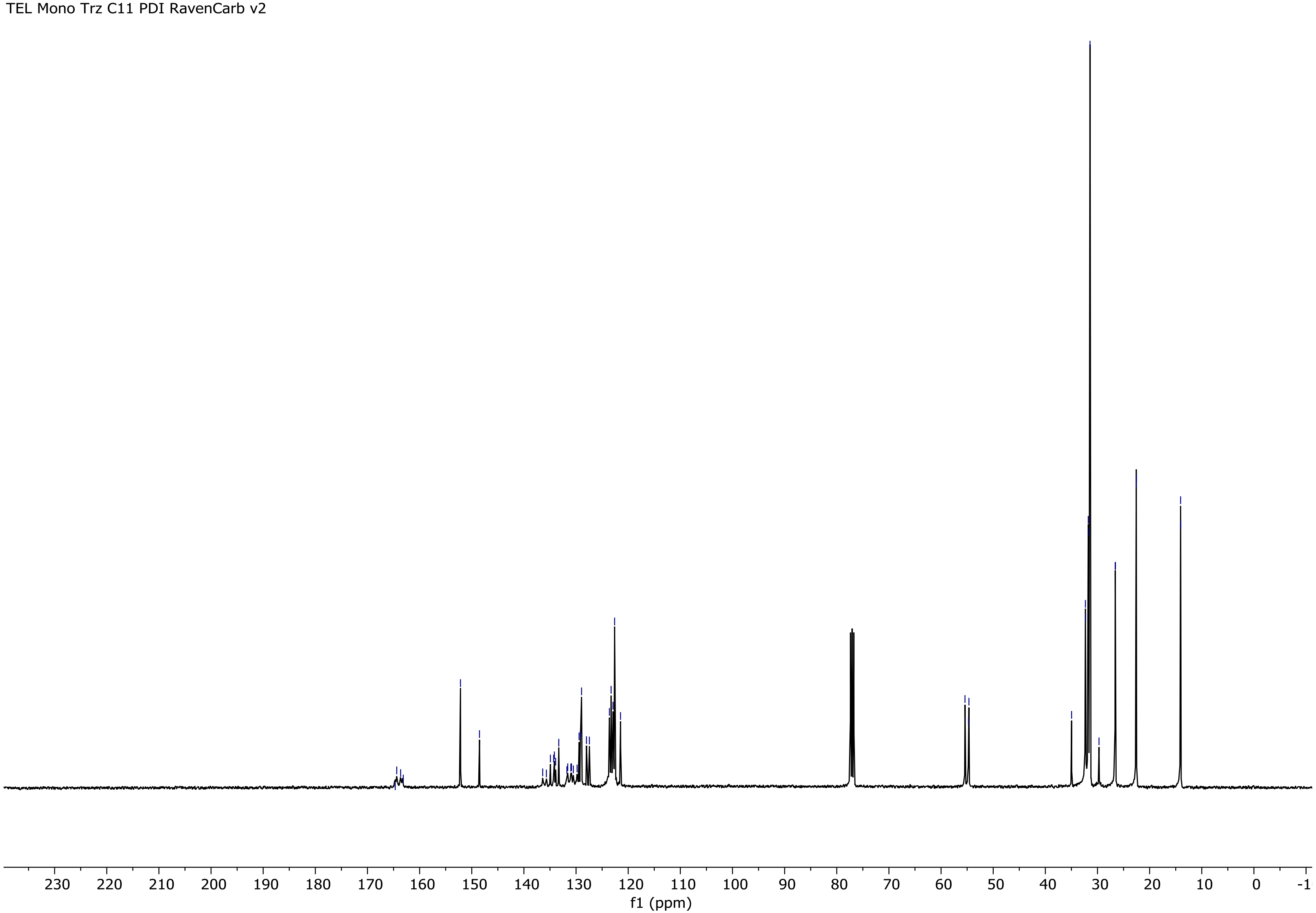

MonoBr-C5-NDI (j)

^{1}H NMR (400 MHz, $CDCl_3$)

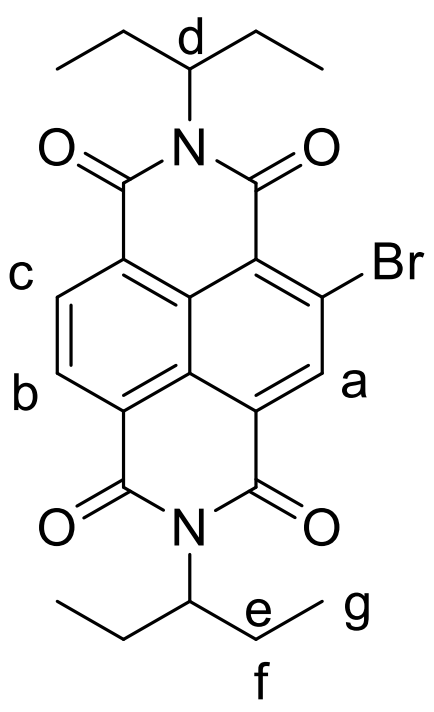

a: 1H, s, 8.90
b-c: 2H, m, 8.82-8.69
d: 2H, m, 5.09
e: 4H, m, 2.19
f: 4H, m, 2.00-1.89
g: 12H, m, 0.87

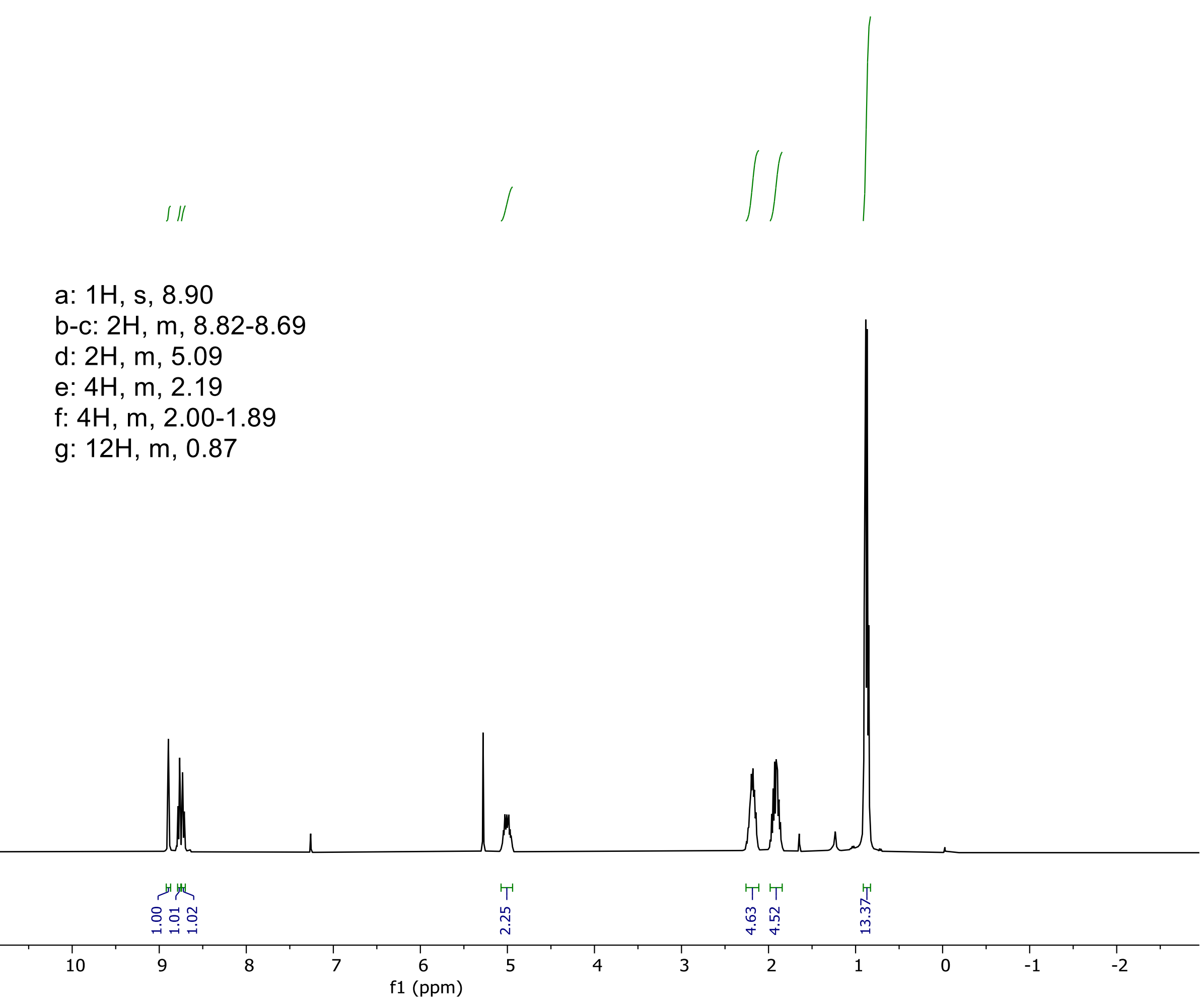

^{13}C NMR (400 MHz, $CDCl_3$)

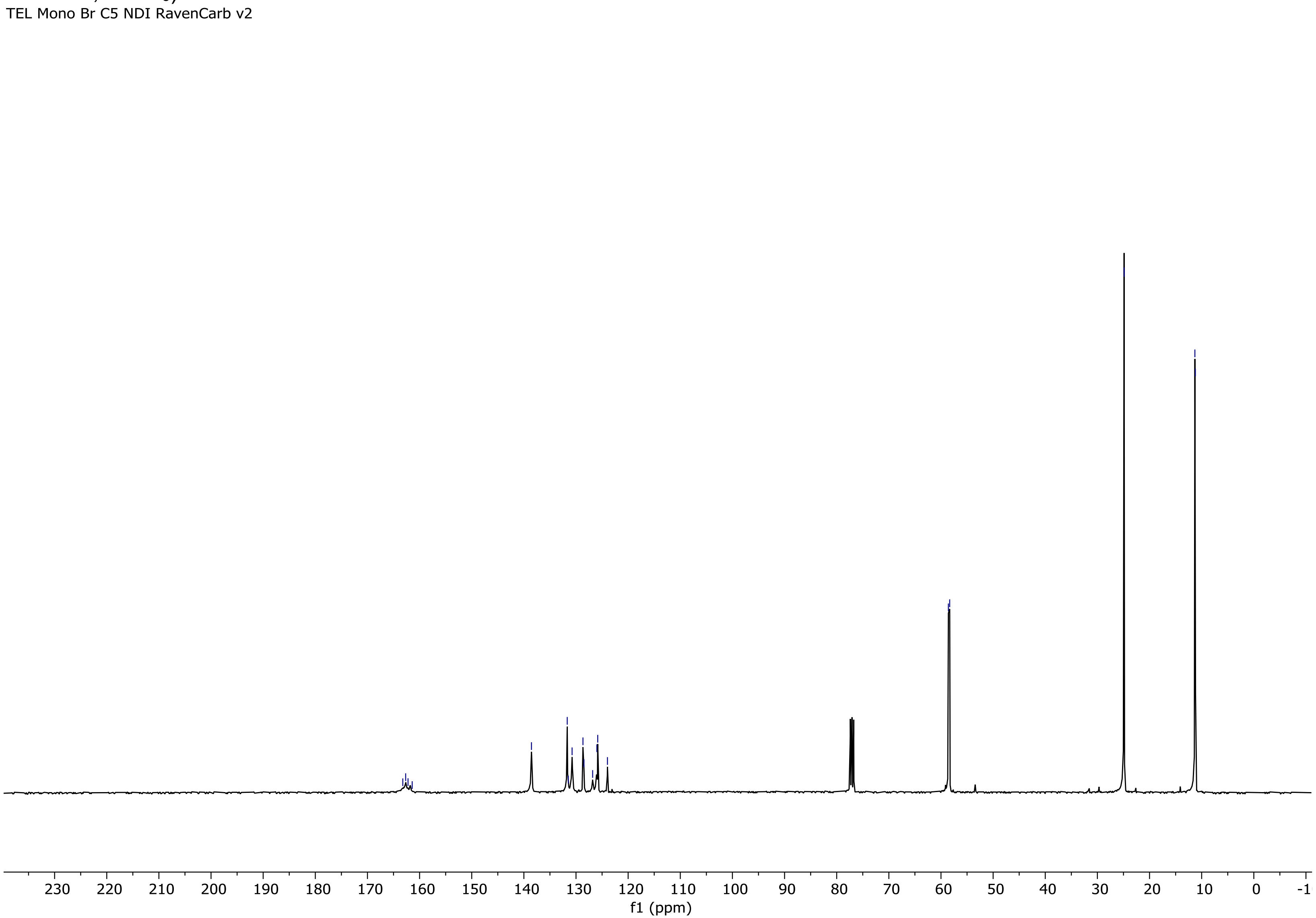

MonoTIPSAlkynyl-C5-NDI (k)

^{1}H NMR (400 MHz, $CDCl_3$)

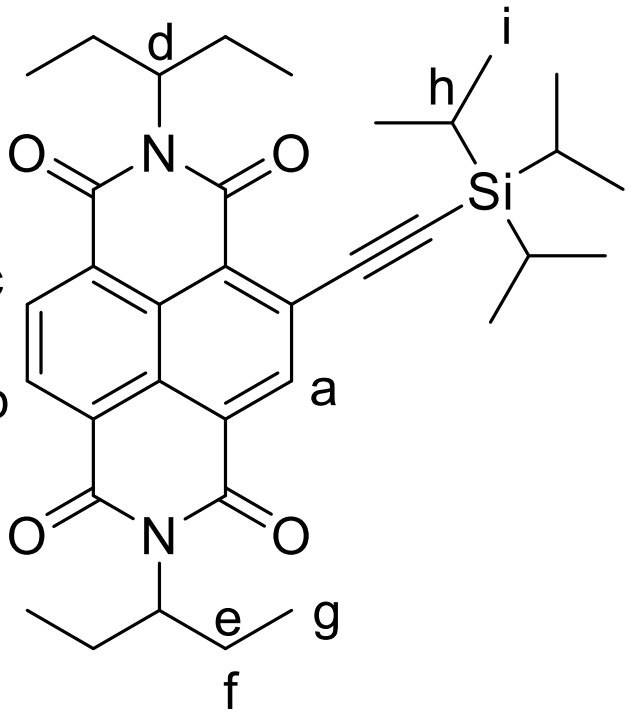

a-c: 3H, m, 8.75-8.63
d: 2H, m, 4.99
e: 4H, m, 2.19
f: 4H, m, 1.88
g: 12H, m, 0.86
h-i: 21H, m, 1.28-1.11

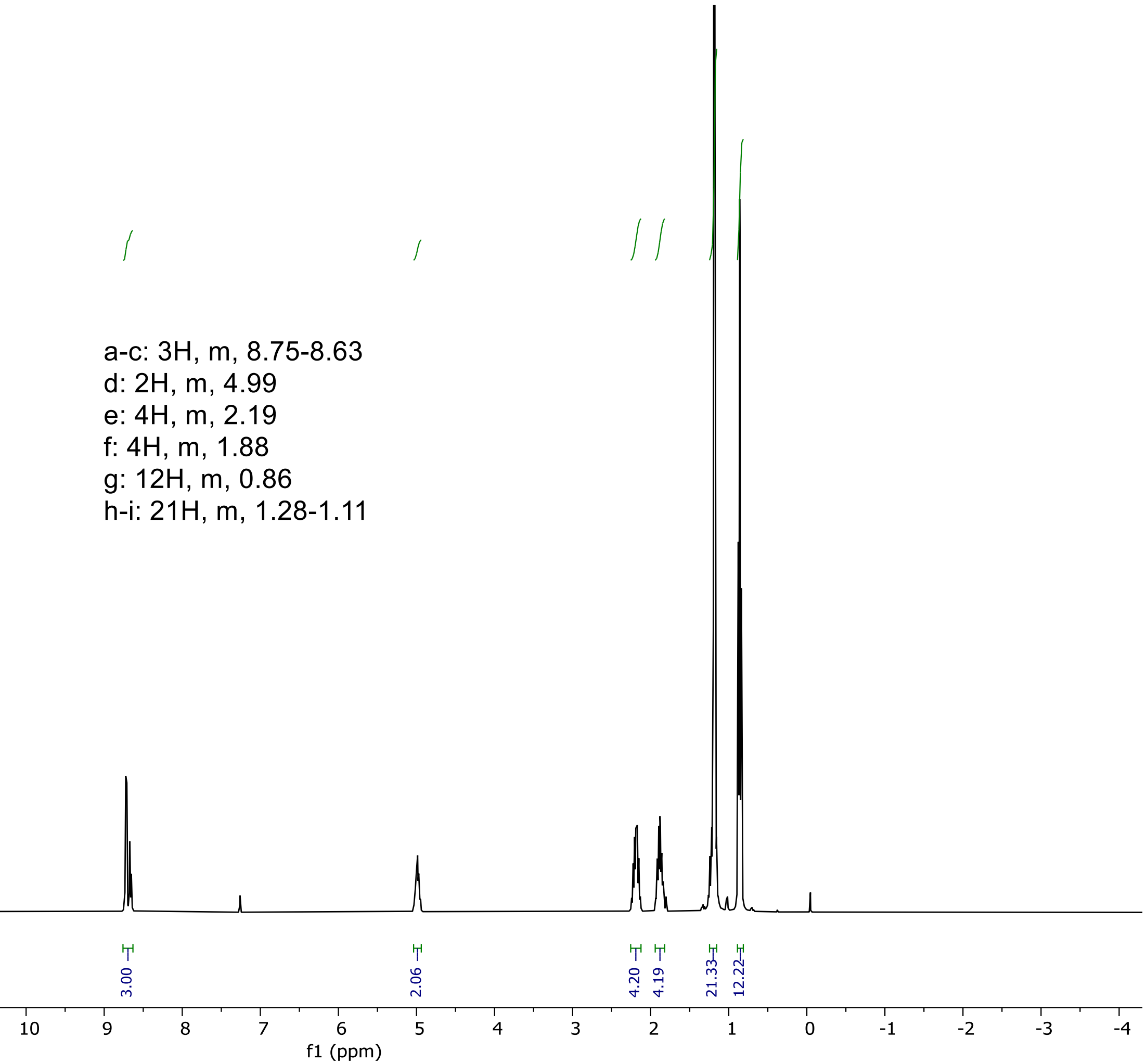

^{13}C NMR (400 MHz, $CDCl_3$)

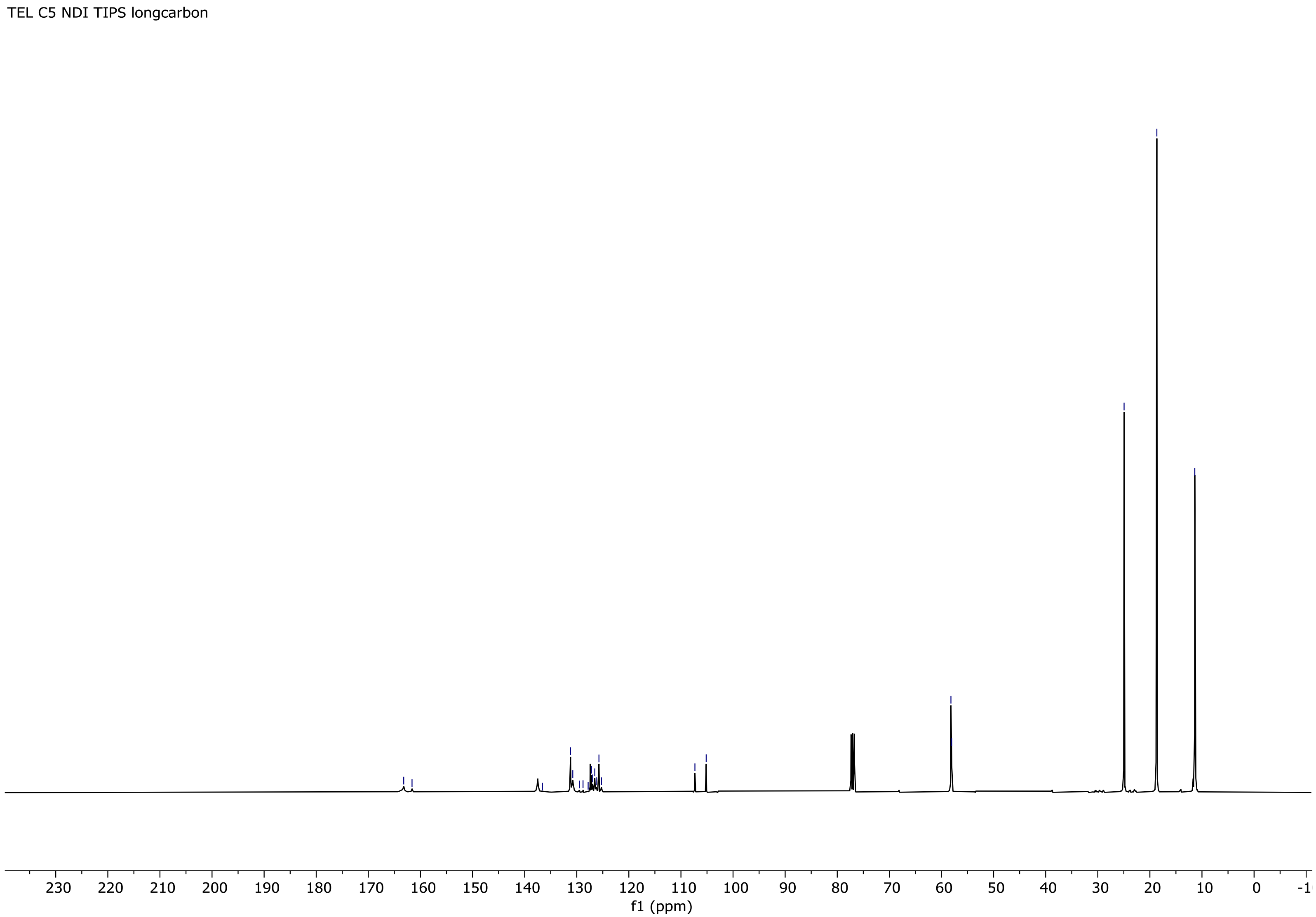

NDI [2]Rotaxane (1)

^{1}H NMR (400 MHz, TCE, 373 K)

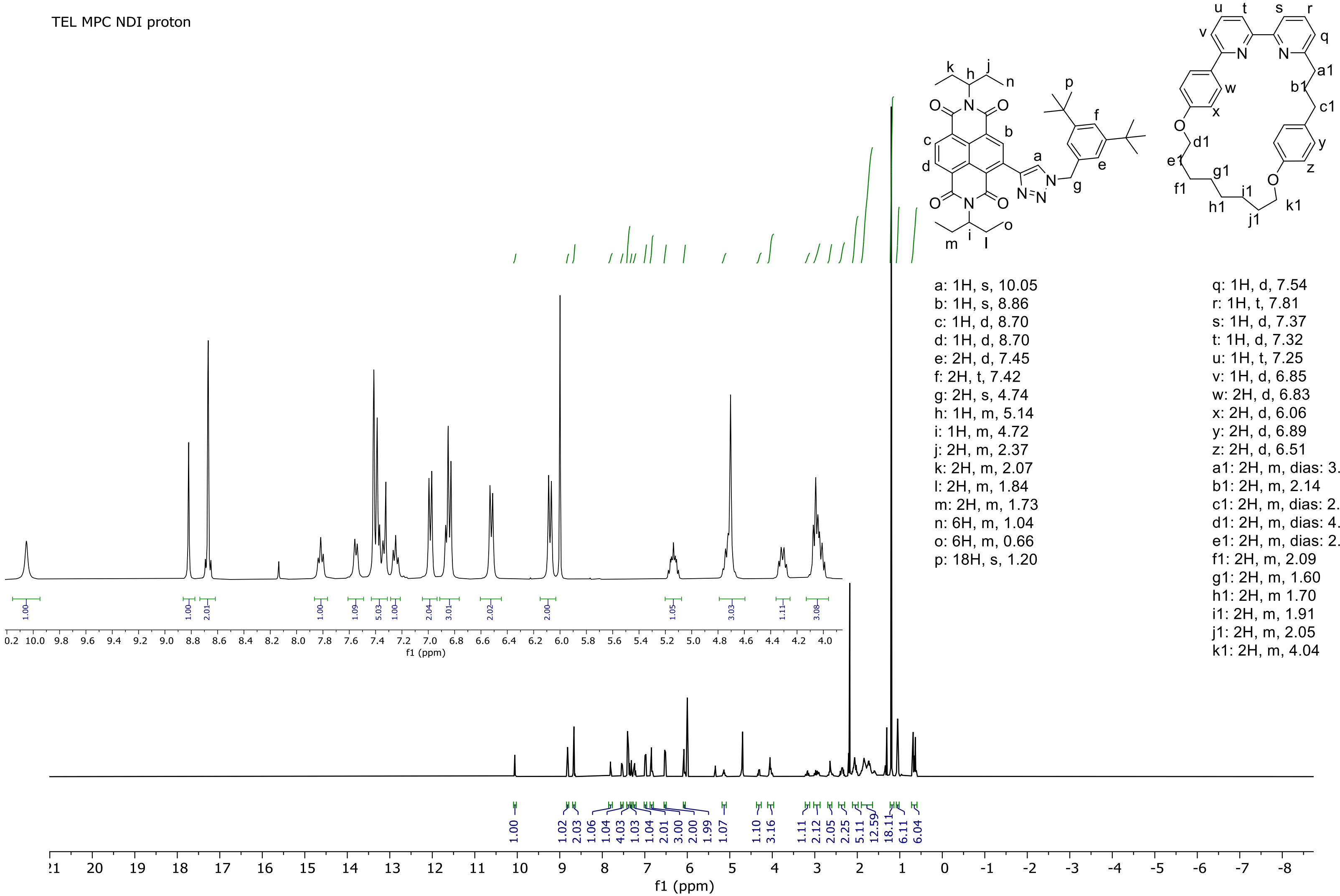

a: 1H, s, 10.05
b: 1H, s, 8.86
c: 1H, d, 8.70
d: 1H, d, 8.70
e: 2H, d, 7.45
f: 2H, t, 7.42
g: 2H, s, 4.74
h: 1H, m, 5.14
i: 1H, m, 4.72
j: 2H, m, 2.37
k: 2H, m, 2.07
l: 2H, m, 1.84
m: 2H, m, 1.73
n: 6H, m, 1.04
o: 6H, m, 0.66
p: 18H, s, 1.20

q: 1H, d, 7.54
r: 1H, t, 7.81
s: 1H, d, 7.37
t: 1H, d, 7.32
u: 1H, t, 7.25
v: 1H, d, 6.85
w: 2H, d, 6.83
x: 2H, d, 6.06
y: 2H, d, 6.89
z: 2H, d, 6.51
a1: 2H, m, dias: 3.17, 2.97
b1: 2H, m, 2.14
c1: 2H, m, dias: 2.93, 2.65
d1: 2H, m, dias: 4.35, 4.06
e1: 2H, m, dias: 2.06, 1.90
f1: 2H, m, 2.09
g1: 2H, m, 1.60
h1: 2H, m 1.70
i1: 2H, m, 1.91
j1: 2H, m, 2.05
k1: 2H, m, 4.04

^{13}C NMR (400 MHz, TCE, 373 K)

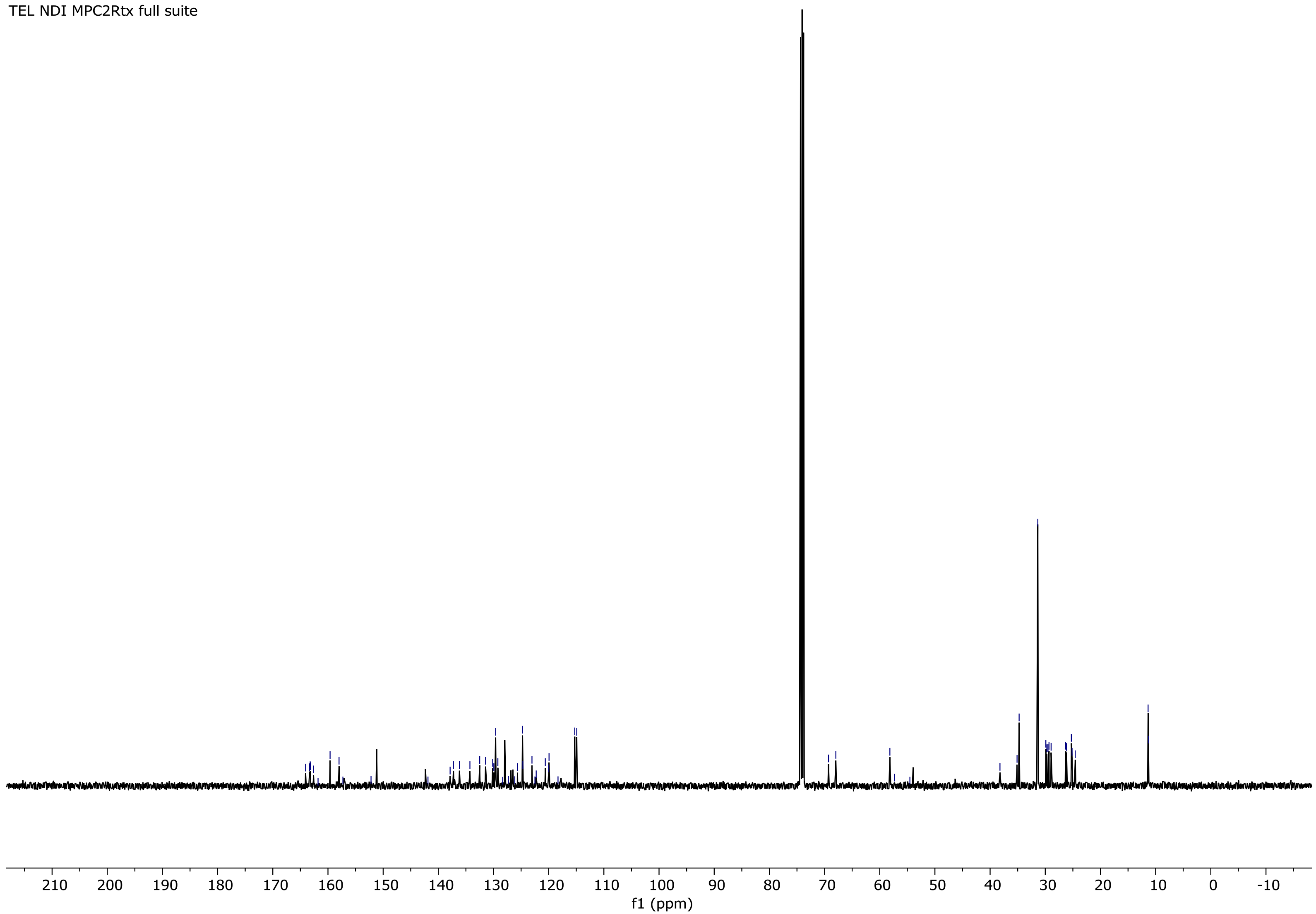

MonoTriazole-C5-NDI (I)

^{1}H NMR (400 MHz, $CDCl_3$)

TEL C5 NDI Click full

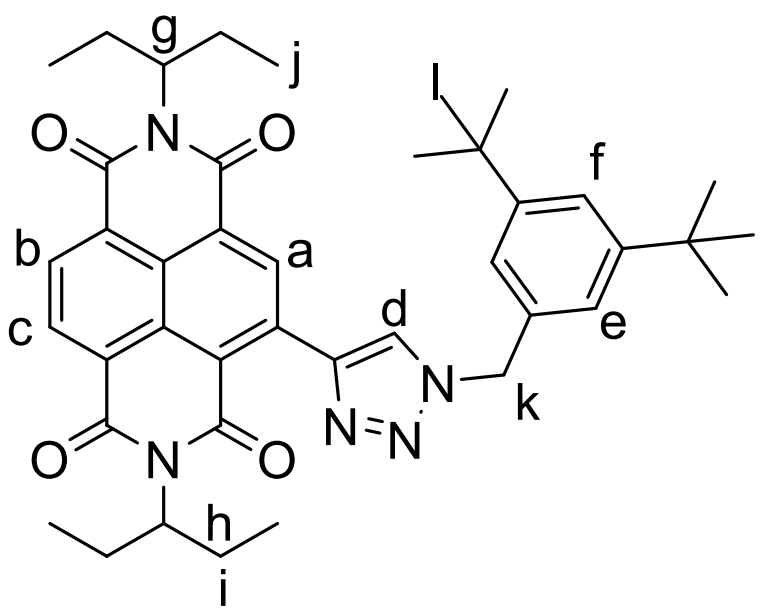

a: 1H, s, 9.34
b: 1H, d, 8.76
c: 1H, d, 8.70
d: 1H, s, 8.34
e: 1H, t, 7.45
f: 2H, d, 7.24
g: 2H, m, 5.09-4.89
h: 4H, m, 2.30-2.06
i: 4H, m, 1.99-.181
j: 12H, m, 0.87
k: 2H, s, 5.67
l: 18H, s, 1.33

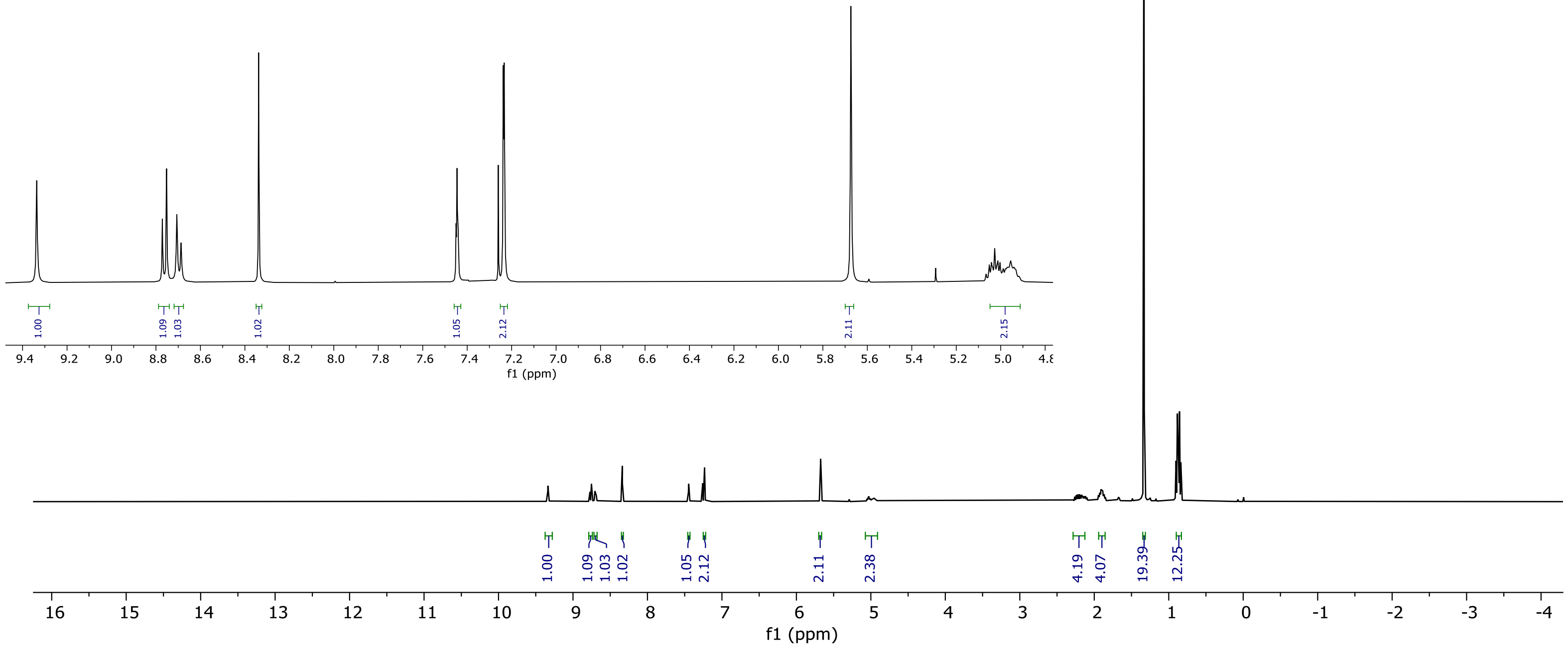

^{13}C NMR (400 MHz, $CDCl_3$)

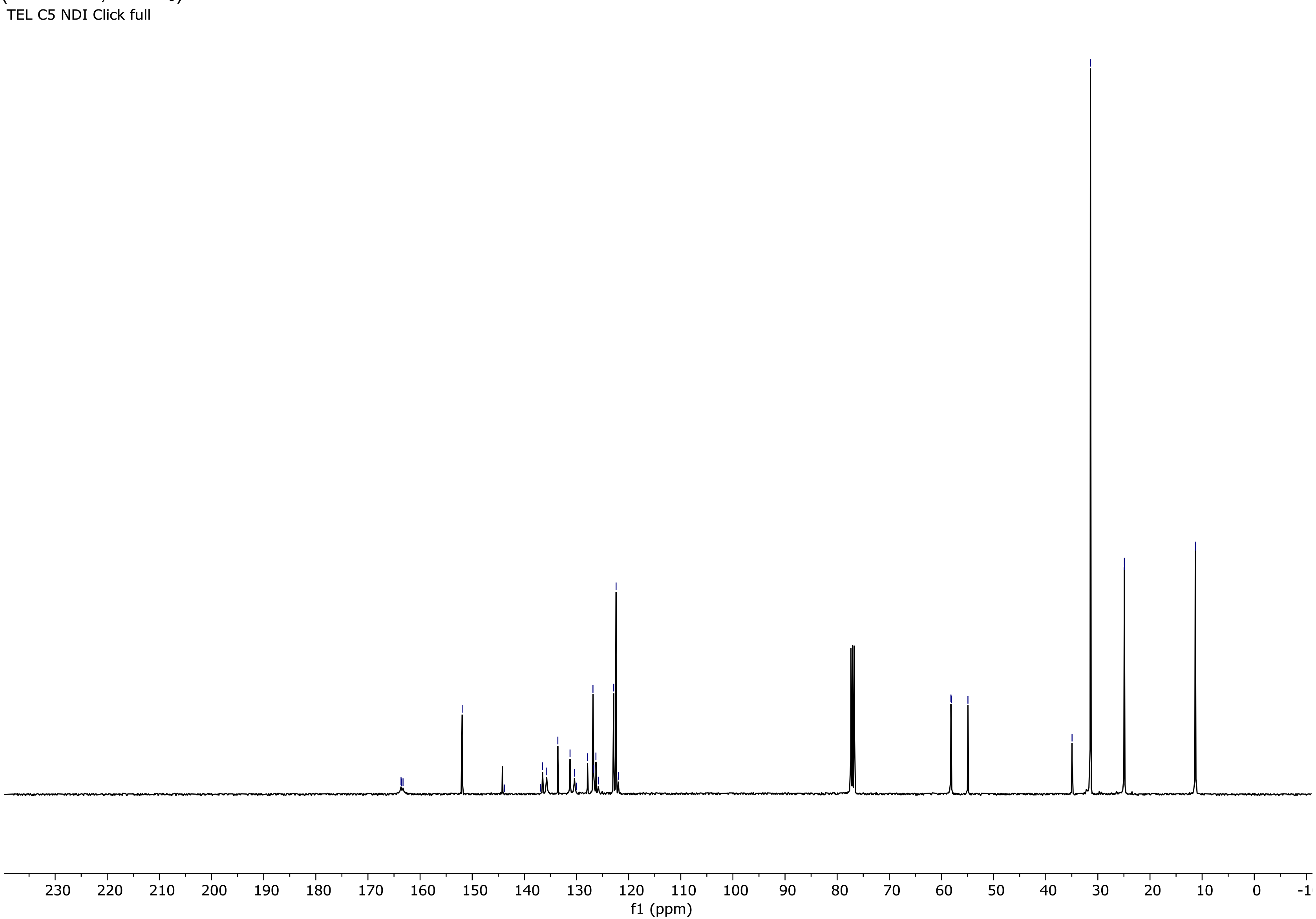

7) Mass Spectra

PDI [2]rotaxane (2)

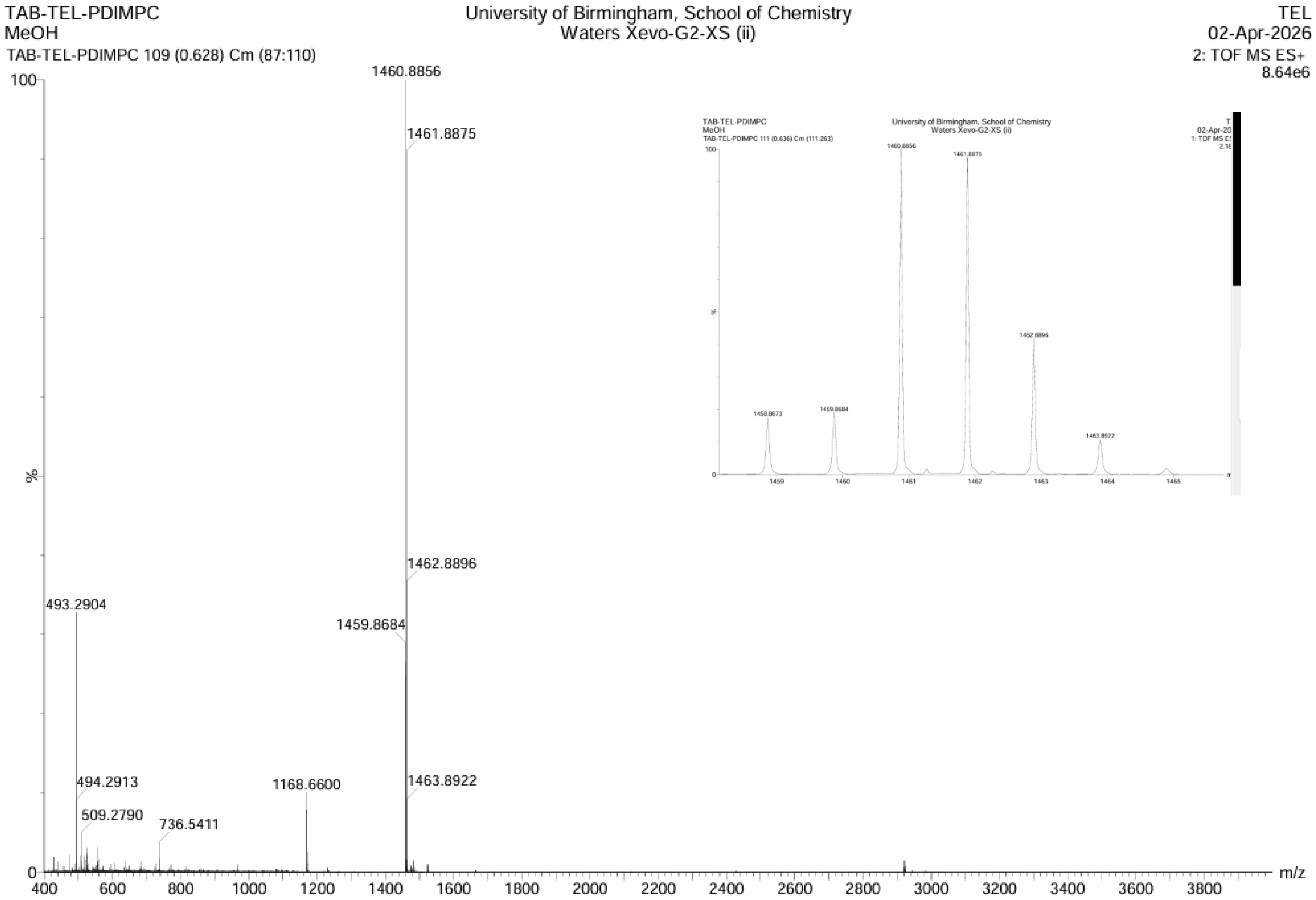

Mono-triazole-C11-PDI (g)

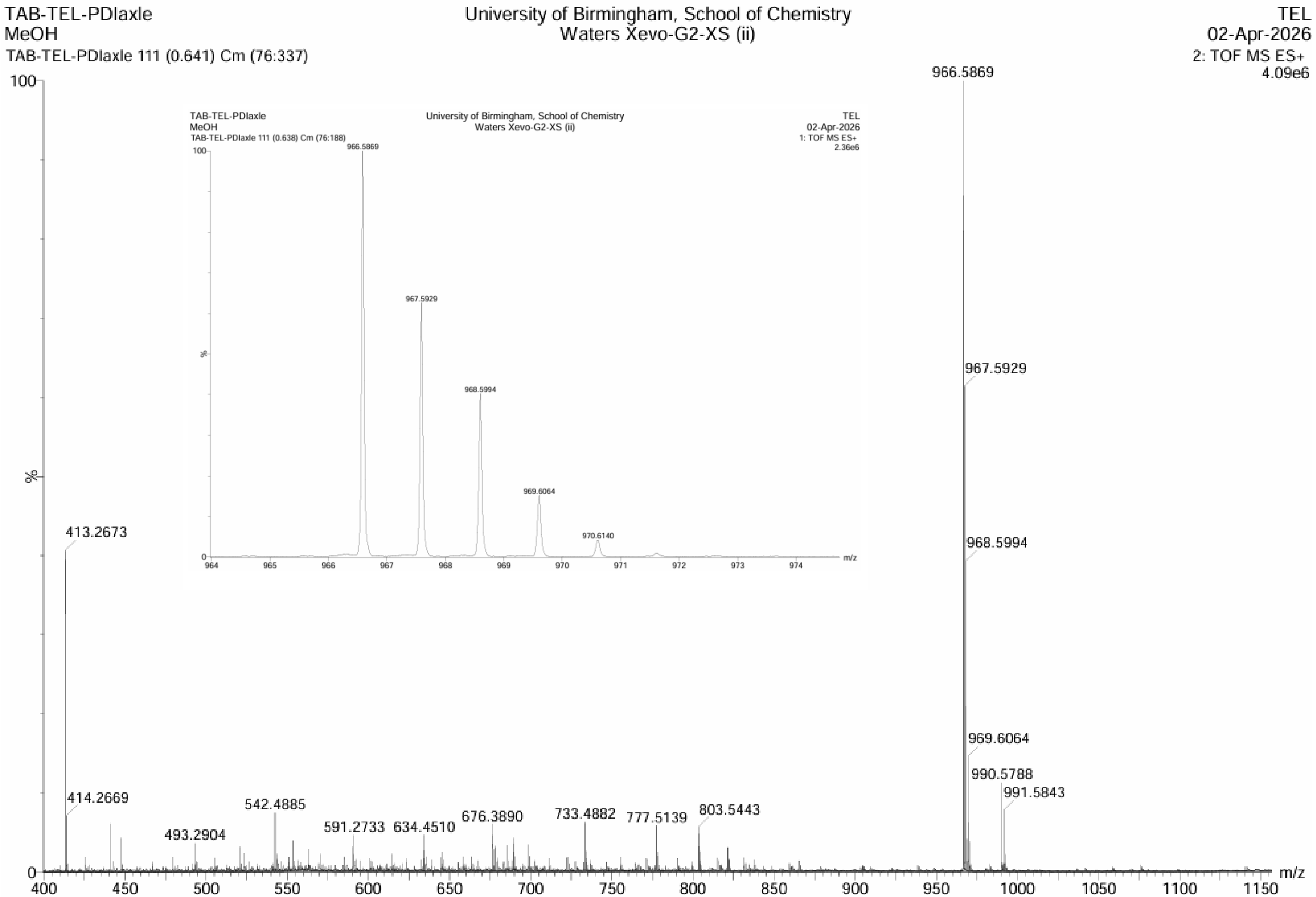

Mono-Br-C5-NDI (j)

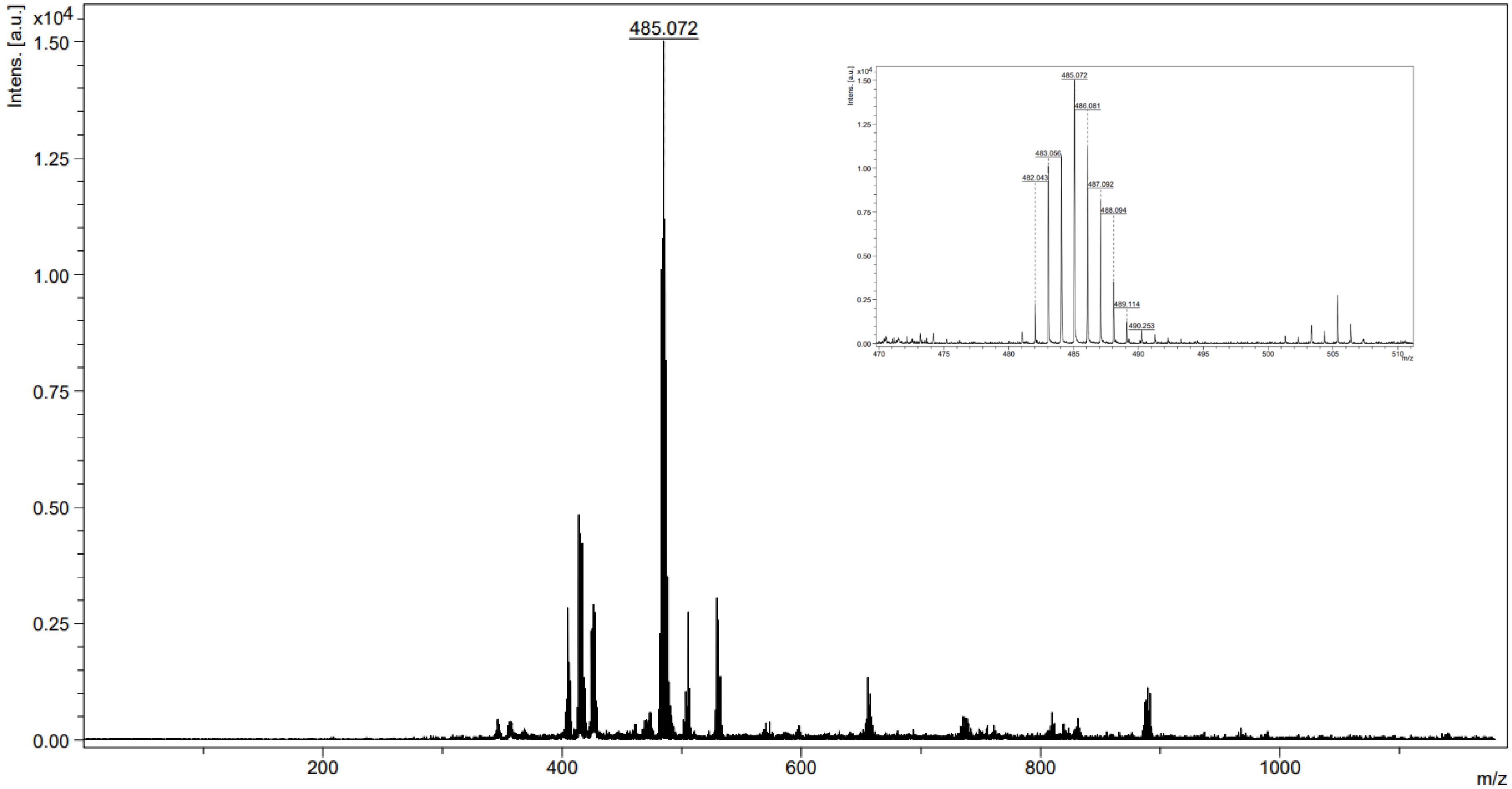

MonoTIPS-C5-NDI (k)

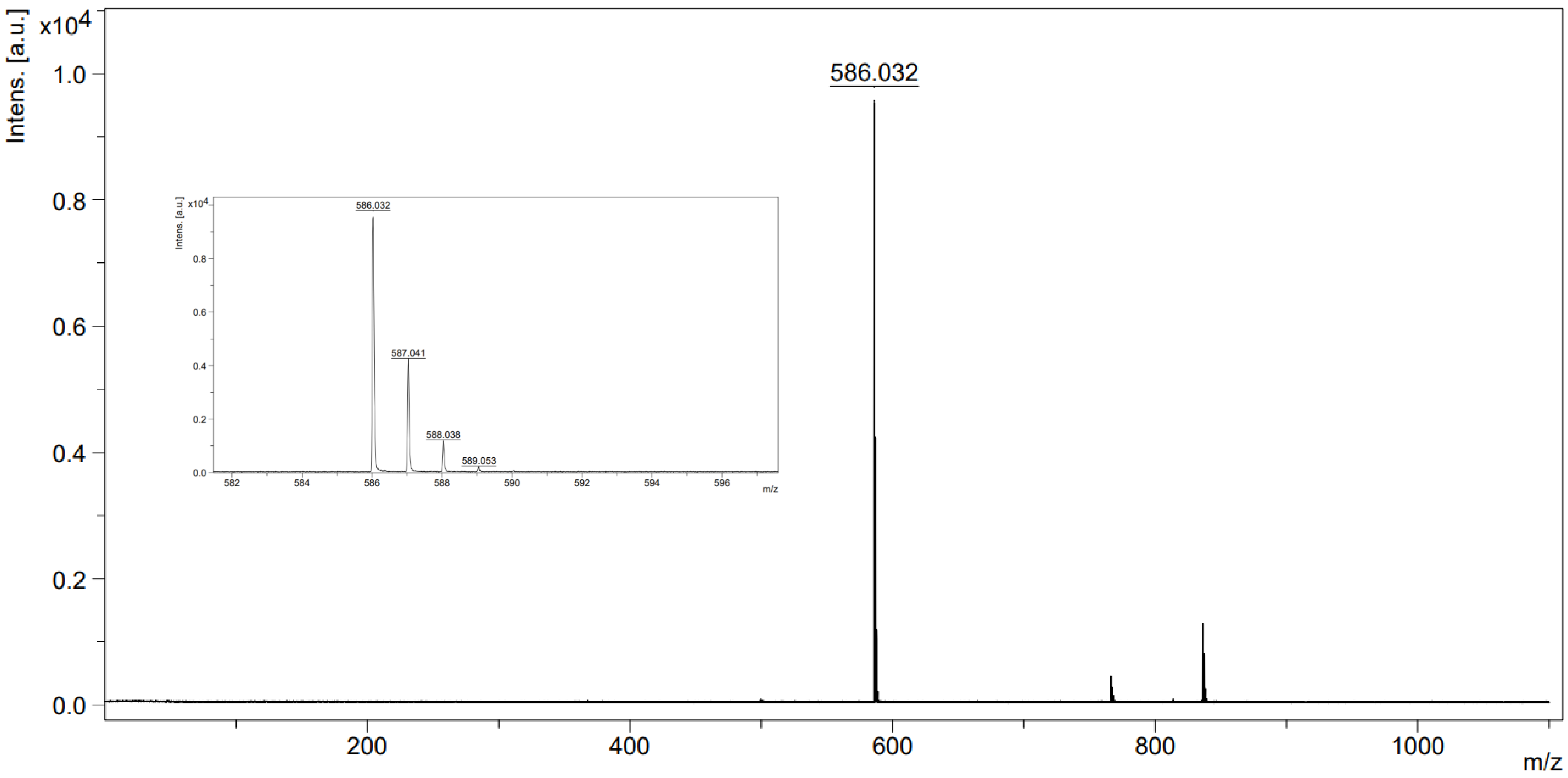

NDI [2]Rotaxane (1)

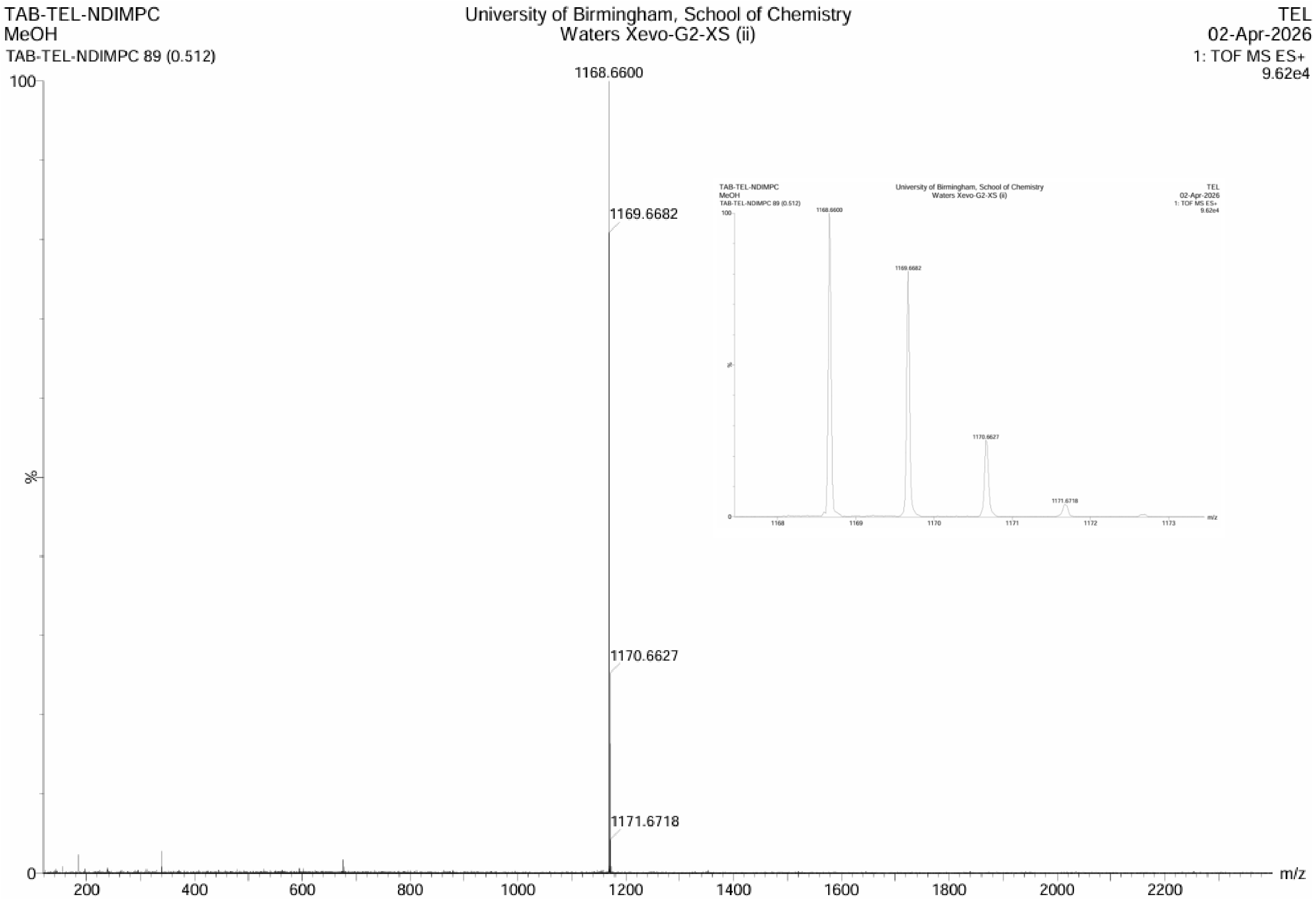

Mono-triazole-C5-NDI (I)

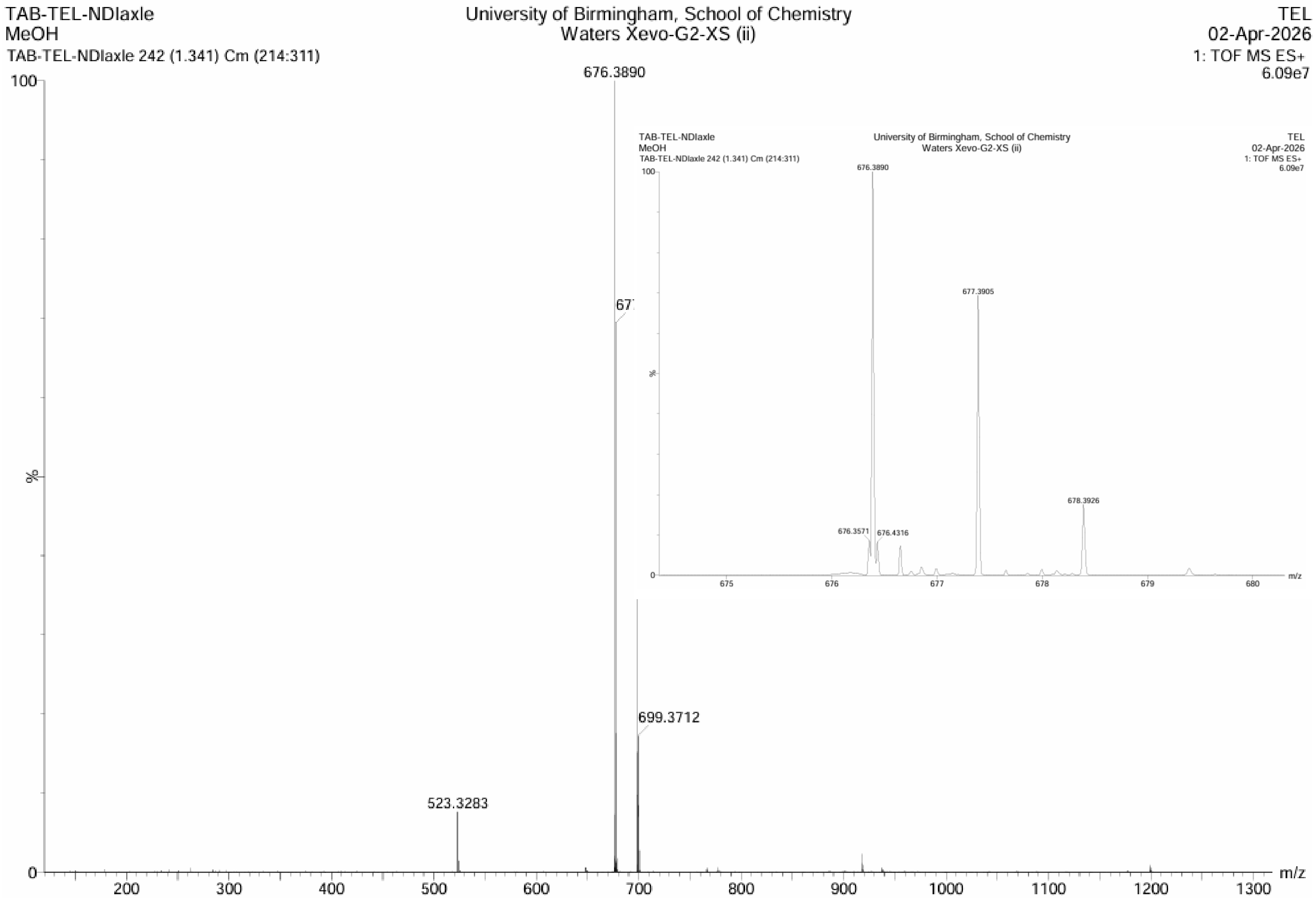

8) References

1. X. Zong, H. Dahiya, Y. Song, X. Liang, S. K. Pandey, H. Xu and G. D. Sharma, *ACS Appl. Energy Mater.*, 2022, **5**, 15624-15637.
2. A. Rodríguez-Rubio, A. Savoini, F. Modicom, P. Butler and S. M. Goldup, *J. Am. Chem. Soc.*, 2022, **144**, 11927-11932.
3. M. Curcio, F. Nicoli, E. Paltrinieri, E. Fois, G. Tabacchi, L. Cavallo, S. Silvi, M. Baroncini and A. Credi, *J. Am. Chem. Soc.*, 2021, **143**, 8046-8055.
4. Y. V. Suseela, M. Sasikumar and T. Govindaraju, *Tetrahedron Lett.*, 2013, **54**, 6314-6318.
5. Z. Chen, A. Lohr, C. R. Saha-Möller and F. Würthner, *Chem. Soc. Rev.*, 2009, **38**, 564-584.
6. F. Würthner, *The Journal of Organic Chemistry*, 2022, **87**, 1602-1615.
7. F. Neese, *WIREs Computational Molecular Science*, 2022, **12**, e1606.
8. M. Bursch, J.-M. Mewes, A. Hansen and S. Grimme, *Angew. Chem. Int. Ed.*, 2022, **61**, e202205735.
9. P. Pracht, F. Bohle and S. Grimme, *Phys. Chem. Chem. Phys.*, 2020, **22**, 7169-7192.
10. C. Bannwarth, S. Ehlert and S. Grimme, *J. Chem. Theory Comput.*, 2019, **15**, 1652-1671.
11. S. Ehlert, M. Stahn, S. Spicher and S. Grimme, *J. Chem. Theory Comput.*, 2021, **17**, 4250-4261.
12. J. G. Brandenburg, C. Bannwarth, A. Hansen and S. Grimme, *J. Chem. Phys.*, 2018, **148**, 064104.
13. F. Weigend and R. Ahlrichs, *Phys. Chem. Chem. Phys.*, 2005, **7**, 3297-3305.
14. J.-D. Chai and M. Head-Gordon, *J. Chem. Phys.*, 2008, **128**, 084106.
15. C. Adamo and V. Barone, *J. Chem. Phys.*, 1999, **110**, 6158-6170.
